# Understanding bovine embryo elongation: a transcriptomic study of trophoblastic vesicles

**DOI:** 10.3389/fphys.2024.1331098

**Published:** 2024-01-29

**Authors:** Séverine A. Degrelle, Fulin Liu, Denis Laloe, Christophe Richard, Daniel Le Bourhis, Marie-Noëlle Rossignol, Isabelle Hue

**Affiliations:** ^1^ Université Paris-Saclay, Université Versailles Saint-Quentin en Yvelines, Institut National de Recherche pour l’Agriculture, l’Alimentation et l’Environnement, Biologie de la Reproduction, Environnement, Epigénétique et Développment, Jouy en Josas, France; ^2^ Inovarion, Paris, France; ^3^ Sichuan Provincial Key Laboratory for Human Disease Gene Study, Center for Medical Genetics, Department of Laboratory Medicine, Sichuan Academy of Medical Sciences and Sichuan Provincial People’s Hospital, University of Electronic Science and Technology, Chengdu, China; ^4^ Université Paris Saclay, INRAE, AgroParisTech, GABI, Domaine de Vilvert, Jouy en Josas, France; ^5^ ELIANCE, Nouzilly, France

**Keywords:** embryo, bovine, elongation, transcriptomics, trophoblastic vesicles

## Abstract

**Background:** During the process of elongation, the embryo increases in size within the uterus, while the extra-embryonic tissues (EETs) develop and differentiate in preparation for implantation. As it grows, the ovoid embryo transforms into a tubular form first and then a filamentous form. This process is directed by numerous genes and pathways, the expression of which may be altered in the case of developmental irregularities such as when the conceptus is shorter than expected or when the embryo develops after splitting. In bovines, efforts to understand the molecular basis of elongation have employed trophoblastic vesicles (TVs)—short tubular EET pieces that lack an embryo—which also elongate *in vivo*. To date, however, we lack molecular analyses of TVs at the ovoid or filamentous stages that might shed light on the expression changes involved.

**Methods:** Following *in vivo* development, we collected bovine conceptuses from the ovoid (D12) to filamentous stages (D18), sectioned them into small pieces with or without their embryonic disc (ED), and then, transferred them to a receptive bovine uterus to assess their elongation abilities. We also grew spherical blastocysts *in vitro* up to D8 and subjected them to the same treatment. Then, we assessed the differences in gene expression between different samples and fully elongating controls at different stages of elongation using a bovine array (10 K) and an extended qPCR array comprising 224 genes across 24 pathways.

**Results:** *In vivo*, TVs elongated more or less depending on the stage at which they had been created and the time spent *in utero*. Their daily elongation rates differed from control EET, with the rates of TVs sometimes resembling those of earlier-stage EET. Overall, the molecular signatures of TVs followed a similar developmental trajectory as intact EET from D12–D18. However, within each stage, TVs and intact EET displayed distinct expression dynamics, some of which were shared with other short epithelial models.

**Conclusion:** Differences between TVs and EET likely result from multiple factors, including a reduction in the length and signaling capabilities of TVs, delayed elongation from inadequate uterine signals, and modified crosstalk between the conceptus and the uterus. These findings confirm that close coordination between uterine, embryonic, and extra-embryonic tissues is required to orchestrate proper elongation and, based on the partial differentiation observed, raise questions about the presence/absence of certain developmental cues or even their asynchronies.

## Introduction

In ruminants, the embryo does not implant at the spherical blastocyst stage, but continues growing and expanding in the uterus for about 2 weeks (D7–D21); during this process of elongation, the embryo progresses through an ovoid and then a tubular form before assuming its final filamentous shape (Greenstein et al., 1958; Betteridge et al., 1980). This is the period in which pregnancy loss is most likely to occur (Walsh et al., 2011), as developmental programs establish the different embryonic and extra-embryonic lineages that continue to differentiate over time (Blomberg et al., 2008; Pfeffer and Pearton, 2012; van Leeuwen et al., 2015; Bernardo et al., 2018; Perez-Gomez et al., 2021; Scatolin et al., 2023). When compared to full blastocysts, split ones also elongate within the uterus, although to a lesser extent (Velasquez et al., 2017). Under *in vitro* conditions, elongation occurs only minimally at best, mm *in vitro versus* cm *in vivo* (Brandao et al., 2004; Vajta et al., 2004), although Akizawa et al. (2021) demonstrated that it is possible to use on-gel cultured blastocysts to perform detailed lineage studies at an early elongating stage (D14).

*In vivo*, the elongation process depends on uterine secretions, a reduction in or the absence of uterine glands preventing elongation (Gray et al., 2002), and requires the establishment of effective, coordinated crosstalk between the uterus and embryo (Clemente et al., 2009). Indeed, the pairings of a younger embryo with an older uterus, or when the conceptus is smaller than expected (in sub-fertile heifers with a less receptive uterine environment, for instance), create inadequacies or asynchronies in this crosstalk (Pope, 1988; Moraes et al., 2018; Mathew et al., 2022). Even between conceptuses of the same age, shorter and longer examples stimulate the uterine environment differently (Sanchez et al., 2019) and exhibit different molecular patterns (Peixoto et al., 2023). Recent work by Jia et al. (2023) generated an atlas of physiological embryo–uterus pairings that extends our knowledge on the pathways at work in *in vivo* ovine pregnancies, refining previous data from bovine and ovine transcriptomic studies (Mamo et al., 2011; Mamo et al., 2012; Ribeiro et al., 2016; Spencer et al., 2019).

Within the uterus, proper pairing between embryonic and extra-embryonic tissues also appears to be essential, with uncoupling being detrimental to developmental outcomes (Degrelle et al., 2011a; Degrelle et al., 2012). Despite this, Ramos-Ibeas et al. (2022) were recently able to generate ovine blastocysts that reached *in vitro* stages resembling the gastrulating patterns of D11 to D12.5 *in vivo*. An interesting finding in that study was that the trophoblast did not proliferate *in vitro* and *in vivo*, but this did not impair subsequent *in vivo* embryonic differentiation. Earlier research also reported the occurrence of *in vitro* gastrulation—in the absence of any extra-embryonic tissues—when human embryonic stem cells were grown on adhesive micropatterns that provided proper signaling molecules (Warmflash et al., 2014). The results of these and other studies suggest that differences in gastrulation across species might not be due to differences between embryonic tissues, but rather their extra-embryonic environments (Van Den Brink and Van Oudenaarden, 2021). Thus, although gastrulation depends on the interactions with the *in vivo* environment, this process can be replicated *in vitro* when the appropriate metabolic and signaling conditions are met.

Can elongation occur in the absence of embryonic or uterine tissues? For embryonic tissues, the answer is yes, since extra-embryonic tissue segments of 0.2–4 mm diameter that lacked any embryonic tissues—called trophoblastic vesicles (TVs)—were observed to elongate *in vivo* when generated from D14 bovine embryos (Heyman et al., 1984). With respect to the uterus, although the answer is no, TVs were generated from D12 ovine embryos elongated only *in vivo* (up to 180 mm), not *in vitro* (Flechon et al., 1986). Although the *in vivo*-elongated TVs did not match the size of *in vivo* controls at day 17 (up to 250 mm; Flechon et al., 1986), their growth was significant, reaching 5 to 10 or even 100 times their size at transfer. Since then, TVs have been derived from *in vitro*-produced blastocysts (D7 and D8), and have sometimes been used for their secretions (Stojkovic et al., 1997; Nagai et al., 2009).

However, to date, trophoblastic vesicles have not been produced at other stages, and molecular studies of these tissues have focused only on their trophectoderm (Pillai et al., 2019). To fill this gap, we generated bovine TVs at D8, D12, and D15 and transferred them *in vivo* for 3, 6, or 9 days, respectively, in order to compare their elongation rates and molecular profiles at different times after the transfer. This first-of-its-kind study investigates the similarities and differences between the elongation rates and molecular profiles of TVs and those of the whole conceptuses, as well as patterns described for other models of elongation.

## Materials and methods

### Animals

Animal use and care were carried out in accordance with the International Guiding Principles for Biomedical Research Involving Animals at the INRAE experimental farm (registered under N° FRTB910 in the national registry). The protocols for these studies were approved by the local ethics committee (Comité d’Ethique en Expérimentation Animale du Centre INRAE de Jouy-en-Josas et AgroParisTech (COMETHEA), registered as 12/084 and 12/086 in the National Ethics Committee Registry).

### Day 8 *in vitro*-produced embryos

Oocytes were matured *in vitro*, as described in the work of Khan et al. (2012). Twenty-four hours after the onset of maturation, metaphase II oocytes were incubated with heparin-capacitated, thawed spermatozoa in a TALP medium for 18 h, according to the standard *in vitro* fertilization technique of the laboratory (detailed in the work of Comizzoli et al., 2000). Following IVP, embryos were cultured until day 8.

### Day 12 and day 15 *in vivo*-produced embryos

For *in vivo* embryo production, cows were bred through artificial insemination (AI) following induced estrus. Synchronization was carried out via a superovulation treatment (eight degressive doses of Stimufol^®^, Reprobiol, Soiron-Pepinster, Belgium; one Stimufol dose contained 500 mg porcine FSH and 100 mg porcine LH), as detailed in the work of Richard et al. (2015).

AI was conducted using one batch of frozen sperm from a single Holstein bull; the same batch was used for the *in vitro*-produced embryos. Bovine embryos were collected on day 12 and day 15 after insemination via non-surgical recovery by flushing the uterine horns with PBS (Richard et al., 2015). The flushing solution (Euroflush, IMV Technologies, France) was heated to 38°C at the beginning of the experiment and maintained at 30°C in a thermostatic box throughout the collection period.

### TV production

In this study, the D8 blastocysts used for TV production were produced *in vitro*, while D12 and D15 embryos were produced *in vivo*. This decision was made for two reasons: 1) *in vivo* D8 blastocysts were expensive to obtain a sufficient number for analysis, and 2) the time required to collect and transport *in vivo* D8 blastocysts from the farm to the lab (80 km distance, ca. 2 h) for dissection reduced the chances of successful TV development *in vivo*. Thus, each group of embryos was dissected at the location where they were produced, in the lab for *in vitro* D8 blastocysts and on the farm for D12 and D15 embryos collected from donor cows.

### *In vitro*-developed blastocysts

*In vitro*-produced D8 blastocysts were bisected with and without the inner cell mass (ICM+ and ICM-). After 2 h in the B2 medium (B2 as in the work of Menck et al., 1997), ICM- halves were transferred to recipient cows and left there until D12, whereas ICM+ halves were transferred to other recipient cows and recovered on D15, D18, or D21.

### *In vivo*-developed embryos at D12 and D15

D12 *in vivo*-produced embryos were flushed out following the procedure used in previous studies (Heyman et al., 1984; Flechon et al., 1986). Ovoids were bisected within and without the embryonic disc (ED+ and ED-). These were incubated in a B2 medium for 2 h and then transferred to new recipient cows until day 15 or day 21 for the ED+ halves.

D15 *in vivo*-developed embryos were flushed out following the same procedure used above. The tubular embryos were cut into pieces, mostly without an embryonic disc. After incubation in a B2 medium for 2 h, they were transferred to recipient cows for 3 days (until D18).

### Tissue sectioning for TV production

Sectioning was performed in a Petri dish using a scalpel under a binocular microscope for D12 and D15 embryos or with a microscalpel and micromanipulators for D8 blastocysts. Under an inverted microscope equipped with micromanipulators, the embryos were placed in an embryo-holding medium (EHM, IMV Technologies, France) and stabilized by using a holding pipette. A homemade microblade was slowly pressed against the middle of the embryo, and the embryo was cut into two hemi-embryos. The hemi-embryos were then washed in the EHM and loaded into a sterile straw for embryo transfer.

### Recipient cows

As recipients for the transfers, we used 11 heifers from two common breeds (Normande, *n* = 8; and Holstein, *n* = 3) that were about 3 years old, were not lactating at the time of transfers, and had never previously experienced lactation. Each animal was selected for synchronization following the observation of two heat cycles; they were, thus, cycling, with functional reproductive organs.

Estrus was induced in each recipient heifer using a Crestar implant (MSD Santé Animale INTERVET, Beaucouzé, France), and the successful induction of estrus was monitored and recorded five times a day by experienced animal care workers. Day 0 was synchronized between insemination and *in vitro* fertilization protocols, and transfers were scheduled accordingly, as described in the work of Richard et al. (2015).

### Tissue panel

**FIGURE 1:**
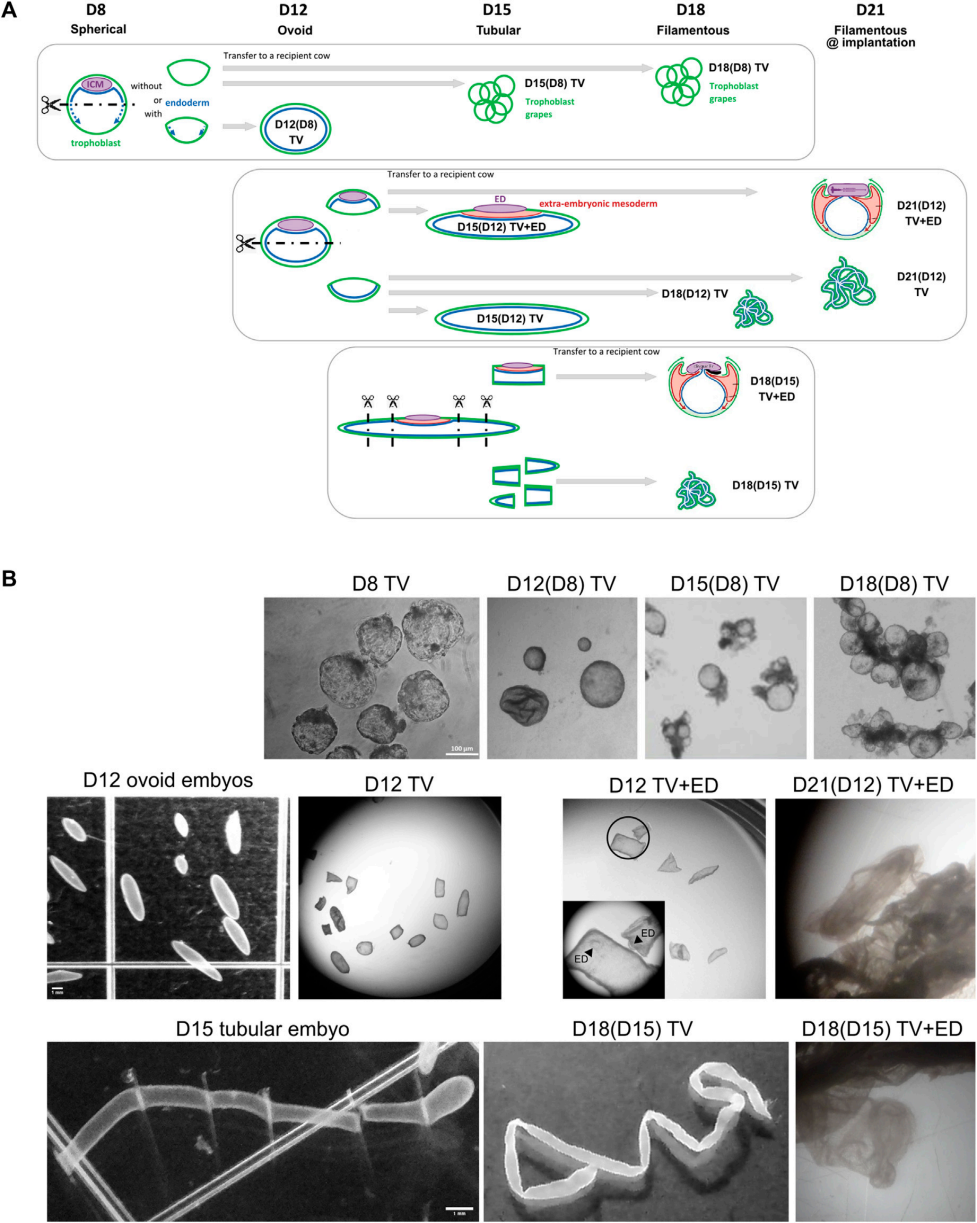
Experimental design for the isolation of bovine TVs and embryonic tissues at successive developmental stages. **(A)** *In vitro*-produced blastocysts were bisected at day 8 to generate TVs (D8 TV). D8 TVs were transferred to recipient cows and recovered at day 12, 15, or 18. *In vivo*-derived ovoid embryos were microdissected at day 12 to isolate TVs with trophoblast and endoderm (D12 TV) and embryo halves with the embryonic disc (TV+ED). D12 TVs were transferred to recipient uteruses and recovered at day 15, 18, 21, and even 25 (not shown). *In vivo*-derived tubular embryos were dissected at day 15 to generate TVs and embryonic discs. D15 TVs were transferred to recipient cows and recovered at day 18. Isolated tissues were used for transcriptomic analyses. The developmental morphology of bovine conceptuses at days 8, 12, 15, 18, and 21 is depicted above each timeline. **(B)** Bright-field images of bovine embryos or trophoblastic vesicles at different days of gestation (D8, D12, D15, D18, and D21).

**FIGURE 2:**
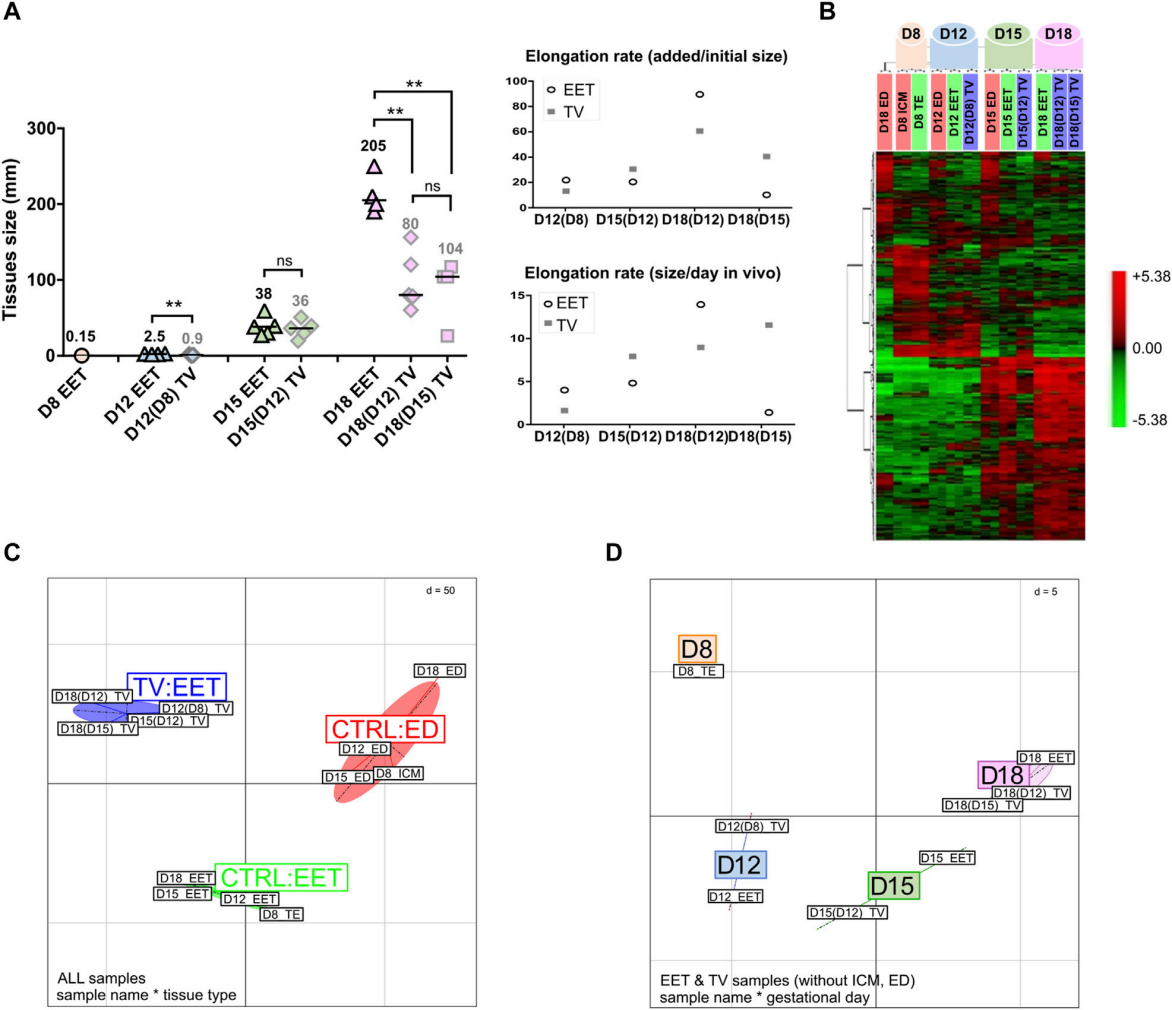
Transcriptomic profiling of bovine TVs and EET controls during the key stages of conceptus elongation. **(A)** Estimations of the elongation rates of TVs and EET controls. TV sizes at the time of transfer *in utero* were 0.125 mm at D8, 1.5 mm at D12 and D14, and 3 mm at D15. Elongation rates of EET and TVs are estimated in each stage on a per-mm basis, which corresponds to mm added per initial mm (e.g., a size of 2 mm would be represented as 1 mm per initial mm), and on a per-day basis (mm added per day in the uterine cavity). EET stages are associated with sizes using the following standard: ovoid (<30 mm), early tubular (30–50 mm), tubular (50–100 mm), early filamentous (100–150 mm), and filamentous (>150 mm). **(B)** Hierarchical clustering analysis of differential gene expression between bovine TVs and control tissues (ED and EET) during elongation. Samples were collected at days 8, 12, 15, and 18 after insemination (*in vivo*) or after fertilization (*in vitro*). Differentially expressed genes were identified by TMEV (*p* < 0.05) and clustered using PermutMatrix. Each colored cell represents a gene value according to a color scale that we added at the right of panel B (red: high expression and green: low expression) **(C)** BCA of global gene expression profiles in elongating bovine TVs and control tissues (CTRL ED and CRTL EET). Samples cluster primarily along the *x*-axis by the developmental stage (D8, D12, D15, and D18) and secondarily along the *y*-axis by the tissue type (ED, EET, and TVs). **(D)** 3D projection of BCA comparing the global gene expression profiles of bovine TVs and intact EET during elongation. Samples cluster primarily along the *x*-axis by the developmental stage (D8, D12, D15, and D18). In panels C and D, Euclidean distance (d) for normalized and centered data is given.

**FIGURE 3:**
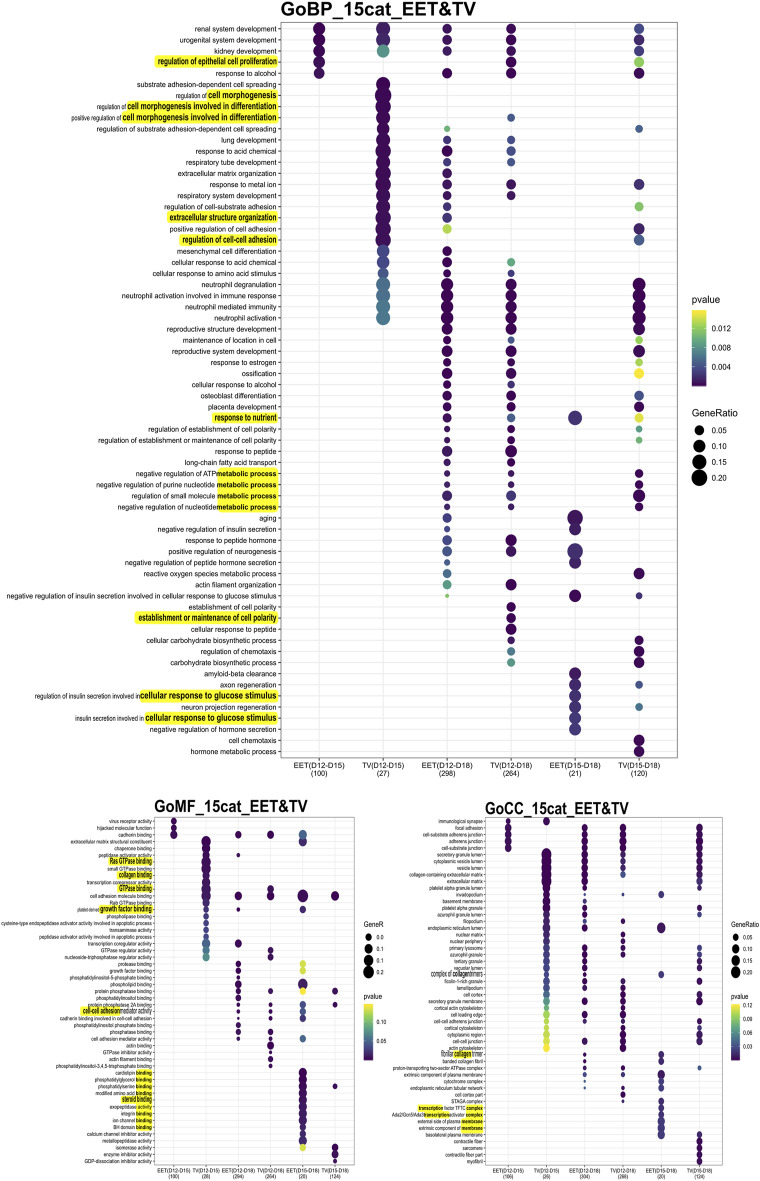
Comprehensive analysis of differential gene expression and functional enrichment across developmental stages. GO enrichment analysis of differentially expressed genes (DEGs) identified in comparisons between bovine TVs and intact EET controls (CTRL) during elongation. DEGs were identified in comparisons of tissues between D12 and D15, D12 and D18, and D15 and D18 of gestation. The DEG numbers are provided on a per-contrast basis, between parentheses, and given in Supplementary Table S3B. Enriched GO terms (adj. *p* < 0.05) are shown for the categories BPs, MFs, and CCs. To the right of the panel, the scales for the *p*-value and gene ratio for each GO term are observed. Underlined are the terms on which we focused.

**FIGURE 4:**
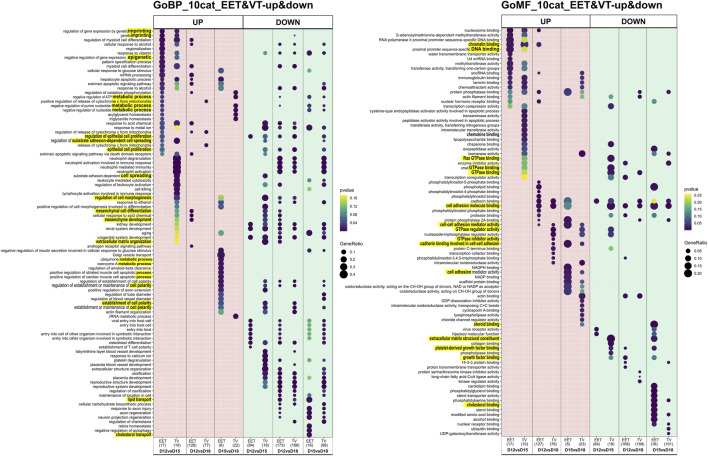
Differential gene expression and functional enrichment in bovine trophoblastic vesicles and extra-embryonic tissues across stages. GO enrichment analysis of DEGs that were up- or downregulated in bovine TVs compared to intact EET controls. DEGs were identified from comparisons of different gestational stages (D12 vs. D15, D12 vs. D18, and D15 vs. D18). Significantly enriched GO terms (adj. *p* < 0.05) are shown separately for up- and downregulated DEGs in TVs and EET. DEG numbers, between parentheses, are given in Supplementary Table S3B. BP, biological process; MF, molecular function. Underlined are the terms on which we focused.

**FIGURE 5:**
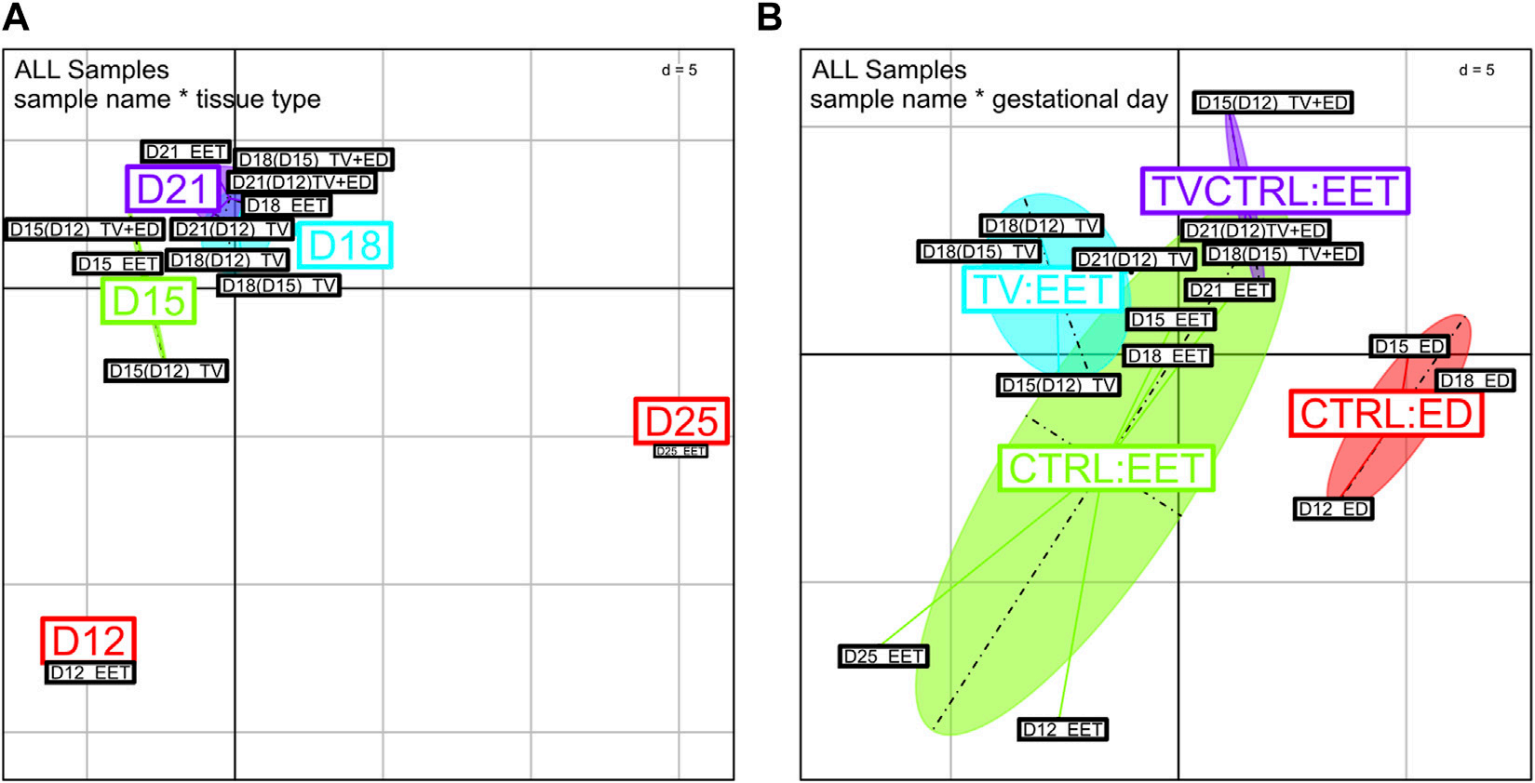
BCA of global gene expression profiles from high-throughput qPCR data. Comparison of global gene expression patterns between bovine trophoblastic vesicles (TV and TV+ED = TV-CTRL) and control tissues (CTRL ED and CTRL EET) across various developmental stages (D12, D15, D18, D21, and D25). Samples were analyzed using OpenArray qPCR and clustered based on Rq values; any missing values were replaced with the mean expression level per sample group. Each point in the BCA represents a sample, with different colors indicating **(A)** gestational days or **(B)** tissue types. **(D)** Euclidean distance (d) for normalized and centered data.

**FIGURE 6:**
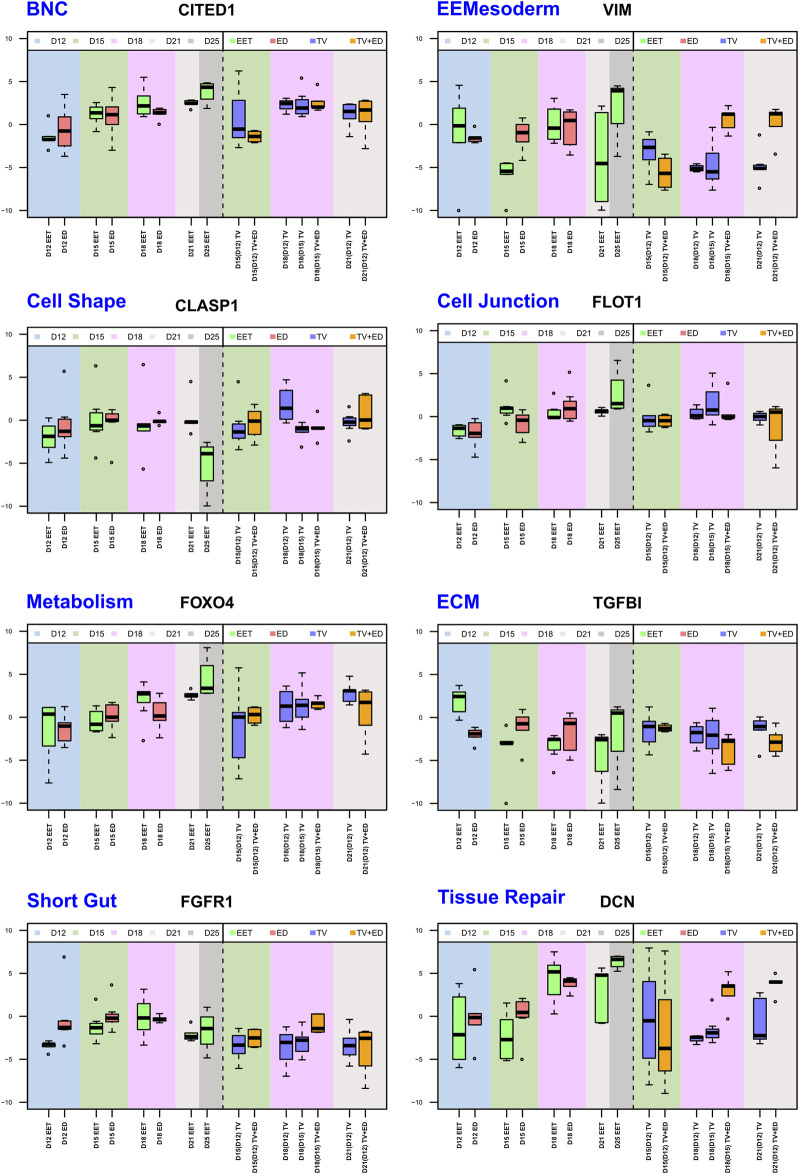
Comparative analysis of selected gene expression profiles across stages (D12, D15, D18, D21, and D25) and tissue types. Eight boxplots that highlight differential expression patterns across tissue types—EETs, EDs, TVs, and TVs containing the ED (TV+ED = CTRL TV)—for eight genes from different pathways. Each boxplot illustrates the median, interquartile range, and potential outliers of log2 (RQ) values, providing a visual representation of the transcriptomic landscape within each biological pathway and tissue type. The data span five gestational days (D12, D15, D18, D21, and D25), capturing the temporal dynamics of gene expression throughout development. The full analysis, comprising 224 genes from 24 biological pathways, can be found in Supplementary Figure S3.

From the entire design, several kinds of trophoblastic vesicles were collected (*n* = 10, Figure 1), with or without embryonic tissues, inner cell mass at D8 (ICM), germ disc at D12, or embryonic disc (ED) at D15. As previously reported, the germ disc is an early form of the embryonic disc (Degrelle et al., 2005). To ease the reading of the figures linked to molecular studies, the germ disc was an “ED” (Figures 1A,B; Figure 2B; Figure 5; Figure 6, Supplementary Figure S1). Referred to as CTRL tissues within molecular studies (10 K array, OpenArray), extra-embryonic tissues from D8 to D21 have been used: D8-TE (Figure 2) or D12 to D21 EET (Figure 2; Figure 3; Figure 4; Figure 5; Figure 6). D25 EET (Figure 5; Figure 6) were not collected therein but collected from previous studies (Supplementary Table S1).

### Microarray analysis

Total RNA was isolated using the RNeasy Mini Kit, incorporating in-column DNase digestion (QIAGEN); T7 linear amplification was carried out using the MessageAmp aRNA Kit (Ambion) (Degrelle et al., 2011a; Degrelle et al., 2012). Array hybridization was conducted as described in the work of Degrelle et al. (2011a) and Degrelle et al. (2012). In brief, 500 ng of amplified RNA (aRNA) was labeled with [α-33P] dATP through reverse transcription and hybridized to an INRA bovine 10 K array (GPL7417). Then, arrays were exposed to phosphor screens for 7 days. Hybridization signals were quantified using ImaGene 5.5 software (BioDiscovery) on the PICT/ICE platform. These datasets are accessible in the Gene Expression Omnibus database (http://www.ncbi.nlm.nih.gov/geo, GSE146768). Data were log-transformed and mean-centered. Statistical and clustering analyses were conducted using TIGR MeV 4.7.3 (MultiExperiment Viewer program, http://www.tm4.org/mev.html). Differentially expressed genes (DEGs) between cell types were assessed using one-way ANOVA, with an adjustment based on Bonferroni correction and significance at *p* < 0.05 (Supplementary Table S2). Unsupervised hierarchical clustering, based on Euclidean distance and complete linkage, was performed on the significant gene expression differences across all studied groups using PermutMatrix (Caraux and Pinloche, 2005), as previously described in the work of Valour et al., 2014. Further investigations were conducted using the between-class analysis of variance (BCA) to work on averaged expression values per embryo group, as in the work of Hue et al., 2018. Different effects were analyzed to characterize their variability including tissue types and gestational days. Data processing further used the methods detailed in Liu et al. (2020). In brief, the threshold to identify the up- and downregulated genes per statistical comparison was set to a fold change of 1.5, and the Gene Ontology (GO) terms (or KEGG pathways) associated with the differential expressions (all DEGs, as well as up- or downregulated DEGs) were recovered using ClueGO. Then, GO terms were compared between groups using the R package clusterProfiler (version 3.9). Then, 10 to 15 category terms per group were selected to include them into the charts. *p*-values lower than 0.05 identified significant enrichment.

### OpenArray high-throughput RT–qPCR analysis

In addition, we analyzed differences in gene expression using a customized TaqMan OpenArray Real-Time PCR System (Life Technologies). We designed a TaqMan OpenArray based on our previous research and a thorough review of the literature. In total, 218 genes were selected, plus 6 endogenous control genes (GAPDH, HMBS, HPRT1, RPLP0, SDHA, and YWHAZ). The candidate genes selected for this project represented various functions of extra-embryonic tissues and different cellular lineages; they are primarily involved in the regulation of cell proliferation and differentiation, cellular interactions, lipid synthesis and metabolism, and tissue repair of the epithelium (Supplementary Table S4). Total RNA from control tissues (*n* = 55) and TVs (*n* = 40) (Supplementary Table S1) was extracted using an RNeasy Micro or Mini RNA Isolation Kit (QIAGEN) following the manufacturer’s instructions and quantified using the NanoDrop Spectrophotometer. The quality of all RNAs was checked using an Agilent 2100 Bioanalyzer (Agilent Technologies, Santa Clara, CA, United States), with an average RNA integrity number that is superior to 8.5 on a scale of 0–10. For each sample, 50 ng of RNA was reverse-transcribed using SuperScript IV VILO Master Mix with ezDNase Enzyme and preamplified according to the manufacturer’s instructions. Then, preamplified cDNA was mixed with the TaqMan OpenArray Real-Time PCR Master Mix and loaded onto an OpenArray plate using the AccuFill™ System. Standard cycling conditions were used as recommended by the manufacturer. Thermal cycling and fluorescence detection were performed using the QuantStudio 12 K Flex Real-Time PCR System (Applied Biosystems). Data were extracted using OpenArray real-time qPCR analysis software (Applied Biosystems). The fold change in gene expression [relative quantification (Rq)] was calculated using the comparative 2−ΔΔCq method with global normalization of all gene expression data. Rq values data obtained from TaqMan OpenArray experiments were log-transformed (log2) and analyzed using BCA from the ade4 R package, as in the work of Taberlet et al. (2012). The effects of interest to characterize the variability between the groups included tissue types and gestational days. To map all these data across tissue types and gestational days, boxplots were presented.

## Results

### Features of elongating TVs and EET

TVs that were generated from *in vitro*-produced bovine blastocysts at D8 did not elongate following their transfer to a recipient uterus; the resulting trophoblastic vesicles remained of similar size at D12 (4 days *in utero*), D15 (7 days), and D18 (11 days; Figures 1A,B). In contrast, trophoblastic vesicles created from D12 embryos did elongate during the 3–9 days spent *in utero* (Figure 1A), with a median length that doubled between D15 and D18 (36 mm vs. 80 mm; Figure 2A). Compared on a per-day basis, D12-derived TVs demonstrated similar patterns of elongation at both D15 and D18 (the same increase of approximately 8 mm per day in the uterus). Finally, TVs produced from D15 EET also elongated up to D18 (104 mm, Figure 2A), although less efficiently than those derived from D12 EET (34 mm per initial mm in D15-derived TV at D18, *versus* 53 mm/mm in D12-derived TV at D18). Compared to EET that developed fully *in vivo*, TV elongation rates clearly differed. Among the control EET, there was little difference in elongation rates between ovoid D12 and early tubular D15 samples (16 and 14 mm/mm and 4–5 mm/day when comparing each to their preceding stage). However, at D18, D12-compared EET and D12-derived TVs had the highest elongation rates among any of the tested samples (81 and 53 mm/mm, respectively). Moreover, on a per-day basis, D15-derived D18 TVs elongated similarly to D12-compared D18 EET (11.2 *versus* 13.5 mm/day, respectively), which could suggest that the process starts later in TVs. Finally, D12 or D15 embryonic discs surrounded by a short piece of EET (TV+ED) were also found to differentiate following the transfer *in utero* for 3–9 days (Figure 1A); at D18, we observed mesoderm derivatives in D15-derived TV+ED, while at D21, somites and headfolds were apparent in D12-derived TV+ED (Figure 1B). Altogether, these data demonstrate the quality of the *in vivo* embryos that we used to generate EDs and TVs, the quality of their recipient cows, and complement earlier findings on D14 bovine or D12 ovine TVs (Heyman et al., 1984; Flechon et al., 1986).

### TV, EET, and ED molecular patterns

Transcriptomic profiling on a bovine array revealed different patterns between TVs and control tissues (EET and ED), with 533 identified DEGs (adjusted *p*-value <0.05; Supplementary Table S3A). Consistent with their distinct developmental programs, the embryonic discs exhibited divergent expression profiles from EET (Figure 2B). Instead, EET and TV samples primarily clustered by day from day 8 to day 18, indicating that the overall signature of TVs followed a developmental trajectory similar to that of elongating EET (hierarchical clustering, Figure 2B; between-class analysis, Figure 2D), even with or without embryonic tissues: ICM or ED (heatmaps, Supplementary Figure S1). Nonetheless, as elongation proceeded, TVs demonstrated progressive shifts in gene expression and began to cluster separately from the control tissues (ED and EET), suggesting that TVs also have unique gene expression dynamics (Figure 2C, BCA). Particularly, samples from D12-derived TVs collected at D15 or D18 [TV D15(12); TV D18(12)] were found to cluster with D15-derived TVs sampled on D18 [TV D18(15)] rather than with control EETs (D15 or D18), and D12 TVs derived from D8 blastocysts clustered closer to their precursor, the D8 TE, than to D12 EET (heatmaps with or without discs, Supplementary Figure S1). Altogether, it seems that TVs derived from D12 or D15 EET experienced compensatory changes that increased their resemblance to each other at D18, whereas TVs derived from D8 blastocysts did not progress in their development, probably due to the absence of necessary cues. To examine this further, we next investigated the pathways involved in order to identify the common temporal patterns at play and tissue-specific differences in the molecular regulation of elongation between TVs and intact conceptuses.

### Differential gene expression in TVs and EET

The patterns of differential gene expression were identified among EET samples at different stages of elongation, and these were partly shared with elongating TVs (Supplementary Table S3). Unsurprisingly, the largest number of DEGs (*n* = 470) was identified between EET samples at the two extremes of development, namely, the blastocyst stage (D8) and the filamentous conceptus (D18). In terms of gene expression, the earliest stages of elongation (ovoid and tubular) appeared to be quite distinct from each other (D12 vs. D15: 132 DEGs), but these differences were much more muted between the tubular and filamentous stages (D15 vs. D18: 27 DEGs). Among the TV samples, the greatest differences in expression were identified between D12(D8) and D18 samples and between D15(D12) and D18 samples (339 and 165 DEGs, respectively; Supplementary Table S3A). Instead, D12(D8) and D15(D12) TVs appeared to be quite similar to each other, with only 35 DEGs, of which 23 were shared with the comparison between D12 and D15 EET. Altogether, the most transcriptomically similar conditions appeared to be in D15 and D18 EET (27 DEGs), D12(D8) and D15(D12) TVs (35 DEGs), and TVs and EET at D12 (16 DEGs) or D15 (23 DEGs). To further investigate these patterns, we characterized the overlapping gene networks implicated (from two to seven depending on the conditions compared; ingenuity analyses, not shown) and identified the functional enrichment shared (or not) among different comparisons using GO analysis. Few biological processes (BPs), molecular functions (MFs), and cellular components (CCs) appeared to be specific for TVs between D12 and D15 (BP: cell morphogenesis in differentiation; MF: GTPase, collagen binding) and EETs between D15 and D18 (BP: cellular response to glucose stimulus; MF: numerous binding activities; and CC: transcription complex and plasma membrane components). However, few were rather differential (Figure 3). For example, genes associated with the function “epithelial cell proliferation” appeared to be differentially expressed in TVs between D12 and D18, which may reflect a delay compared to EET, in which the same difference was detected between D12 and D15. In contrast, EET exhibited specific enrichment for “steroid binding” at D18 compared to D15, a difference that was not observed in TVs (Figure 3). Among TVs and EETs, functions associated with “imprinting, epigenetic (BP) or chromatin binding (MF)” were found to only be upregulated at D15 compared to D12, whereas “cell adhesion molecule binding (MF)” was upregulated at D18 compared to D12, but downregulated from D15 to D18 (Figure 4). When we examined the overall patterns of up- and downregulation, TV-specific upregulated functions included “cell morphogenesis” (BP) or “GTPase binding” (MF, Figure 4) together with “metabolic” functions (KEGG pathways, Supplementary Figure S2), whereas downregulated terms were more associated with “cell spreading,” “mesenchyme” (BP, Figure 4), “ECM constituents,” “growth factor-binding” (PDGF, for instance; MF, Figure 4), or “signaling” pathways (TNF and PPAR, for instance, Supplementary Figure S2). All pathways and functions that make sense with developmental processes.

### In-depth transcriptomic profiling of TVs, ED, TV+ED, and EET

To obtain a more detailed understanding of the patterns of gene expression involved in these tissues and processes, we performed real-time qPCR profiling on a set of 218 genes (plus 6 control genes) identified from a search of the literature. Among these, 21 genes were highlighted in the DEG analysis described above (Supplementary Table S3C). The genes of interest are involved in pathways relevant to lineage specification, morphogenesis, proliferation, differentiation, metabolism, or epithelium repair (Supplementary Table S4, Supplementary Figure S3), some of which are also identified above (Figure 3, Figure 4). In addition to the 12 samples we profiled on the bovine 10 K array, we also included 8 additional samples (*n* = 8, Supplementary Table S1) for qPCR profiling on the OpenArray (OA) platform. These included EET at D21 and D25 to evaluate EET differentiation and TVs with an embryonic disc, transferred like other TV samples and grown *in utero* for the same amount of time (3, 6, or 9 days), to assess embryonic/extra-embryonic lineages. The results revealed a clear clustering of samples based on the tissue type (Figure 5), with distinct patterns of segregation among embryonic discs (CTRL:ED), extra-embryonic tissues (CTRL:EET), TVs (TV:EET), and TVs with embryonic discs (TVCTRL:EET). This finding confirms that i) TV samples exhibit transcriptional profiles that diverge from those of intact EET (controls) at each stage (D12 to D25), and ii) the presence of an embryonic disc in TVs shifts gene expression patterns closer toward those of intact embryos. Indeed, D15(D12) TV+ED samples were found to cluster closer to D15 EET (control) than to D15(D12) TV (Figure 5A). However, the reverse was true at D21: D21(D12) TV+ED clustered closer to D21(D12) TV than to D21 EET (control). This could suggest that the effects of embryonic signaling on gene expression in EET diminish over time and are potentially outweighed by the influence of uterine signaling at D21.

### Key developmental profiles in TVs, ED, TV+ED, and EET

According to the between-class analysis, the largest cluster was CTRL EET (Figure 5B), which comprised samples from all stages of the elongation process (D12–D21 and even D25). Comparatively, the other clusters were less variable, with the TV+ED and ED groups being the most “focused,” likely due to the absence of the D21/D25 samples (included only in Figure 5A). This same pattern was evident with respect to the expression of individual pathways (Figure 6; Supplementary Figure S3). By combining our DEG analysis with OA data, we were able to detect distinct temporal patterns in EET, TVs, and TV+ED. For example, genes associated with embryonic tissues, such as POU5F1, NANOG, and SOX2, demonstrated high expression in early EET but declined over time (D12 to D18) compared to levels in the corresponding EDs (increase or steady state), reflecting the loss of pluripotency as EET differentiates. Endoderm genes, such as FN1, GATA4, GATA6, PLET1, and HNF4A, also exhibited early expression in EET (D12–D15). Trophoblast genes, including ASCL2, CDX2, ETS2, FGFR2, GATA3, HOPX, IFNT, PAG11, and SSLP1, were expressed at progressively higher levels in EET (D12 to D15, or D12 to D18) and maintained high expression over time in early TVs and TV+ED (D12- or D15-derived), highlighting their critical roles in trophoblast specification. Consistent with the patterns of trophoblast fusion, two genes linked with trophoblast differentiation, PPARG and CITED1, displayed increasing expression over time in EET (D12–D25) but increased to a much smaller degree in TVs (CITED1 only from D15–D18, Figure 6). The steroidogenic enzymes CYP2C18 and HSD17B2 had their highest expression in EET at D21, but exhibited variable expression in both ED and TV+ED with rather low levels in TVs. Interestingly, VIM and SNAI2 were upregulated at D18 and D21 in TV+ED [D18(15), D21(12)] compared to TVs [D18(15), D18(12), D21(12)], confirming the ED origin of epithelial–mesenchymal transition (EMT) (Figure 6). Upregulation in the regulatory genes TWIST1 and ZEB2 between D15 and D18 in EET and ED and in D18(D15) TV+ED—along with a similar pattern in extra-embryonic mesoderm genes such as BMP4, CD44, HAND1, and MMP2—highlights a phenomenon of increasing expression from D12 to D15 in ED and EET with no equivalence in TV or TV+ED. Regarding epithelial markers, the expression of genes associated with microvilli, such as EZR and RDX, declined over time in TVs that were derived from early stages [D15(12), D18(12), D21(12)], reflecting a potential loss of epithelial morphology during their elongation. Similarly, the expression of integrin genes such as ITGB1 and ITGB2 declined in D12-derived TVs at D15 and D18, suggesting reduced cell adhesion. ECM genes (*COL1A1*, *COL1A2*, *COL6A1*, *COL12A1*, and *ECM1*) were found to be upregulated in EET and ED, consistent with the extensive matrix remodeling that is linked to EMT; instead, expression levels in TVs were either relatively steady or exhibited a sharp decrease (SPP1, TGFBI, THBS2, and TNC, for instance; see TGFBI in Figure 6). The expression of cell junction molecules such as CADM1, CTNNB1, DES, and FLOT1 increased over time in EET, consistent with the increasing amount of cell contact, but this pattern was less pronounced in TVs (FLOT1, Figure 6). Certain genes controlling cell shape and polarity, such as CDC42EP3, CLASP1, MYLK2, and RHOA, were expressed earlier in development [D12 and D15 EET and TV derived from early stages: D15(12) and D18(12); see CLASP1 in Figure 6] and sometimes at higher levels in TV+ED (CLASP2), while others (CYPFIP2) were upregulated later on (D15–D25 EET; D12-derived TVs at D15, D18, and D21). Cell cycle regulators such as AURKB, CCND1, and CLASP1 were highly expressed in D15 trophoblasts and EDs, as well as in D12-derived TVs at D15 and D18, likely as a result of the rapid proliferation occurring in these types of tissues at these stages. Certain transporters linked to nutrient uptake, such as SLC2A4, SLC5A1, SLC5A11, SLC7A1, SLC7A3, and SLC27A1, displayed differential expression patterns over time in EET and/or TVs, while others demonstrated consistent expression in TVs (SLC2A3, SLC5A1, SLC7A1, SLC7A6, and SLC38A7). We found a high degree of variability in the expression of metabolic genes linked to fatty acids between ED, EET, TVs, and TV+ED, likely reflecting differences in the metabolic kinetics of various embryonic or extra-embryonic cell types. Among these, FOXO4 had a much clearer increase over time in ED and EET compared with TVs and TV+ED (Figure 6). Taken together, these data provide insights into the expression kinetics of key developmental genes, which in turn reflect the dynamics of various gene regulatory networks underlying the bovine elongation process.

## Discussion

Overall, the molecular signature of TVs follows a similar developmental trajectory as intact EET from day 12 to day 18. However, within each stage, distinct differences in gene expression are apparent between TV and intact EET samples. These differences shed light on some of the molecular cues that might be missing in TVs and could potentially provide answers to longstanding questions about the influence of extra-embryonic signaling (Gardner and Johnson, 1972). Moreover, these data can also be used to investigate the compensatory functions that allow TVs to cope with a uterine milieu in which they are considerably undersized (normal tissues develop from larger sizes). A comparison of these short EETs with similar short phenotypes reported from the gut epithelium could be an interesting way to study the putative post-repair pathways at work in TVs and other elongating epithelia.

### EET *versus* ED: dependent on each other or not?

As observed in this study, TVs only elongate once the embryo has engaged in the elongation process; thus, D8-derived TVs from the *in vitro* origin did not elongate, whereas TVs derived from ovoid and tubular *in vivo*-developed conceptuses at D12 and D15 after insemination did. Through the process of cutting EET samples into pieces, incubating them *in vitro* for 2 h in a B2 medium, transferring them into a new receptive uterus, and leaving them up to D15, D18, or D21, we were able to observe successful elongation, although not to the same extent as control conceptuses.

The earliest stage examined in this study was that of the spherical blastocyst, which was developed *in vitro*, deprived of its ED at D8 (ICM), transferred into a bovine uterus, and recovered at D12, D15, D18, or D21. No elongation was found to occur, except for a few cases that were recovered at D12 (Figure 1A). The lack of elongation is almost certainly the result of missing cues, which might originate from the extra-embryonic endoderm that lies under the trophoblast layer (ExEndod D9) (Betteridge et al., 1980; Flechon et al., 1986) or from the inner cell mass (ICM), as was reported at D7 in *in vitro* bovine blastocysts (Nagai et al., 2009) or at 3.5 dpc in murine blastocysts (Gardner and Johnson, 1972). An earlier study on D8-hatched bovine blastocysts (produced *in vivo* and maintained in SOFaa for 6 h before uterine transfer) did report successful splitting and proper elongation compared to control conceptuses (Velasquez et al., 2017); the difference in that case might have been the presence of an early endoderm layer (under the ICM) that extended throughout the whole blastocyst cavity so that the two halves, with or without a disc, produced similar recovery rates and mean sizes. Interestingly, in our study, we observed similar patterns of gene expression between the two sets of samples—D12-derived TVs at D18 and split D8 *in vivo* blastocysts that developed up to D17—with respect to the expression of the gene *FN1*, which is a usual mark for endoderm organization and differentiation (Artus et al., 2020).

Here, small pieces of D12 or D15 EET with no disc were found to elongate *in vivo*. However, we also observed that embryonic discs with only a short piece of EET (at the time of their transfer into the uterus) differentiated almost or completely normally by D15, 18, or 21. This supports previous research using *in vitro* mouse or human models in which gastrulation was accomplished with no EET at all, through the use of embryonic stem cells grown on adhesive micropatterns (Warmflash et al., 2014). Consistent with these results, in the present study, the size and shape of EDs from TVs were similar to those of EET controls (Figure 1B). Despite this, only few embryonic genes had similar expression patterns in both TV+ED and ED (EED, HHEX, and NODAL early patterns, LEFTY2, MYH1, and TBXT at D18), with a majority displaying lower or higher expression (Supplementary Figure S3).

### TVs *versus* EET: is elongation similar?

#### Elongation rate

In this study, TVs did elongate, but not to the same extent as EET (Figure 2A). At D15, TV and EET sizes were similar (36 vs. 38 mm), but at D18, D12- and D15-derived TVs were smaller than the EET of a similar stage (104 vs. 205 mm). With respect to their starting size, TVs appeared to elongate at a higher rate between D12 and D18 (60 mm added per mm) but at a lower rate from D15 to D18 (40 mm added per mm). Thus, D15-derived TVs at D18 elongated fairly like D12-derived TVs (11 mm per day and 9 mm per day), which makes sense given that the initial EET pieces were of similar sizes: 3 mm for the TVs generated at D15 *versus* 2.5 mm for those created at D12.

Thus, the process of sectioning D15 EET into pieces appears to have the overall effect of pushing TV development backwards to D12, considering their size and daily elongation rates. However, the uterus into which they are transferred is prepared for D15 EET development (Forde et al., 2014) so that short D15-derived TVs experience asynchrony. This situation resembles that of a younger embryo developing in an older uterus, as occurring in sub-fertile heifers with a less-receptive uterine milieu (Moraes et al., 2018). To investigate this hypothesis, we analyzed the expression of some of the genes of interest from that study in order to characterize any expression similarities or differences between D15-derived TVs and younger/shorter embryos (Supplementary Figure S3). Therefore, we found similar patterns of expression between these TVs and D12 and D15 EET with respect to the gene *DMRT1*, and between these TVs and D15 or D18 EET for the gene encoding FKBP9. The gene *GPRC5B* was underexpressed in TVs compared to D18 EET, while HOXA10 was found to be overexpressed in TVs compared to D25 EET. The one constant pattern in all of this is that D18 (D15-derived) TVs bear little resemblance to 1) D12 EET, although both shared the same initial size at the time of transfer; 2) D15 EET, even though TVs were sectioned at that stage, or 3) D18 EET, despite being of the same chronological age. An alternative explanation could be that a piece of EET that is shorter than usual has reduced signaling capacities and elicits different answers from the uterus, as described in the work of (Sanchez et al., 2019), and, thus, must adapt to compensate for physiodevelopmental asynchronies or mismatches in pairing.

In this case, the lack of an embryonic disc does not appear to be a missing cue. FGF4 and BMP4 signaling from the embryo to the trophoblast, as reported from early ovoid embryos in pigs (Valdez Magana et al., 2014), seems to be restricted to the EET that is neighboring the disc. In our study, D15-derived TVs did not originate solely from the EET that borders the ED but originated from the whole conceptus, which by D15 is tubular and 38 mm long so that 8 to 10 TVs of approximately 3 mm each can be produced.

#### Cell layers in TVs compared to EET

Depending on the stage of the conceptus that is cut into pieces to generate TVs, extra-embryonic endoderm (ExEn) or extra-embryonic mesoderm (ExM) may be formed by D12 or D14 (Betteridge et al., 1980; Maddox-Hyttel et al., 2003; Degrelle et al., 2005; van Leeuwen et al., 2015). Accordingly, D15 (D12-derived) TVs were found to express FN1 or LAMB1 similar to D12 EET and ED, indicating the presence of ExEn (Supplementary Figure S3). Likewise, D18 (D15-derived) TVs expressed CD44, MMP2, and SPARC similar to D15 EET, supporting the presence of an ExM layer. The trophoblast is also present, with most of those genes expressed in the same manner in TVs as in EET, although with more variability in their expression levels. Given these patterns, does the trophoblast develop normally in TVs? Or is there instead a lingering impact of the repair process that occurred a few days prior to seal the sectioned pieces of EET? As part of our transfer protocol, we evaluated the quality of sealing prior to TV transfer *in utero*, but it may be that the cumulative effects of repair and proliferation disturb normal elongation dynamics.

#### Repair and epithelial properties

Following tissue injury, the process of repair involves proliferation and patterning, and thus, the reactivation of embryonic programs (Goldman and Poss, 2020). For example, the sectioning of gut tissues was found to result in the formation of balls of wound-associated epithelial (WAE) cells, which transiently blocked enterocyte differentiation to create an epithelial barrier over the wound (Miyoshi and Chang, 2017). The TVs used in our experiment are morphologically similar to those balls and demonstrate a number of differences in the expression of repair genes compared to EET (Supplementary Figure S3), including slight upregulation (D12-derived D18 TV: ANGPT2 and ANXA1/A5; D12-derived D15: HYAL2; D15-derived D18 TV: CLND4), notable downregulation (D12-and D15-derived D18: DCN, Figure 6), or considerable upregulation (D12-derived D18 TV: DKK1). Additional research is needed on these repair genes in the hours following EET sectioning in order to compare the immediate and/or longstanding impacts, especially given the variety of recovery times used in the literature [e.g., 2 h (our protocol), 6 h (Velasquez et al., 2017), or 24 h (Heyman et al., 1984; Flechon et al., 1986)].

Moreover, both TVs and EET share a few similarities with gut epithelia: they elongate, absorb nutrients, and differentiate in response to morphogen gradients (BMP and TGFB; Beumer and Clevers, 2016; Lemke and Nelson, 2021; Berkova et al., 2023). Interestingly, these tissues also share genes (Supplementary Figure S3, Supplementary Table S3) linked to regeneration, such as CD44, CTNNB1, DKK1, HES1, and WNT5A (Beumer and Clevers, 2016; Horiguchi et al., 2017), or to stem cell niche potential, such as ASCL2, FOXO3, IHH, PDGFRA, WNT5A, DKK1, and PPARG (Santos et al., 2018), all of which demonstrated altered expression in TVs compared to EET. As the small intestine requires an enormous surface area for the absorption of nutrients, its process of elongation involves changes in cell size and shape, including microvilli. The signaling pathways involved in this lengthening employ WNT5A, FGF, HEDGEHOG, and NOTCH (Walton et al., 2016), which were all highlighted as genes of interest in the present study. Finally, previous research in mice revealed a connection between the length of the intestine and alterations in FGFR1 and FGFR2, which is interesting given that all TVs in this study underexpressed FGFR1 (Figure 6).

#### Elongation in TVs

In TVs that elongated, was the process similar to that of control tissues? Yes and no. For example (Supplementary Table S3), both EET and TVs similarly underexpressed PAG11 or AKR1B1 between D12 and D18 and B4GALT1 and PAG2 between D15 and D18, respectively. However, TVs overexpressed FABP5, GATA3, PAG2, and TKDP5 from D15 to D18, as observed in earlier-stage EET (D12 to D18). If we compare our results to those of Scatolin et al. (2023), it seems that TVs from D12–18 exhibited a mix of genes normally expressed at D12–D14 (HB1 and TB2–TB1) and D16–D18 (TB6). A similar pattern was evident for TVs at D15–18 as well, but to a lesser extent (HB1 and TB2–TB1: 54 genes instead of 106 in TV12–18; TB6: 16 genes instead of 36 in TV12–18). Genes related to lipid metabolism, nutrient uptake, or steroidogenic enzymes appeared to be largely expressed in the same manner in TVs as in EET, though not always at the same level, supporting the existence of at least a partial elongation process at work in TVs.

In summary, the distinct transcriptomic profiles and molecular signatures of TVs underscore the complexity of the regulatory networks that direct elongation and morphogenesis in the absence of embryonic inputs. Further investigation into the patterns of gene and pathway enrichment in TVs across different stages will provide insights into the mechanisms that enable extra-embryonic elongation in the absence of coordinated inputs from the embryonic disc and/or the uterine milieu (including extra-cellular vesicles or miRNA; Imakawa et al., 2022). In the future, the use of single-cell RNAseq would ensure that all DEGs are accounted for (Forde et al., 2014; Jia et al., 2023) and extend the repertoire of known gene expression changes. Such data could be combined with other models of tissue elongation in order to highlight common themes, including patterning, spatiotemporal cell fate, or physical forces that enable proper morphogenesis (Horne-Badovinac, 2014; Weng et al., 2022; Adler et al., 2023). Taken together, our results provide useful information for the future analyses of the factors that coordinate the conserved morphogenetic processes at work during bovine conceptus development, processes that one may evaluate in the coming years through novel cell culture modes (Walsh et al., 2023), cocultures (Wei et al., 2023), or *in silico* predictive models (Butt et al., 2023).

## Data Availability

The datasets presented in this study can be found in online repositories. The names of the repository/repositories and accession number(s) can be found at: https://www.ncbi.nlm.nih.gov/geo/, GSE146768.

## Supplementary material

SUPPLEMENTARY FIGURE S1 Heatmap of DEGs between all bovine TVs and embryo control samples, including EDs and EETs. DEGs (*n*=553) were identified by one-way ANOVA (*p*<0.05) from the full dataset of 120 samples across four stages (D8, D12, D15, and D18), using two replicates (-1 and -2). Hierarchical clustering was performed using PermutMatrix. Each row represents a gene, and each column represents a sample or condition, with the color scale indicating the expression level from low (blue) to high (red).

SUPPLEMENTARY FIGURE S2 Differential gene expression and associated functional pathway enrichment in bovine trophoblastic vesicles and extra-embryonic tissues. Enrichment analysis was performed to identify which pathways from the Kyoto Encyclopedia of Genes and Genomes (KEGG) were associated with the DEGs that were found to be up- or downregulated in bovine TVs compared to intact EETs. DEGs were identified from comparisons of different gestational stages (D12 vs. D15, D12 vs. D18, and D15 vs. D18). The DEG numbers, between parentheses, are given in Supplementary Table S3B. Significantly enriched KEGG pathways (adj. p <0.05) are shown separately for up- and downregulated DEGs in TV and EET. In the right panel, the p-value and gene ratio for specific GO categories are given. Underlined are those on which we focused.

SUPPLEMENTARY FIGURE S3 OpenArray data analyses. Each boxplot represents the distribution of log2(RQ) values for one of 224 genes, categorized into 24 biological pathways (Supplementary Table S4), across four distinct types of tissues: ED, EETs, TVs, and TVs containing the ED (TV+ED=CTRL TV). Each boxplot depicts the median, interquartile range, and potential outliers of the gene expression values across five gestational days (D12, D15, D18, D21, and D25).

## Abbreviations

AI, artificial insemination; BCA, between-class analysis of variance; dpc, days post-coitum; ED, embryonic disc; EET, extra-embryonic tissue; GO, Gene Ontology; IVP, *in vitro*-produced; TV, trophoblastic vesicle.

## References

[B1] Adler M. Moriel N. Goeva A. Avraham-Davidi I. Mages S. Adams T. S. (2023). Emergence of division of labor in tissues through cell interactions and spatial cues. Cell Rep. 42, 112412. 10.1016/j.celrep.2023.112412 37086403 PMC10242439

[B2] Akizawa H. Saito S. Kohri N. Furukawa E. Hayashi Y. Bai H. (2021). Deciphering two rounds of cell lineage segregations during bovine preimplantation development. FASEB J. 35, e21904. 10.1096/fj.202002762RR 34569650

[B3] Artus J. Hue I. Acloque H. (2020). Preimplantation development in ungulates: a 'menage a quatre' scenario. Reproduction 159, R151–R172. 10.1530/REP-19-0348 31751293

[B4] Bazer F. W. Wang X. Johnson G. A. Wu G. (2015). Select nutrients and their effects on conceptus development in mammals. Anim. Nutr. 1, 85–95. 10.1016/j.aninu.2015.07.005 29767122 PMC5945975

[B5] Berkova L. Fazilaty H. Yang Q. Kubovciak J. Stastna M. Hrckulak D. (2023). Terminal differentiation of villus tip enterocytes is governed by distinct Tgfβ superfamily members. EMBO Rep. 24, e56454. 10.15252/embr.202256454 37493498 PMC10481656

[B6] Bernardo A. S. Jouneau A. Marks H. Kensche P. Kobolak J. Freude K. (2018). Mammalian embryo comparison identifies novel pluripotency genes associated with the naive or primed state. Biol. Open 7, bio033282. 10.1242/bio.033282 30026265 PMC6124576

[B7] Betteridge K. J. Eaglesome M. D. Randall G. C. Mitchell D. (1980). Collection, description and transfer of embryos from cattle 10--16 days after oestrus. J. Reprod. Fertil. 59, 205–216. 10.1530/jrf.0.0590205 7401037

[B8] Beumer J. Clevers H. (2016). Regulation and plasticity of intestinal stem cells during homeostasis and regeneration. Development 143, 3639–3649. 10.1242/dev.133132 27802133

[B9] Blomberg L. Hashizume K. Viebahn C. (2008). Blastocyst elongation, trophoblastic differentiation, and embryonic pattern formation. Reproduction 135, 181–195. 10.1530/REP-07-0355 18239048

[B10] Brandao D. O. Maddox-Hyttel P. Lovendahl P. Rumpf R. Stringfellow D. Callesen H. (2004). Post hatching development: a novel system for extended *in vitro* culture of bovine embryos. Biol. Reprod. 71, 2048–2055. 10.1095/biolreprod.103.025916 15329327

[B11] Brooks K. E. Burns G. W. Spencer T. E. (2015). Peroxisome proliferator activator receptor gamma (PPARG) regulates conceptus elongation in sheep. Biol. Reprod. 92, 42. 10.1095/biolreprod.114.123877 25519185

[B12] Butt Z. Tinning H. O'connell M. J. Fenn J. Alberio R. Forde N. (2023). Understanding conceptus-maternal interactions: what tools do we need to develop? Reprod. Fertil. Dev. 36, 81–92. 10.1071/RD23181 38064186

[B13] Caraux G. Pinloche S. (2005). PermutMatrix: a graphical environment to arrange gene expression profiles in optimal linear order. Bioinformatics 21, 1280–1281. 10.1093/bioinformatics/bti141 15546938

[B14] Clemente M. De La Fuente J. Fair T. Al Naib A. Gutierrez-Adan A. Roche J. F. (2009). Progesterone and conceptus elongation in cattle: a direct effect on the embryo or an indirect effect via the endometrium? Reproduction 138, 507–517. 10.1530/REP-09-0152 19556439

[B15] Comizzoli P. Marquant-Le Guienne B. Heyman Y. Renard J. P. (2000). Onset of the first S-phase is determined by a paternal effect during the G1-phase in bovine zygotes. Biol. Reprod. 62, 1677–1684. 10.1095/biolreprod62.6.1677 10819771

[B16] Degrelle S. A. Campion E. Cabau C. Piumi F. Reinaud P. Richard C. (2005). Molecular evidence for a critical period in mural trophoblast development in bovine blastocysts. Dev. Biol. 288, 448–460. 10.1016/j.ydbio.2005.09.043 16289134

[B17] Degrelle S. A. Jaffrezic F. Campion E. Le Cao K. A. Le Bourhis D. Richard C. (2012). Uncoupled embryonic and extra-embryonic tissues compromise blastocyst development after somatic cell nuclear transfer. PLoS One 7, e38309. 10.1371/journal.pone.0038309 22701625 PMC3368877

[B18] Degrelle S. A. Le Cao K. A. Heyman Y. Everts R. E. Campion E. Richard C. (2011a). A small set of extra-embryonic genes defines a new landmark for bovine embryo staging. Reproduction 141, 79–89. 10.1530/REP-10-0174 20926692

[B70] Degrelle S. A. Murthi P. Evain-Brion D. Fournier T. Hue I. (2011b). Expression and localization of DLX3, PPARG and SP1 in bovine trophoblast during binucleated cell differentiation. Placenta 32 (11), 917–920. 10.1016/j.placenta.2011.08.014 21937107

[B19] Flechon J. E. Guillomot M. Charlier M. Flechon B. Martal J. (1986). Experimental studies on the elongation of the Ewe blastocyst. Reprod. Nutr. Dev. 26, 1017–1024. 10.1051/rnd:19860609 3775097

[B20] Forde N. Simintiras C. A. Sturmey R. Mamo S. Kelly A. K. Spencer T. E. (2014). Amino acids in the uterine luminal fluid reflects the temporal changes in transporter expression in the endometrium and conceptus during early pregnancy in cattle. PLoS One 9, e100010. 10.1371/journal.pone.0100010 24960174 PMC4069017

[B21] Gardner R. L. Johnson M. H. (1972). An investigation of inner cell mass and trophoblast tissues following their isolation from the mouse blastocyst. J. Embryol. Exp. Morphol. 28, 279–312. 10.1242/dev.28.2.279 4672104

[B22] Goldman J. A. Poss K. D. (2020). Gene regulatory programmes of tissue regeneration. Nat. Rev. Genet. 21, 511–525. 10.1038/s41576-020-0239-7 32504079 PMC7448550

[B23] Gray C. A. Burghardt R. C. Johnson G. A. Bazer F. W. Spencer T. E. (2002). Evidence that absence of endometrial gland secretions in uterine gland knockout ewes compromises conceptus survival and elongation. Reproduction 124, 289–300. 10.1530/rep.0.1240289 12141942

[B24] Greenstein J. S. Murray R. W. Foley R. C. (1958). Observations on the morphogenesis and histochemistry of the bovine preattachment placenta between 16 and 33 days of gestation. Anat. Rec. 132, 321–341. 10.1002/ar.1091320308 13637407

[B25] Heyman Y. Camous S. Fevre J. Meziou W. Martal J. (1984). Maintenance of the corpus luteum after uterine transfer of trophoblastic vesicles to cyclic cows and ewes. J. Reprod. Fertil. 70, 533–540. 10.1530/jrf.0.0700533 6699815

[B26] Horiguchi H. Endo M. Kawane K. Kadomatsu T. Terada K. Morinaga J. (2017). ANGPTL2 expression in the intestinal stem cell niche controls epithelial regeneration and homeostasis. EMBO J. 36, 409–424. 10.15252/embj.201695690 28043948 PMC5694950

[B27] Horne-Badovinac S. (2014). The Drosophila egg chamber-a new spin on how tissues elongate. Integr. Comp. Biol. 54, 667–676. 10.1093/icb/icu067 24920751 PMC4229889

[B28] Hue I. Dufort I. Vitorino Carvalho A. Laloe D. Peynot N. Degrelle S. A. (2018). Different pre-implantation phenotypes of bovine blastocysts produced *in vitro* . Reproduction 157, 163–178. 10.1530/REP-18-0439 30444718

[B29] Hue I. Evain-Brion D. Fournier T. Degrelle S. A. (2015). Primary bovine extra-embryonic cultured cells: a new resource for the study of *in vivo* peri-implanting phenotypes and mesoderm formation. PLoS One 10, e0127330. 10.1371/journal.pone.0127330 26070137 PMC4466545

[B30] Imakawa K. Matsuno Y. Fujiwara H. (2022). New roles for EVs, miRNA and lncRNA in bovine embryo implantation. Front. Vet. Sci. 9, 944370. 10.3389/fvets.2022.944370 35909679 PMC9334902

[B31] Jia G. X. Ma W. J. Wu Z. B. Li S. Zhang X. Q. He Z. (2023). Single-cell transcriptomic characterization of sheep conceptus elongation and implantation. Cell Rep. 42, 112860. 10.1016/j.celrep.2023.112860 37494181

[B32] Khan D. R. Dube D. Gall L. Peynot N. Ruffini S. Laffont L. (2012). Expression of pluripotency master regulators during two key developmental transitions: EGA and early lineage specification in the bovine embryo. PLoS One 7, e34110. 10.1371/journal.pone.0034110 22479535 PMC3315523

[B33] Lemke S. B. Nelson C. M. (2021). Dynamic changes in epithelial cell packing during tissue morphogenesis. Curr. Biol. 31, R1098–R1110. 10.1016/j.cub.2021.07.078 34582821 PMC8496771

[B71] Liu F. Rouault C. Guesnon M. Zhu W. Clément K. Degrelle S. (2020). Comparative study of PPARγ targets in human extravillous and villous cytotrophoblasts. PPAR Res. 2020, 1–18. 10.1155/2020/9210748 PMC7152979 32308672

[B34] Maddox-Hyttel P. Alexopoulos N. I. Vajta G. Lewis I. Rogers P. Cann L. (2003). Immunohistochemical and ultrastructural characterization of the initial post-hatching development of bovine embryos. Reproduction 125, 607–623. 10.1530/rep.0.1250607 12683931

[B35] Mamo S. Mehta J. P. Mcgettigan P. Fair T. Spencer T. E. Bazer F. W. (2011). RNA sequencing reveals novel gene clusters in bovine conceptuses associated with maternal recognition of pregnancy and implantation. Biol. Reprod. 85, 1143–1151. 10.1095/biolreprod.111.092643 21795669

[B36] Mamo S. Rizos D. Lonergan P. (2012). Transcriptomic changes in the bovine conceptus between the blastocyst stage and initiation of implantation. Anim. Reprod. Sci. 134, 56–63. 10.1016/j.anireprosci.2012.08.011 22944169

[B37] Mathew D. J. Peterson K. D. Senn L. K. Oliver M. A. Ealy A. D. (2022). Ruminant conceptus-maternal interactions: interferon-tau and beyond. J. Anim. Sci. 100, skac123. 10.1093/jas/skac123 35772752 PMC9246669

[B38] Menck M. C. Guyader-Joly C. Peynot N. Le Bourhis D. Lobo R. B. Renard J. P. (1997). Beneficial effects of Vero cells for developing IVF bovine eggs in two different coculture systems. Reprod. Nutr. Dev. 37, 141–150. 10.1051/rnd:19970202 9178355

[B39] Miyoshi J. Chang E. B. (2017). The gut microbiota and inflammatory bowel diseases. Transl. Res. 179, 38–48. 10.1016/j.trsl.2016.06.002 27371886 PMC5156589

[B40] Moraes J. G. N. Behura S. K. Geary T. W. Hansen P. J. Neibergs H. L. Spencer T. E. (2018). Uterine influences on conceptus development in fertility-classified animals. Proc. Natl. Acad. Sci. U. S. A. 115, E1749–E1758. 10.1073/pnas.1721191115 29432175 PMC5828633

[B41] Nagai K. Sata R. Takahashi H. Okano A. Kawashima C. Miyamoto A. (2009). Production of trophoblastic vesicles derived from Day 7 and 8 blastocysts of *in vitro* origin and the effect of intrauterine transfer on the interestrous intervals in Japanese black heifers. J. Reprod. Dev. 55, 454–459. 10.1262/jrd.20222 19420837

[B42] Peixoto P. M. Bromfield J. J. Ribeiro E. S. Santos J. E. P. Thatcher W. W. Bisinotto R. S. (2023). Transcriptome changes associated with elongation of bovine conceptuses II: differentially expressed transcripts in the endometrium on day 17 after insemination. J. Dairy Sci. 106, 9763–9777. 10.3168/jds.2023-23399 37641338

[B43] Perez-Gomez A. Gonzalez-Brusi L. Bermejo-Alvarez P. Ramos-Ibeas P. (2021). Lineage differentiation markers as a proxy for embryo viability in farm ungulates. Front. Vet. Sci. 8, 680539. 10.3389/fvets.2021.680539 34212020 PMC8239129

[B44] Pfeffer P. L. Pearton D. J. (2012). Trophoblast development. Reproduction 143, 231–246. 10.1530/REP-11-0374 22223687

[B45] Pfeffer P. L. Smith C. S. Maclean P. Berg D. K. (2017). Gene expression analysis of bovine embryonic disc, trophoblast and parietal hypoblast at the start of gastrulation. Zygote 25, 265–278. 10.1017/S0967199417000090 28534463

[B46] Pillai V. V. Siqueira L. G. Das M. Kei T. G. Tu L. N. Herren A. W. (2019). Physiological profile of undifferentiated bovine blastocyst-derived trophoblasts. Biol. Open 8, bio037937. 10.1242/bio.037937 30952696 PMC6550082

[B47] Pope W. F. (1988). Uterine asynchrony: a cause of embryonic loss. Biol. Reprod. 39, 999–1003. 10.1095/biolreprod39.5.999 3064819

[B48] Ramos-Ibeas P. Gonzalez-Brusi L. Used M. T. Cocero M. J. Marigorta P. Alberio R. (2022). *In vitro* culture of ovine embryos up to early gastrulating stages. Development 149, dev199743. 10.1242/dev.199743 35319748 PMC8977095

[B49] Ribeiro E. S. (2018). Symposium review: lipids as regulators of conceptus development: implications for metabolic regulation of reproduction in dairy cattle. J. Dairy Sci. 101, 3630–3641. 10.3168/jds.2017-13469 29174158

[B50] Ribeiro E. S. Greco L. F. Bisinotto R. S. Lima F. S. Thatcher W. W. Santos J. E. (2016). Biology of preimplantation conceptus at the onset of elongation in dairy cows. Biol. Reprod. 94, 97. 10.1095/biolreprod.115.134908 26935601

[B51] Richard C. Hue I. Gelin V. Neveux A. Campion E. Degrelle S. A. (2015). Transcervical collection of bovine embryos up to Day 21: an 8-year overview. Theriogenology 83, 1101–1109. 10.1016/j.theriogenology.2014.12.005 25662200

[B52] Sanchez J. M. Mathew D. J. Behura S. K. Passaro C. Charpigny G. Butler S. T. (2019). Bovine endometrium responds differentially to age-matched short and long conceptuses†. Biol. Reprod. 101, 26–39. 10.1093/biolre/ioz060 30977805 PMC6614577

[B53] Santos A. J. M. Lo Y. H. Mah A. T. Kuo C. J. (2018). The intestinal stem cell niche: homeostasis and adaptations. Trends Cell Biol. 28, 1062–1078. 10.1016/j.tcb.2018.08.001 30195922 PMC6338454

[B54] Scatolin G. N. Ming H. Wang Y. Zhu L. Castillo E. G. Bondioli K. (2023). Single-cell transcriptional landscapes of bovine peri-implantation development. bioRxiv. 10.1016/j.isci.2024.109605 PMC11022056 38633001

[B55] Spencer T. E. Kelleher A. M. Bartol F. F. (2019). Development and function of uterine glands in domestic animals. Annu. Rev. Anim. Biosci. 7, 125–147. 10.1146/annurev-animal-020518-115321 30183326

[B56] Stojkovic M. Zakhartchenko V. Brem G. Wolf E. (1997). Support for the development of bovine embryos *in vitro* by secretions of bovine trophoblastic vesicles derived *in vitro* . J. Reprod. Fertil. 111, 191–196. 10.1530/jrf.0.1110191 9462285

[B72] Taberlet P. Prud’Homme S. M. Campione E. Roy J. Miquel C. Shehzad W. (2012). Soil sampling and isolation of extracellular DNA from large amount of starting material suitable for metabarcoding studies. Mol. Ecol. 21 (8), 1816–1820. 10.1111/j.1365-294X.2011.05317.x 22300434

[B57] Ushizawa K. Herath C. B. Kaneyama K. Shiojima S. Hirasawa A. Takahashi T. (2004). cDNA microarray analysis of bovine embryo gene expression profiles during the pre-implantation period. Reprod. Biol. Endocrinol. 2, 77. 10.1186/1477-7827-2-77 15560851 PMC535809

[B58] Vajta G. Alexopoulos N. I. Callesen H. (2004). Rapid growth and elongation of bovine blastocysts *in vitro* in a three-dimensional gel system. Theriogenology 62, 1253–1263. 10.1016/j.theriogenology.2004.01.007 15325552

[B59] Valdez Magana G. Rodriguez A. Zhang H. Webb R. Alberio R. (2014). Paracrine effects of embryo-derived FGF4 and BMP4 during pig trophoblast elongation. Dev. Biol. 387, 15–27. 10.1016/j.ydbio.2014.01.008 24445281

[B60] Valour D. Degrelle S. A. Ponter A. A. Giraud-Delville C. Campion E. Guyader-Joly C. (2014). Energy and lipid metabolism gene expression of D18 embryos in dairy cows is related to dam physiological status. Physiol. Genomics 46, 39–56. 10.1152/physiolgenomics.00091.2013 24220328

[B61] Van Den Brink S. C. Van Oudenaarden A. (2021). 3D gastruloids: a novel frontier in stem cell-based *in vitro* modeling of mammalian gastrulation. Trends Cell Biol. 31, 747–759. 10.1016/j.tcb.2021.06.007 34304959

[B62] Van Leeuwen J. Berg D. K. Pfeffer P. L. (2015). Morphological and gene expression changes in cattle embryos from hatched blastocyst to early gastrulation stages after transfer of *in vitro* produced embryos. PLoS One 10, e0129787. 10.1371/journal.pone.0129787 26076128 PMC4468082

[B63] Velasquez A. E. Manriquez J. Castro F. O. Cox J. F. Rodriguez-Alvarez L. (2017). Embryo splitting affects the transcriptome during elongation stage of *in vitro*-produced bovine blastocysts. Theriogenology 87, 124–134. 10.1016/j.theriogenology.2016.08.014 27641677

[B64] Walsh S. C. Miles J. R. Broeckling C. D. Rempel L. A. Wright-Johnson E. C. Pannier A. K. (2023). Secreted metabolome of porcine blastocysts encapsulated within *in vitro* 3D alginate hydrogel culture systems undergoing morphological changes provides insights into specific mechanisms involved in the initiation of porcine conceptus elongation. Reprod. Fertil. Dev. 35, 375–394. 10.1071/RD22210 36780705

[B65] Walsh S. W. Williams E. J. Evans A. C. (2011). A review of the causes of poor fertility in high milk producing dairy cows. Anim. Reprod. Sci. 123, 127–138. 10.1016/j.anireprosci.2010.12.001 21255947 PMC7125520

[B66] Walton K. D. Freddo A. M. Wang S. Gumucio D. L. (2016). Generation of intestinal surface: an absorbing tale. Development 143, 2261–2272. 10.1242/dev.135400 27381224 PMC4958325

[B67] Warmflash A. Sorre B. Etoc F. Siggia E. D. Brivanlou A. H. (2014). A method to recapitulate early embryonic spatial patterning in human embryonic stem cells. Nat. Methods 11, 847–854. 10.1038/nmeth.3016 24973948 PMC4341966

[B68] Wei Y. Zhang E. Yu L. Ci B. Sakurai M. Guo L. (2023). Dissecting embryonic and extraembryonic lineage crosstalk with stem cell co-culture. Cell 186, 5859–5875. 10.1016/j.cell.2023.11.008 38052213 PMC10916932

[B69] Weng S. Huebner R. J. Wallingford J. B. (2022). Convergent extension requires adhesion-dependent biomechanical integration of cell crawling and junction contraction. Cell Rep. 39, 110666. 10.1016/j.celrep.2022.110666 35476988 PMC9119128

