# Supplementary figures and images for: Understanding bovine embryo elongation: a transcriptomic study of trophoblastic vesicles
Source: Front Physiol. 2024 Jan 29;15:1331098. doi: 10.3389/fphys.2024.1331098 (PMC10859461; doi:10.3389/fphys.2024.1331098)

# Figure S2

## KEGG\_10cat\_CTRL&VT-up&down

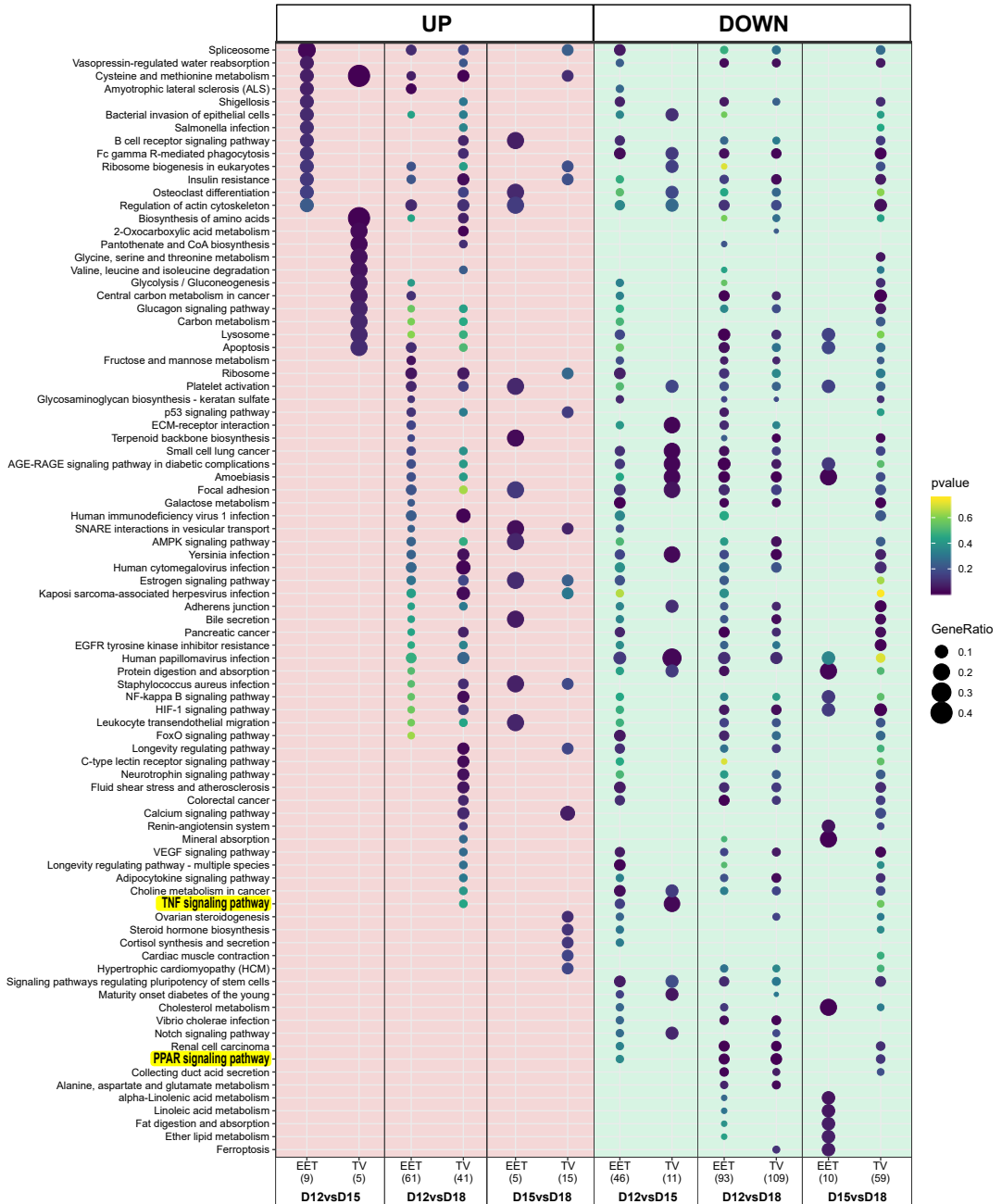

Supplement: Supplementary file 1 [file DataSheet2.PDF]

# Figure S1

## ALL SAMPLES

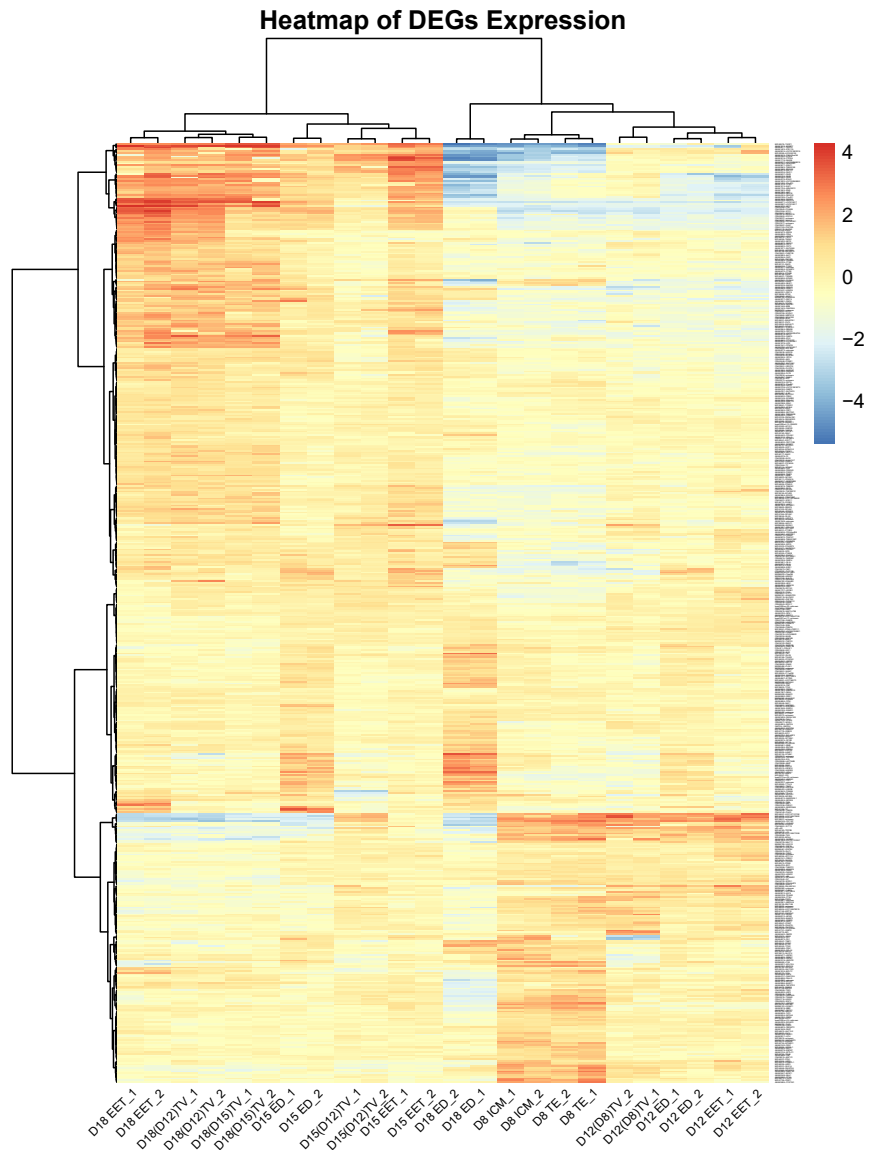

## Without (ICM, ED) SAMPLES

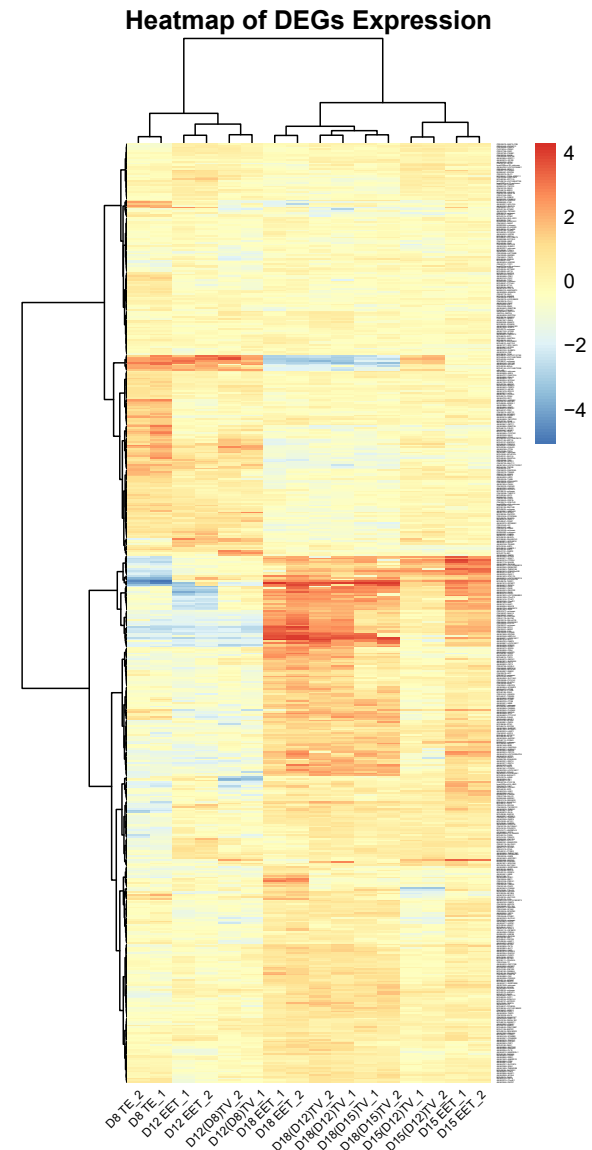

Supplement: Supplementary file 5 [file DataSheet1.PDF]
