# Supplementary material for: Understanding bovine embryo elongation: a transcriptomic study of trophoblastic vesicles
Source: Front Physiol. 2024 Jan 29;15:1331098. doi: 10.3389/fphys.2024.1331098 (PMC10859461; doi:10.3389/fphys.2024.1331098)

Biological Pathway = Emb Tissues

DNMT3A

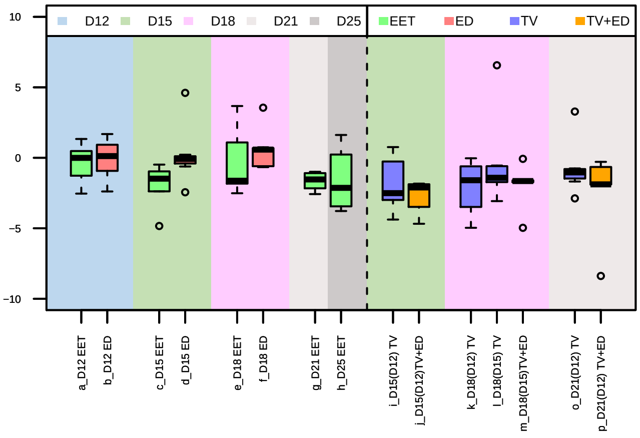

DNMT3B

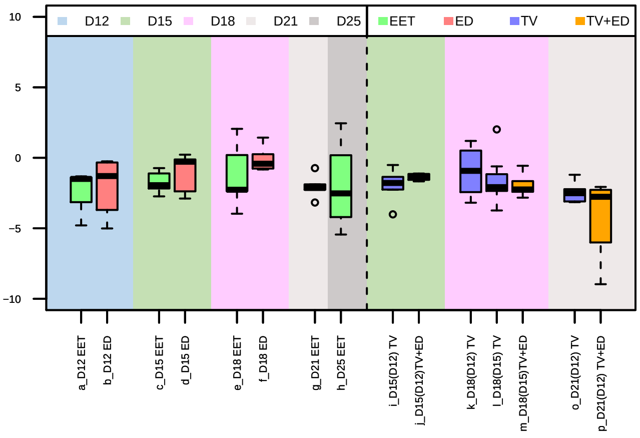

EED

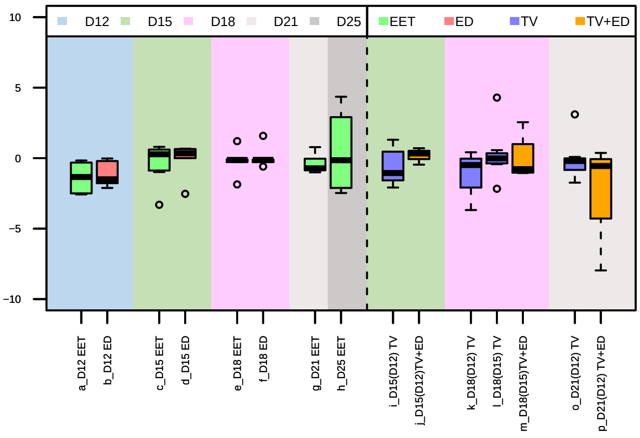

EOMES

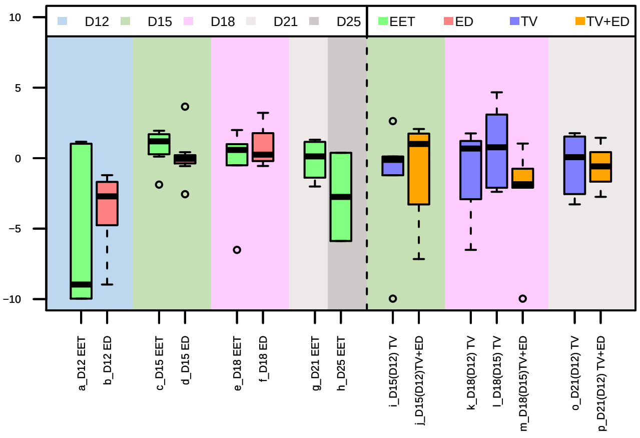

GSC

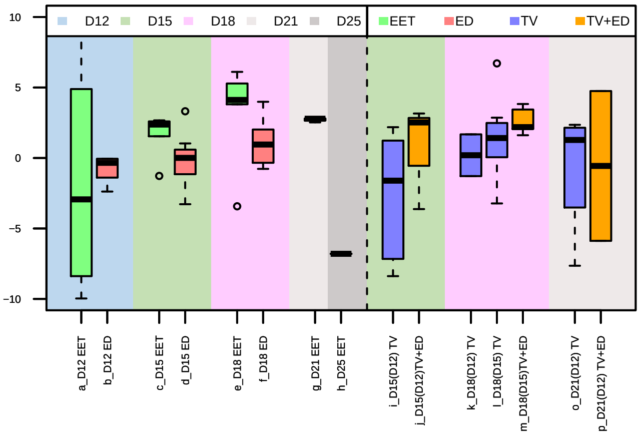

HHEX

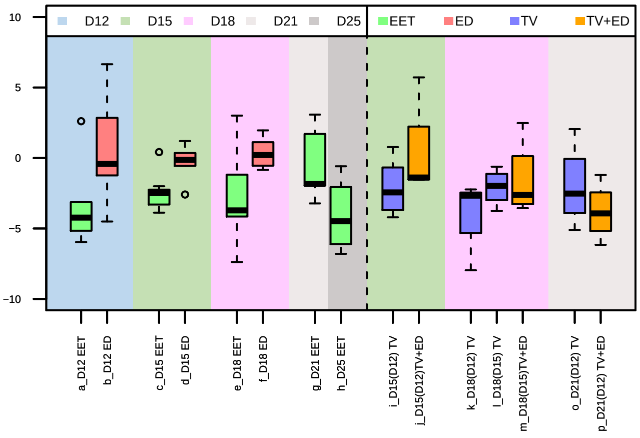

LEFTY2

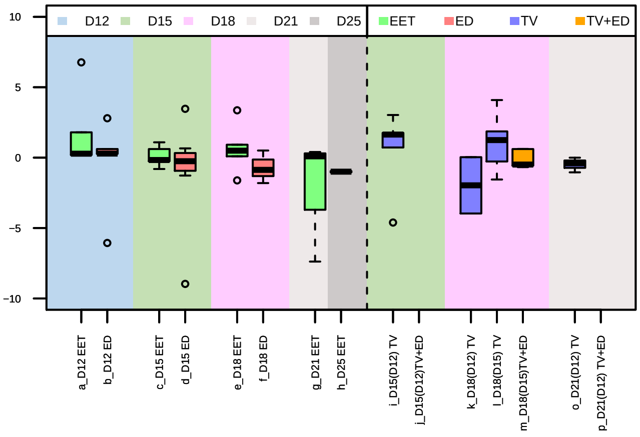

MYH1

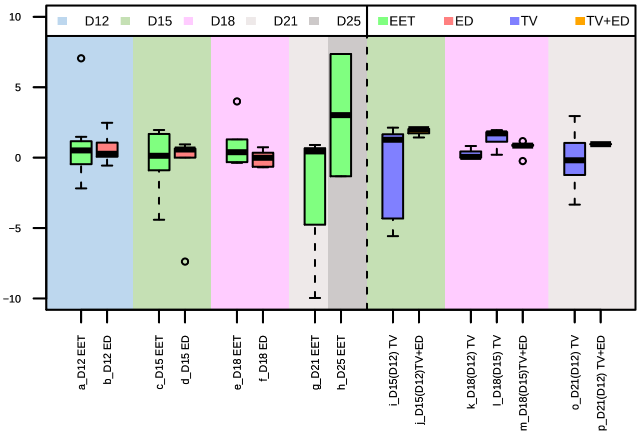

Biological Pathway = Emb Tissues

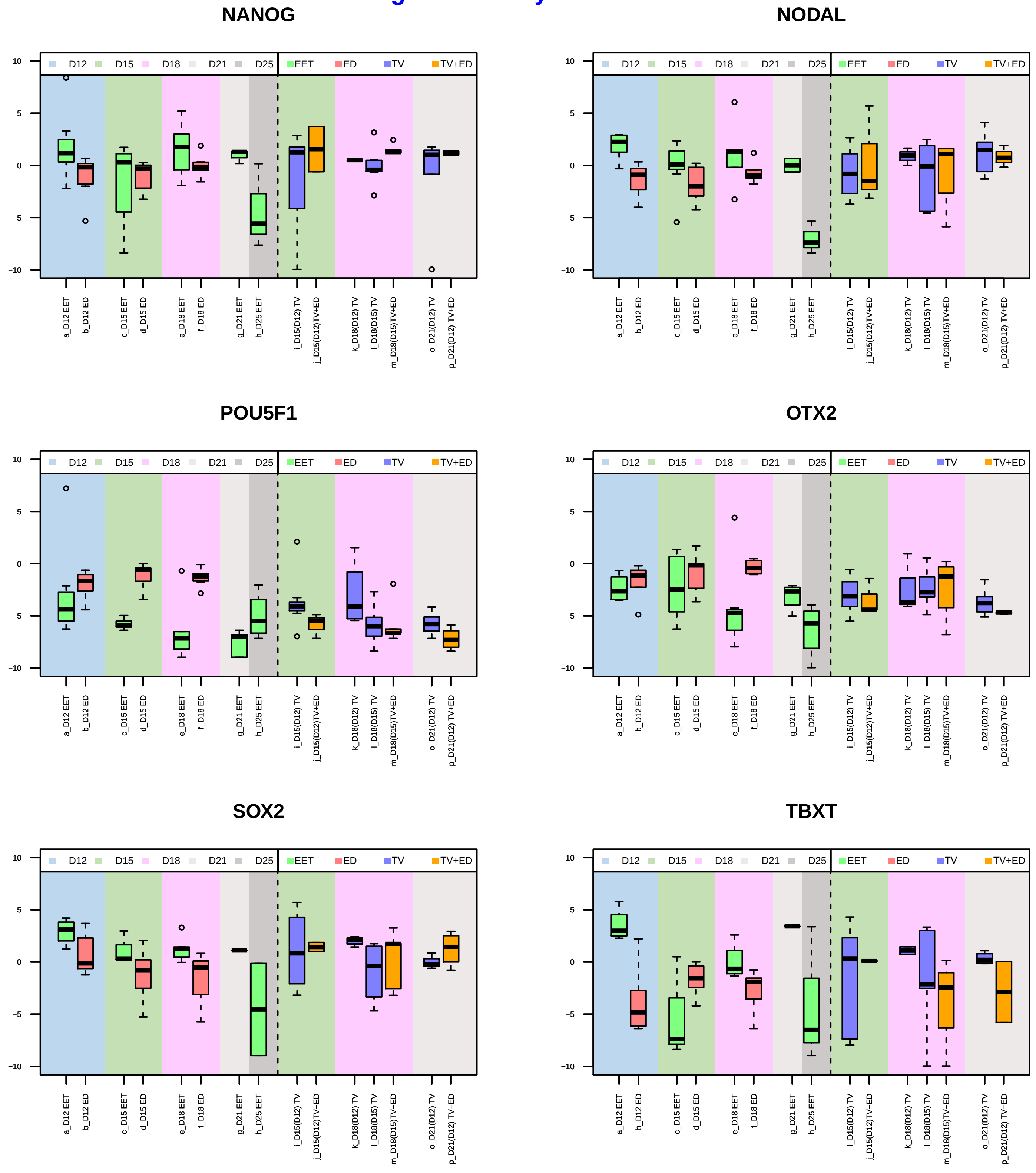

Biological Pathway = Trophoblast

ASCL2

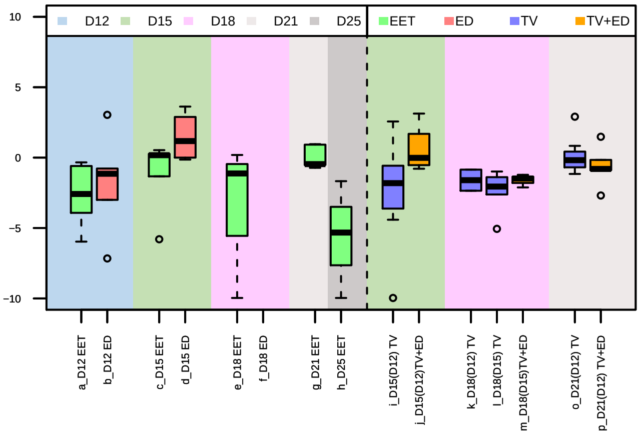

CD163

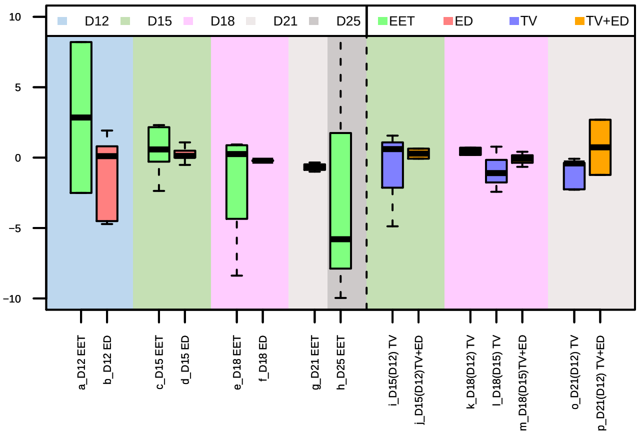

CDX2

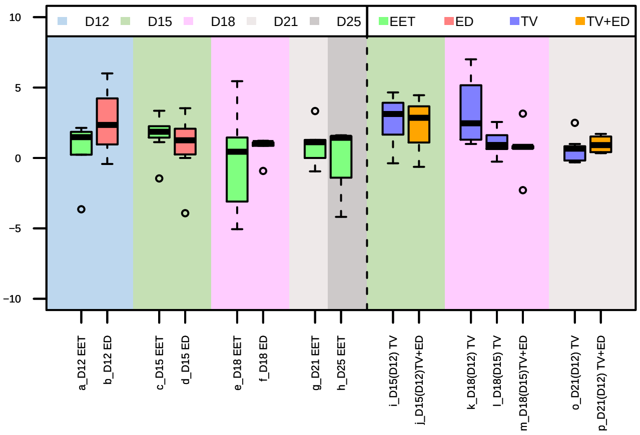

ETS2

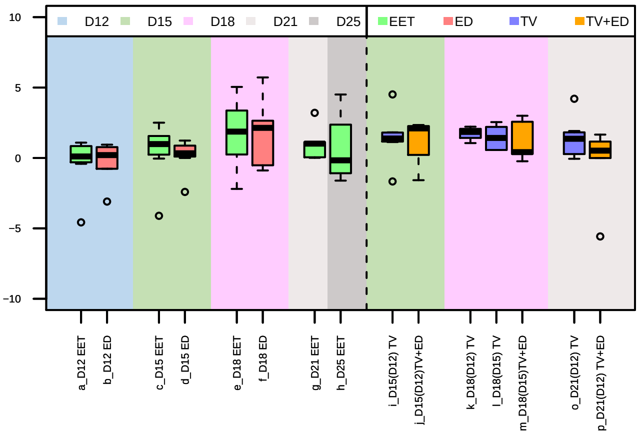

FGFR2

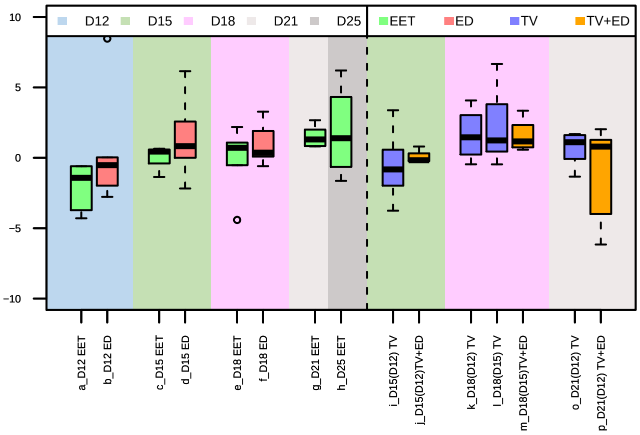

FURIN

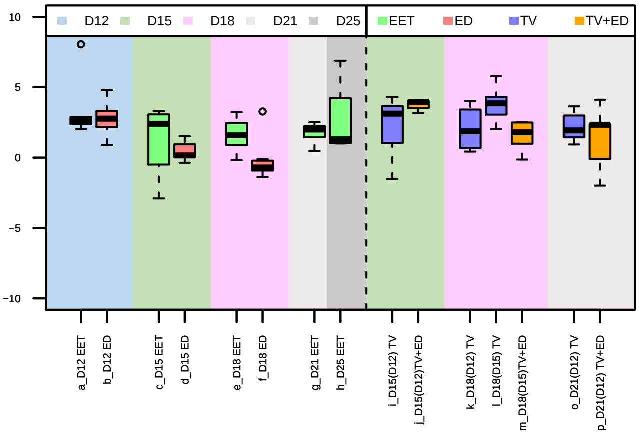

GATA3

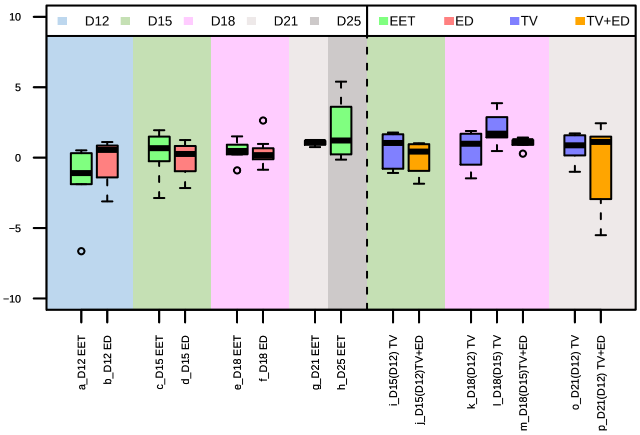

HOPX

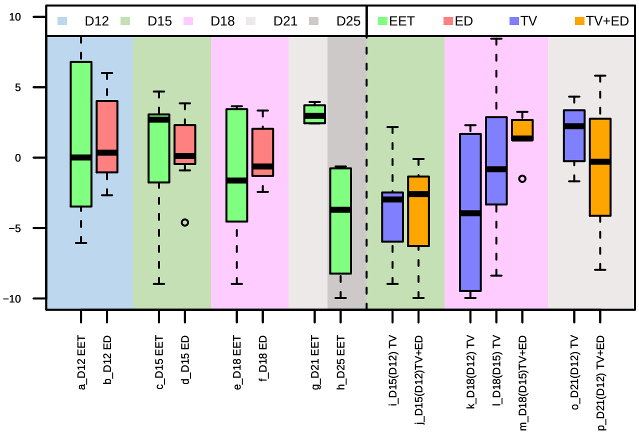

Biological Pathway = Trophoblast

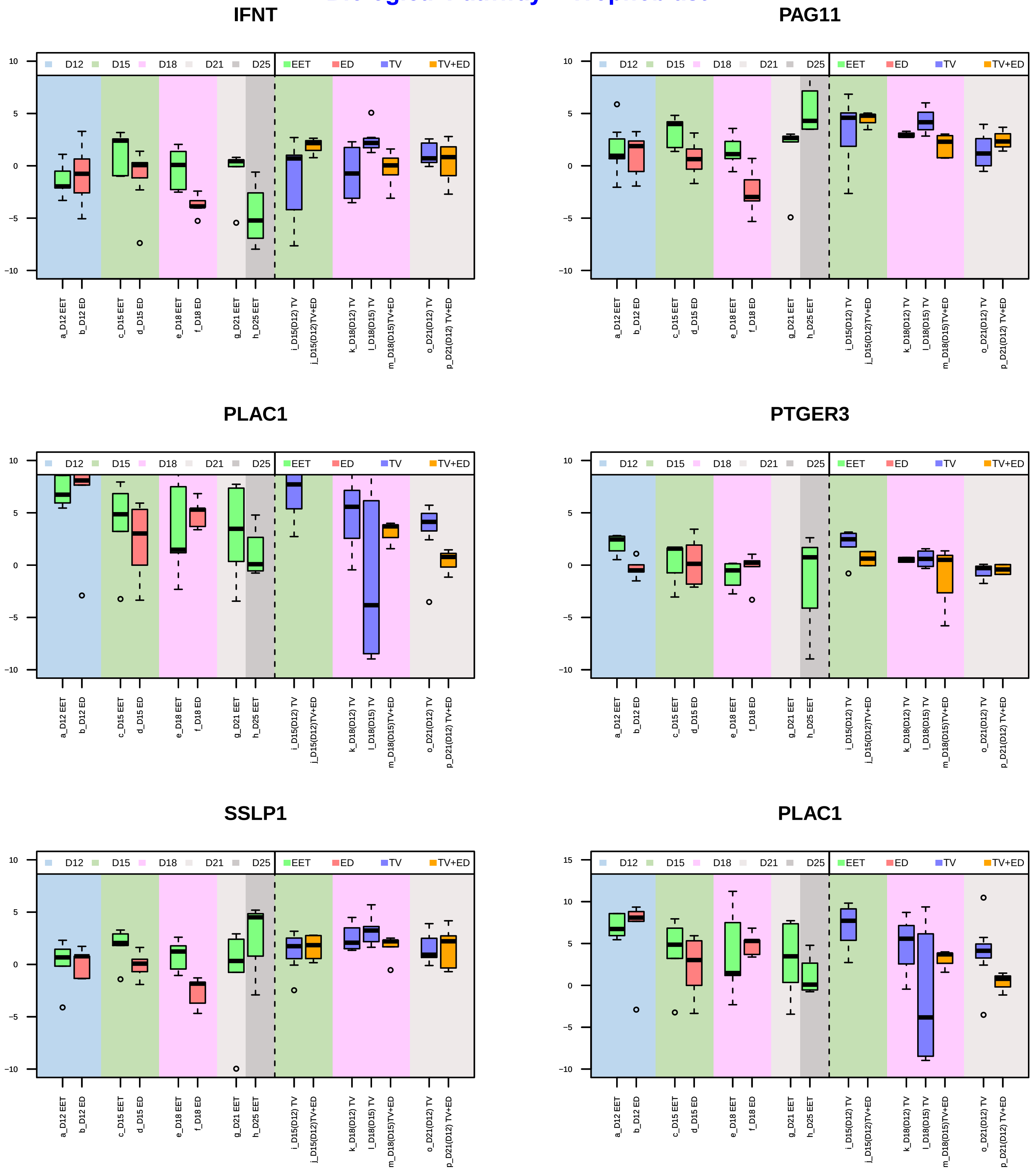

Biological Pathway = Endoderm

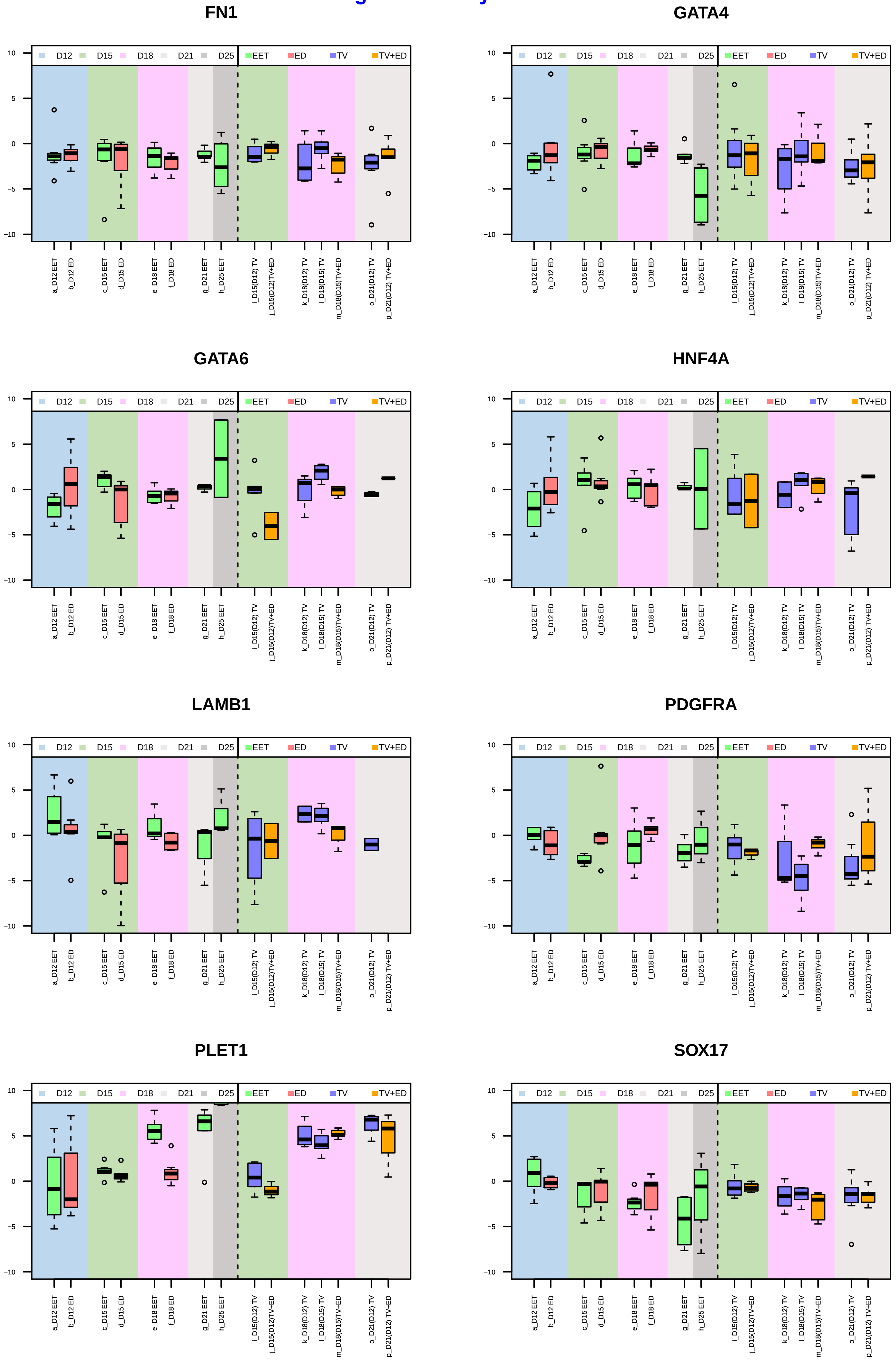

Biological Pathway = Endo + Meso

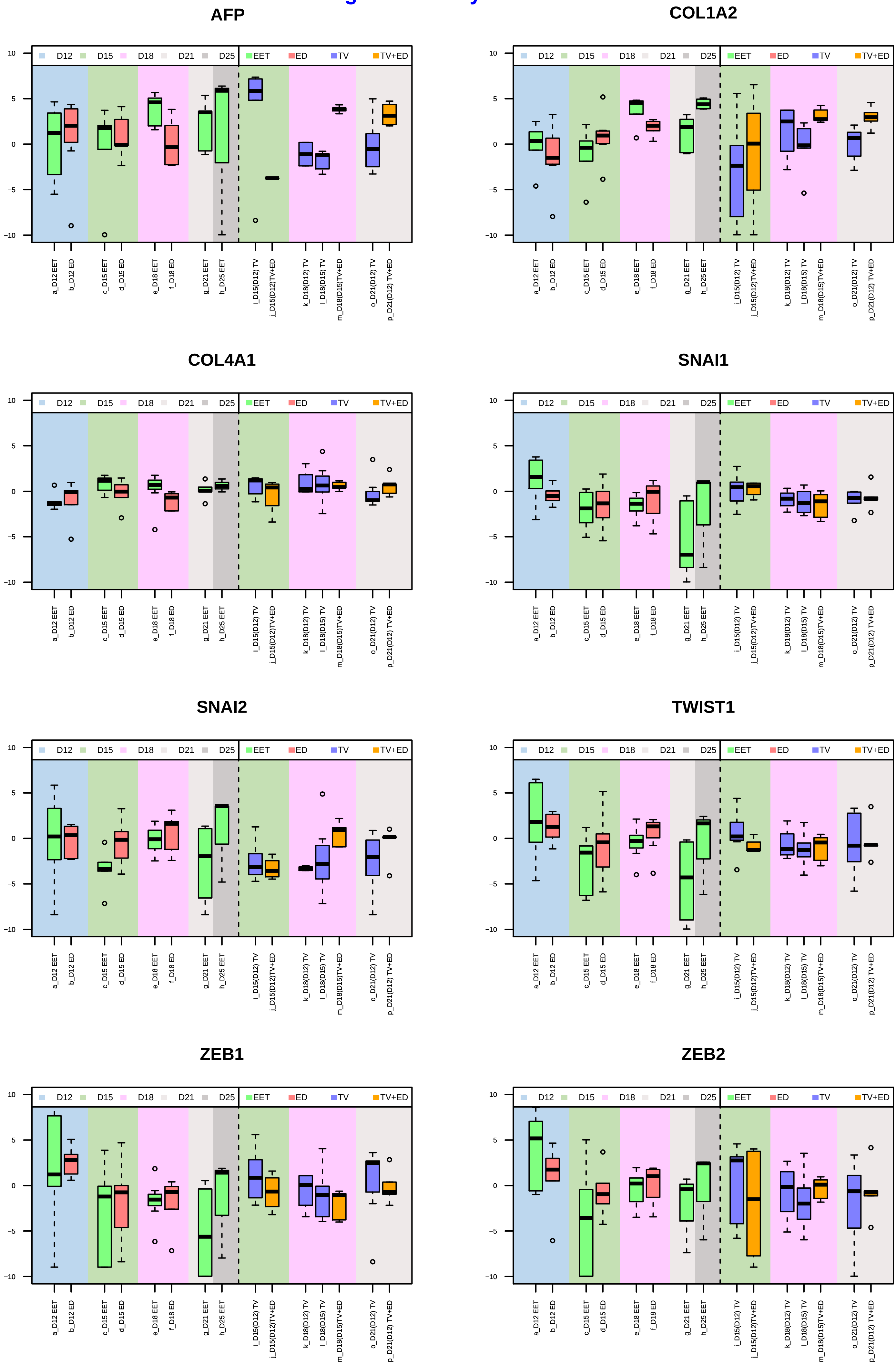

Biological Pathway = EEM

BMP4

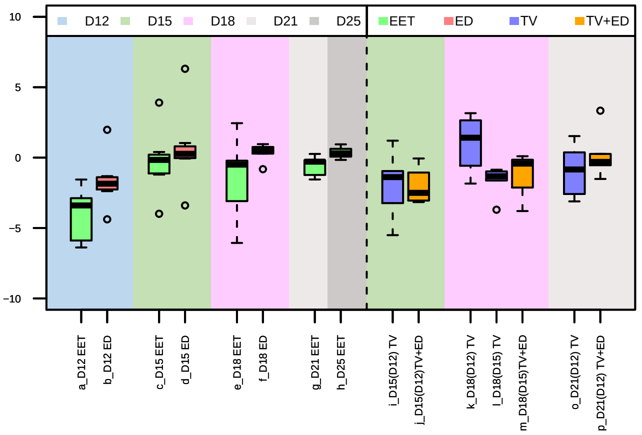

CD44

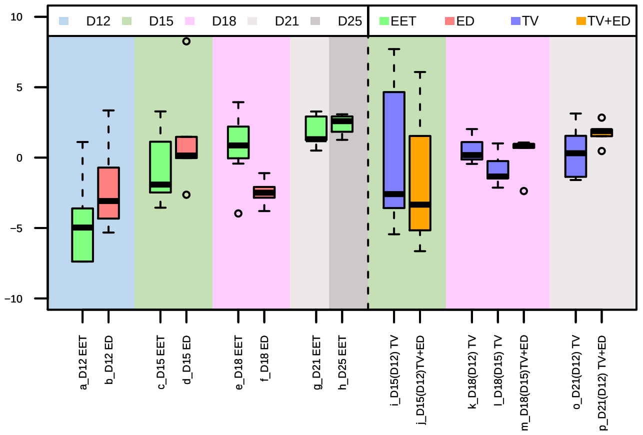

HAND1

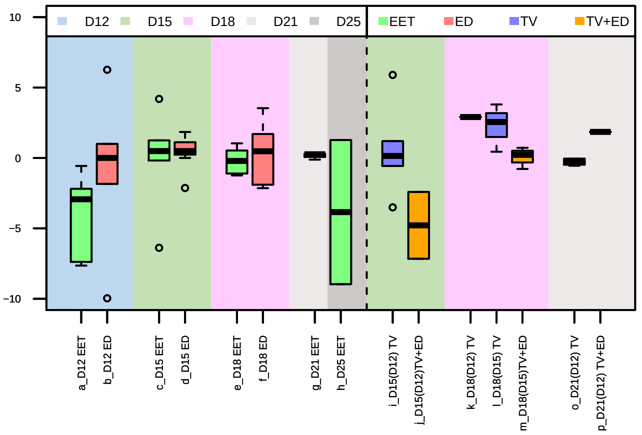

MMP2

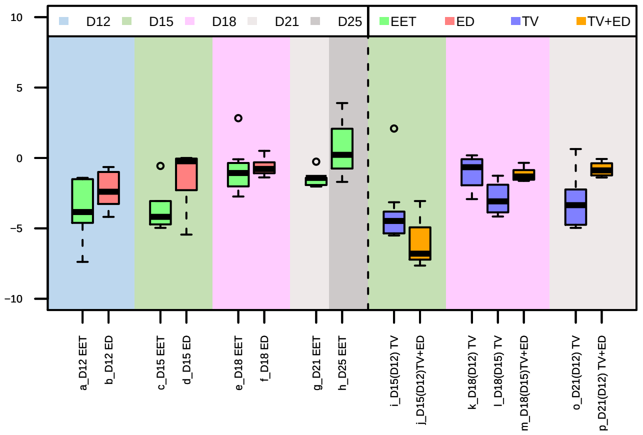

MMP9

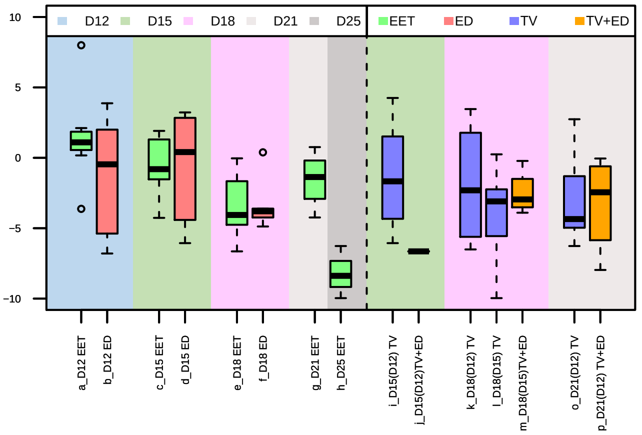

SPARC

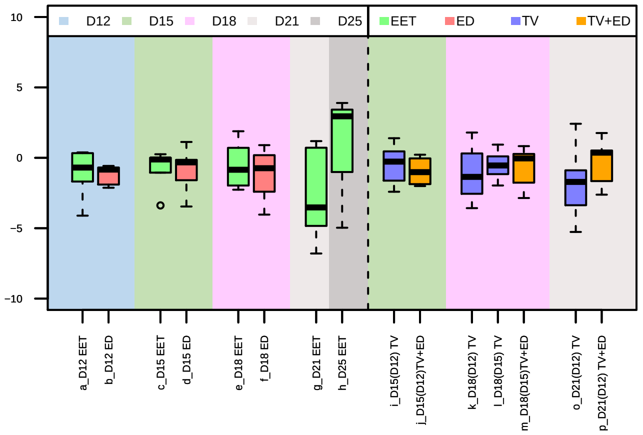

VIM

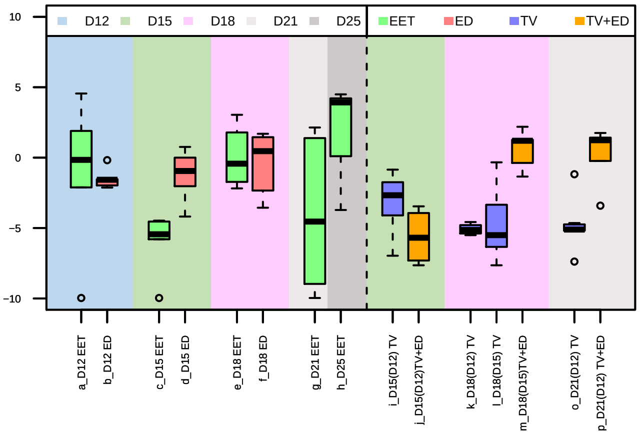

Biological Pathway = BNC

CITED1

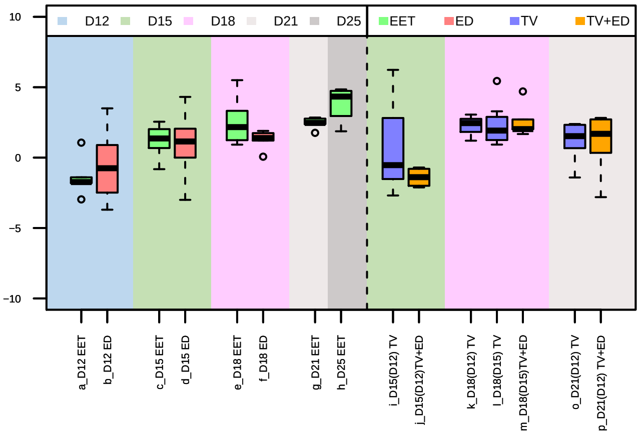

CPA3

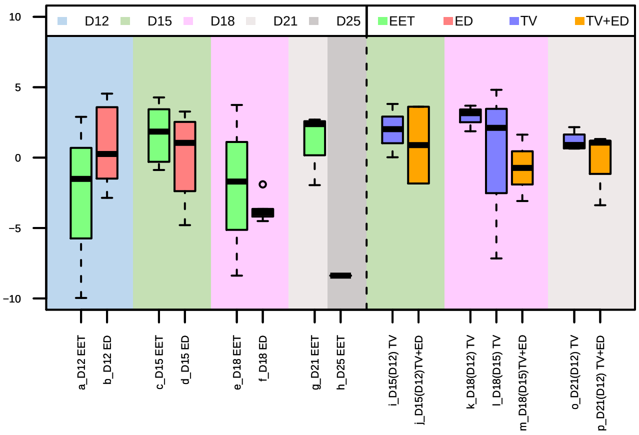

DLX3

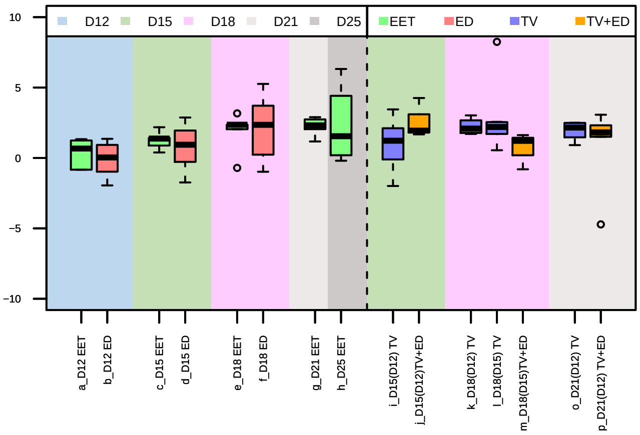

PAG1

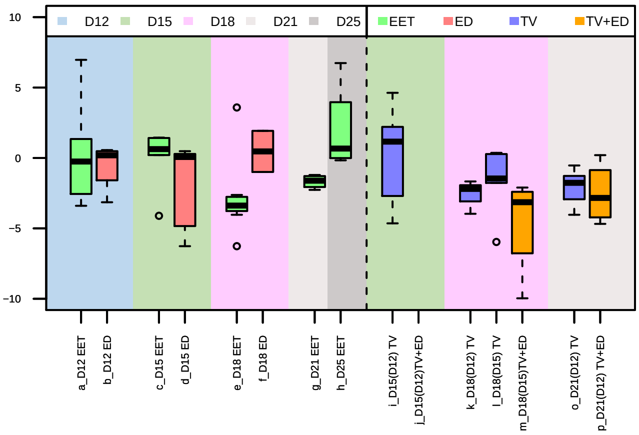

PLIN2

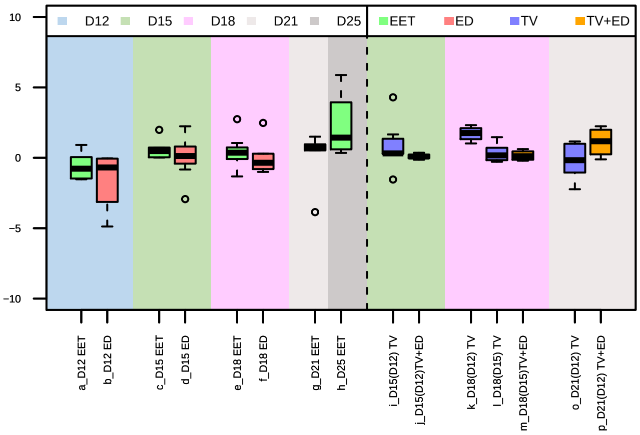

PPARG

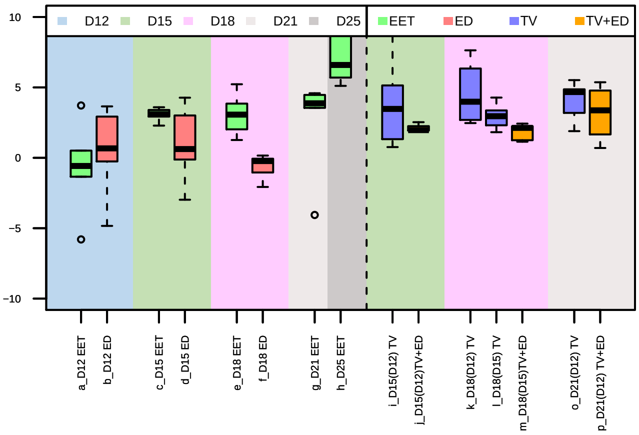

PRL

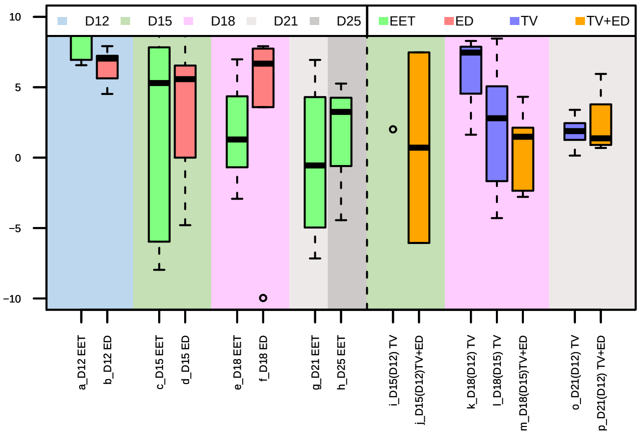

Biological Pathway = Steroidogenesis

CYP2C18

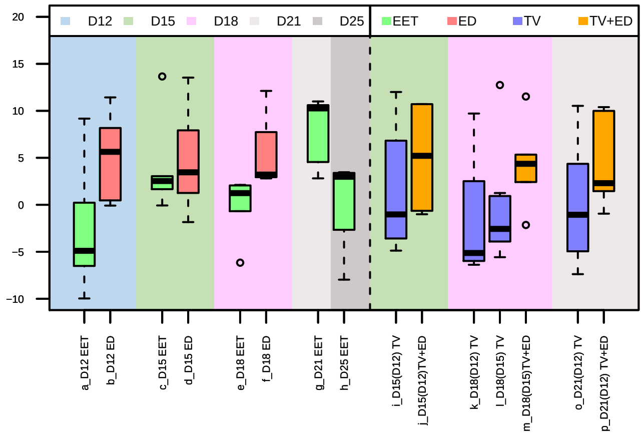

CYP39A1

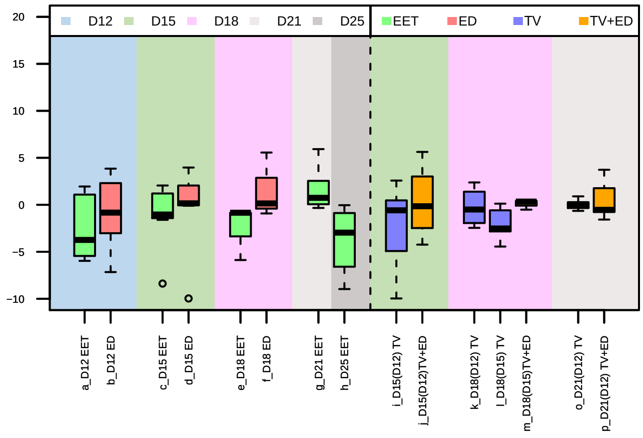

HSD3B1

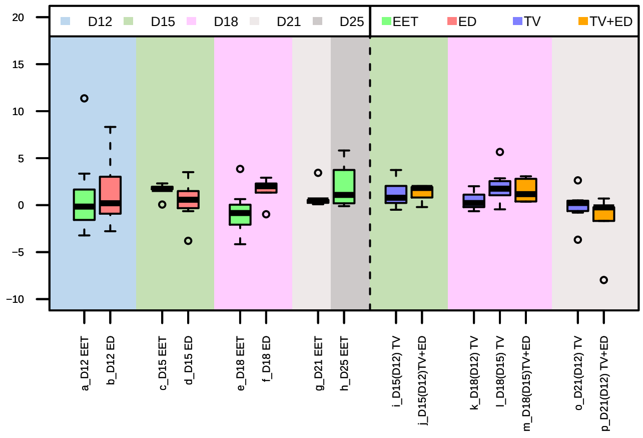

HSD17B2

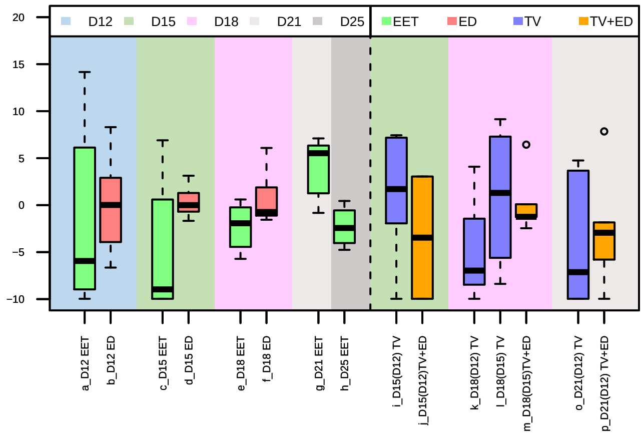

HSD17B10

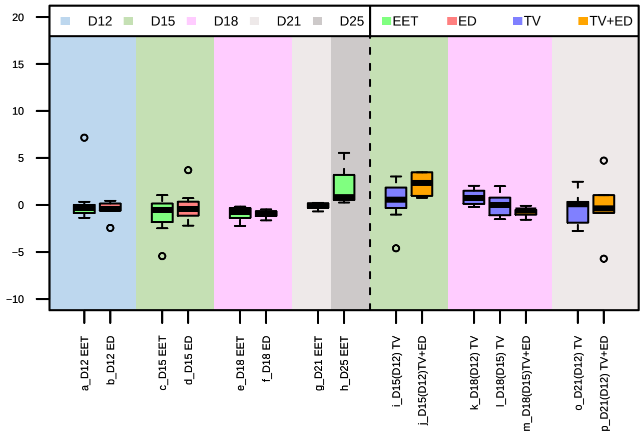

Biological Pathway = Microvilli

EZR

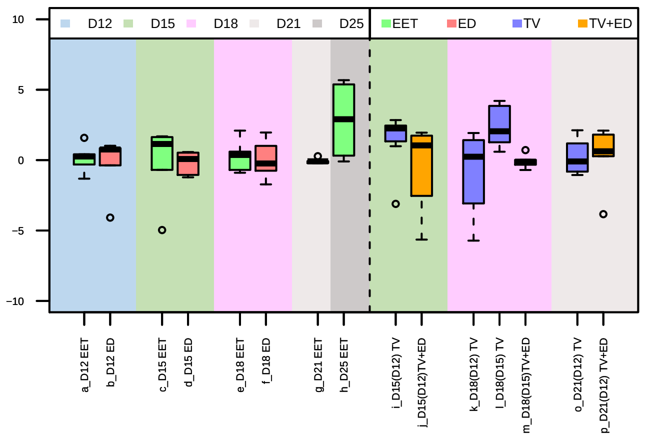

MSN

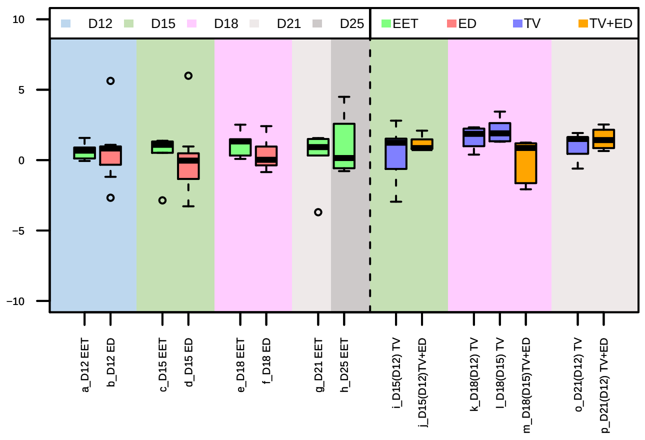

MYO6

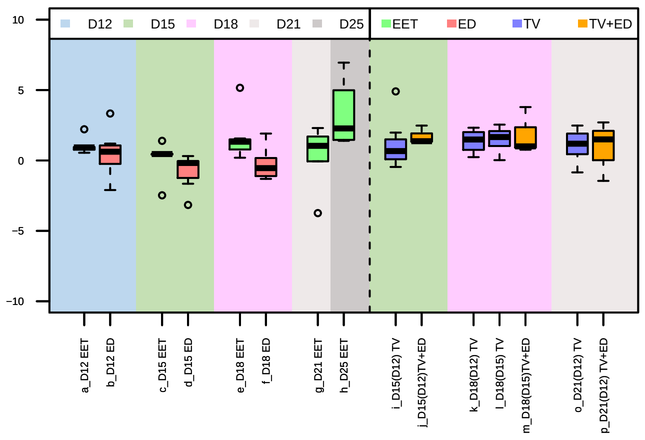

RDX

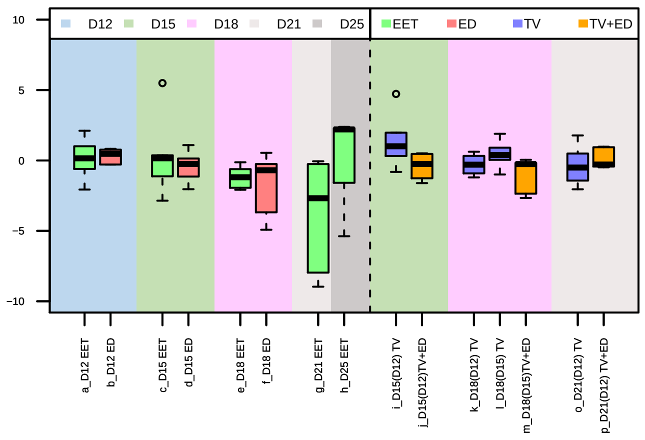

VIL1

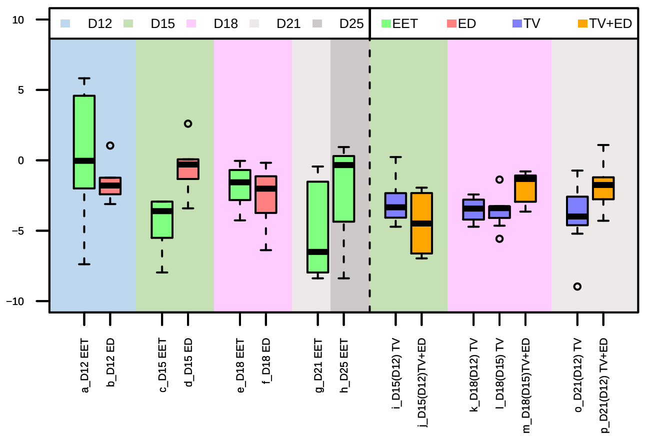

Biological Pathway = Cell Shape

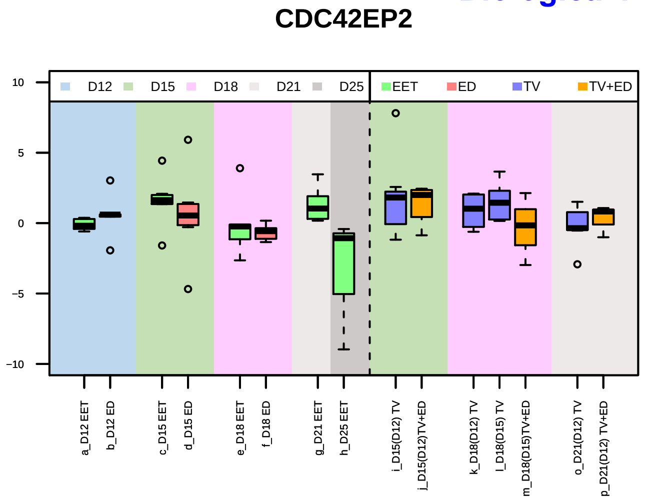

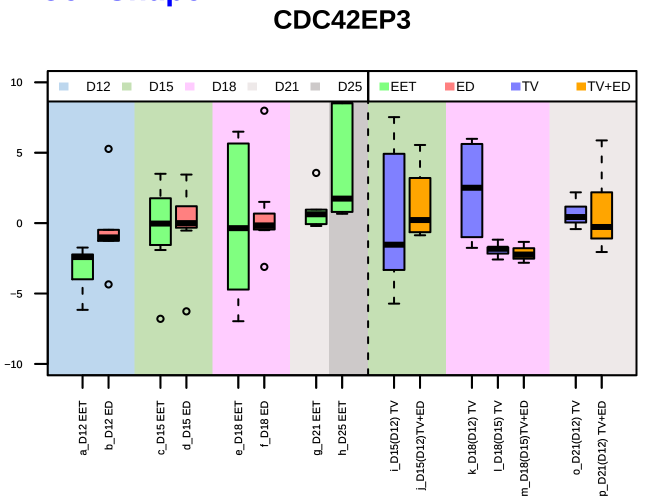

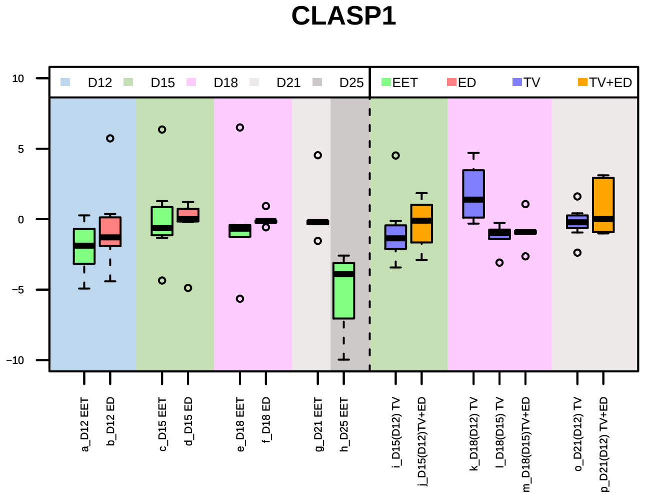

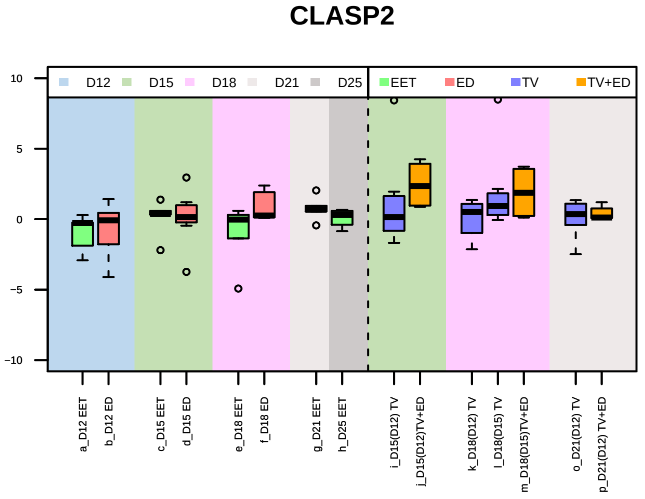

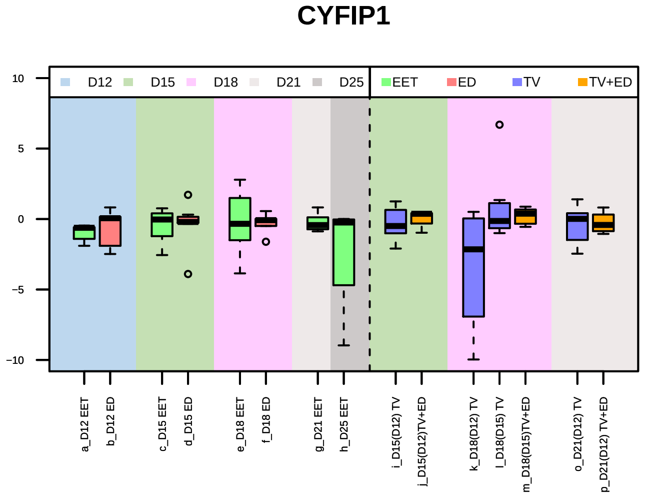

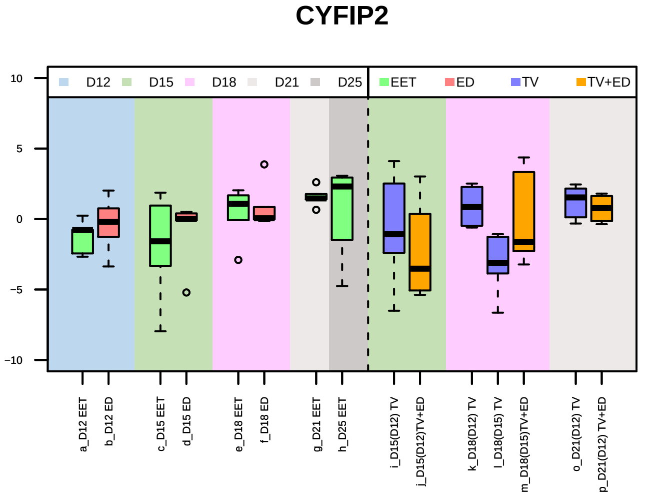

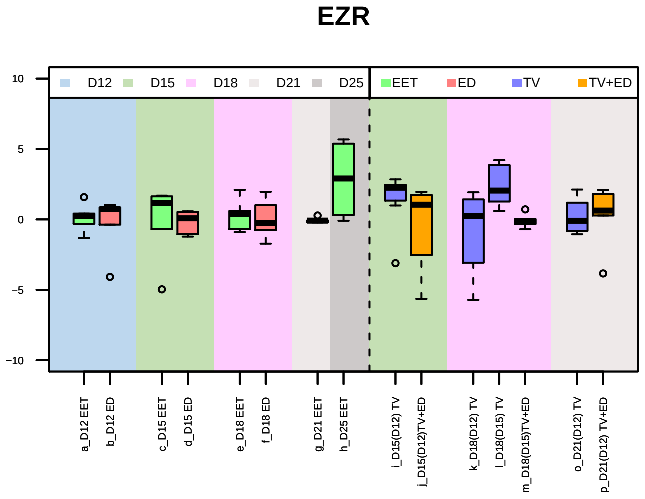

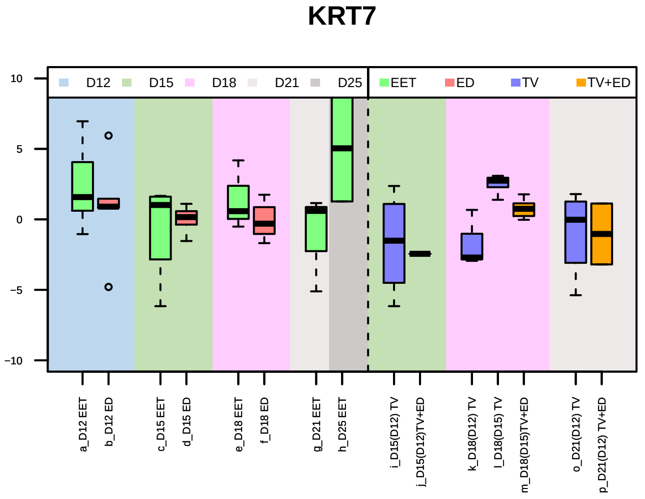

Biological Pathway = Cell Shape

KRT8

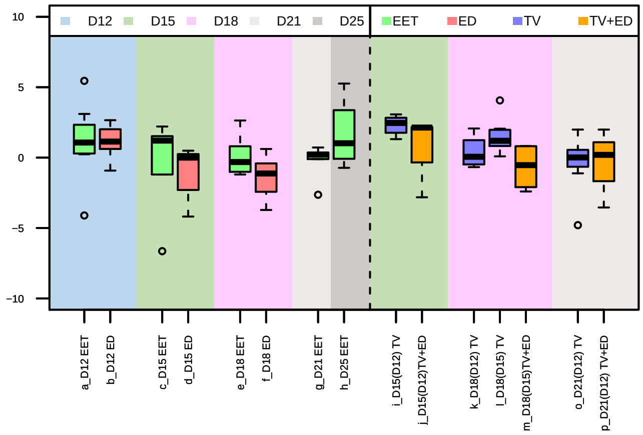

KRT18

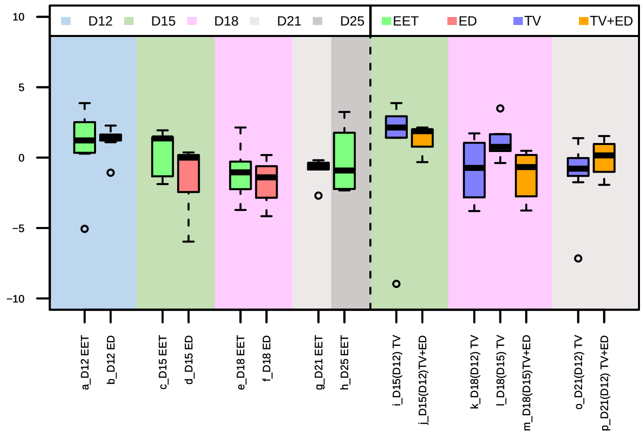

KRT19

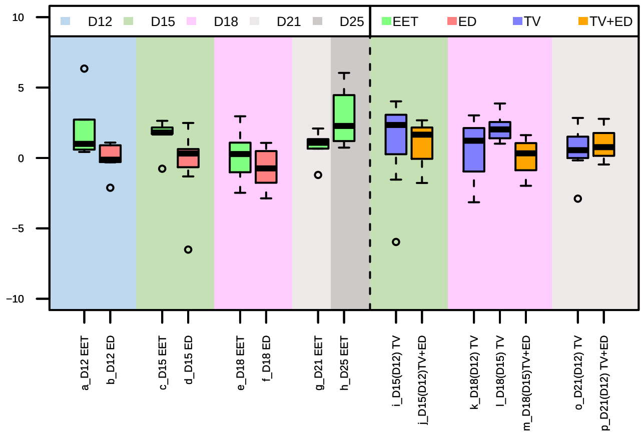

LLGL1

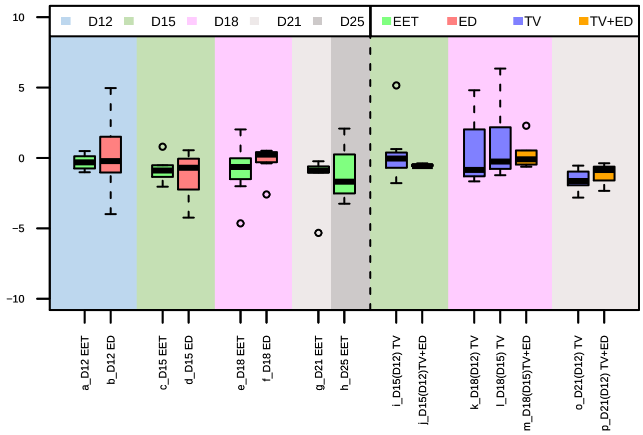

MARK2

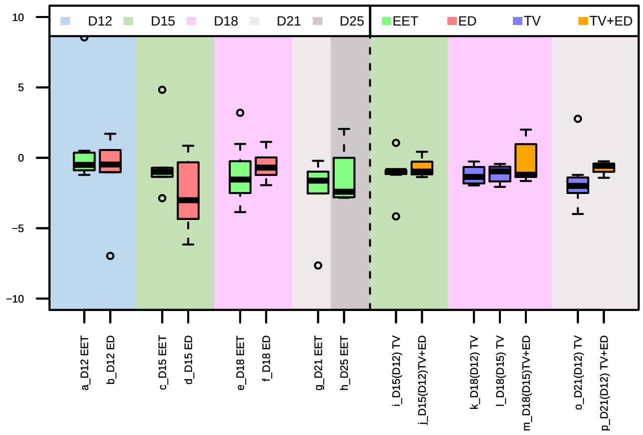

MYLK2

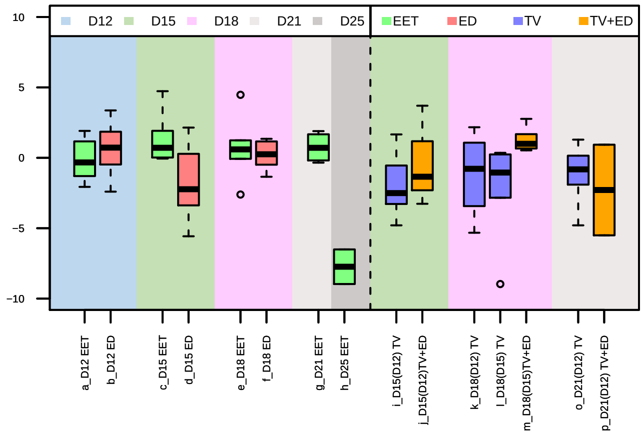

RAC1

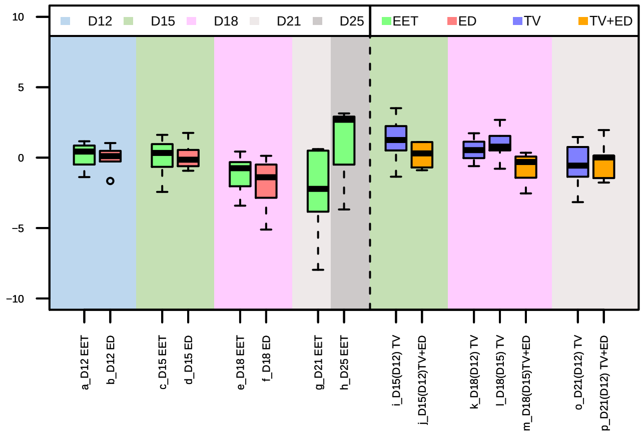

RHOA

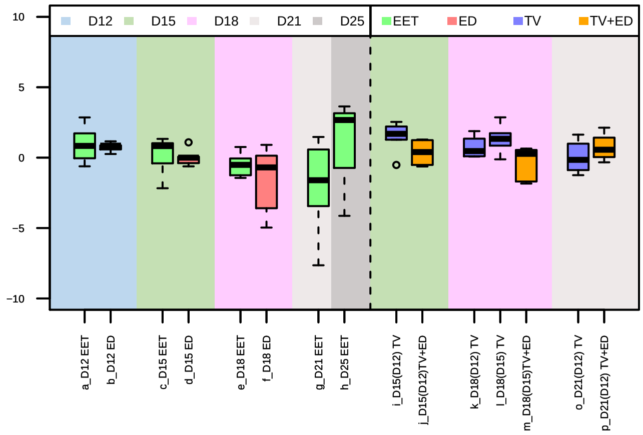

Biological Pathway = Mitosis

AURKA

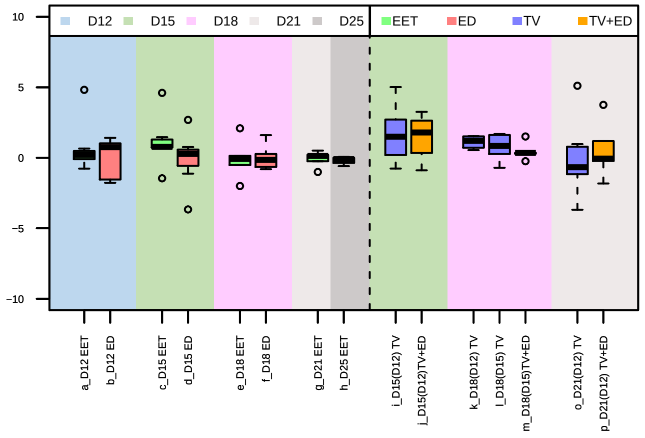

AURKB

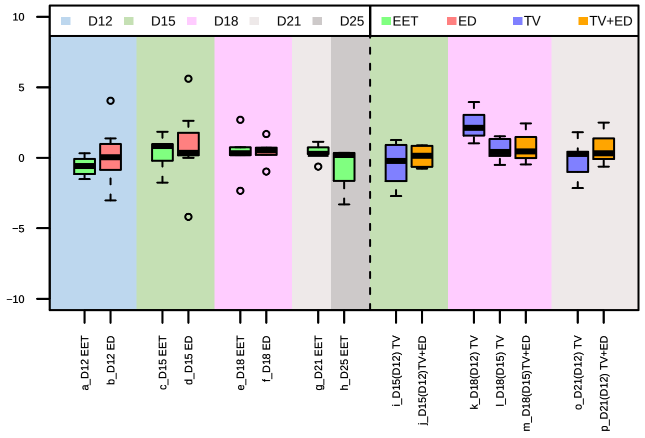

CCND1

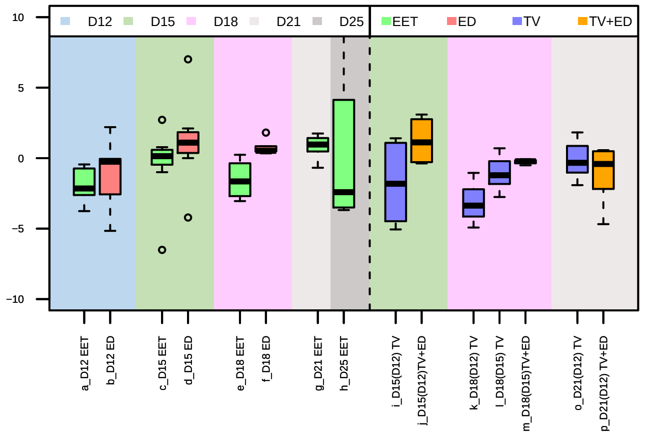

CLASP1

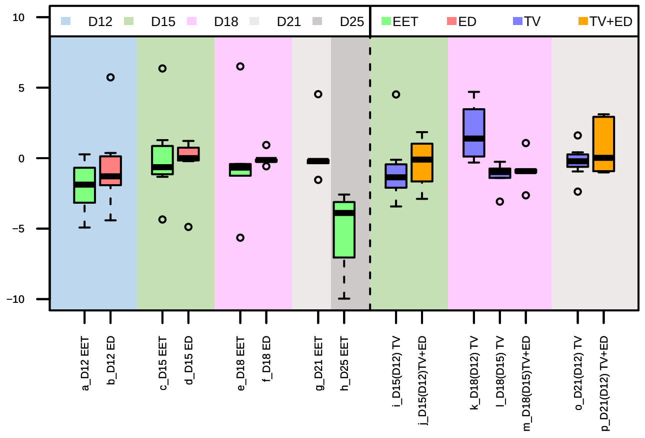

CLASP2

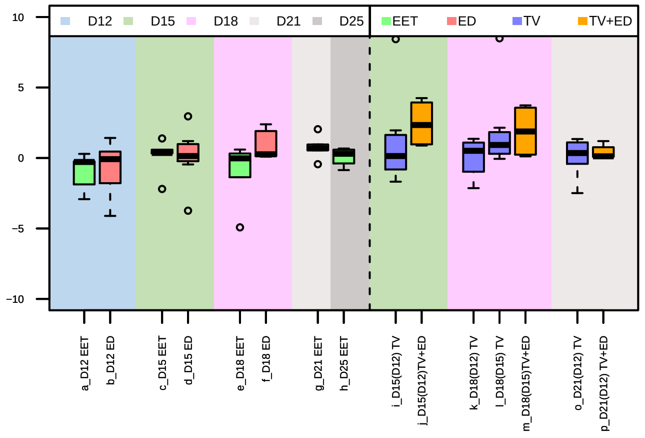

CLIP1

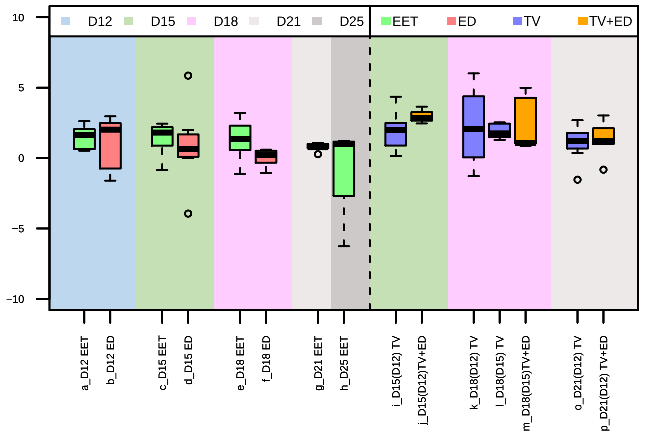

MAPRE1

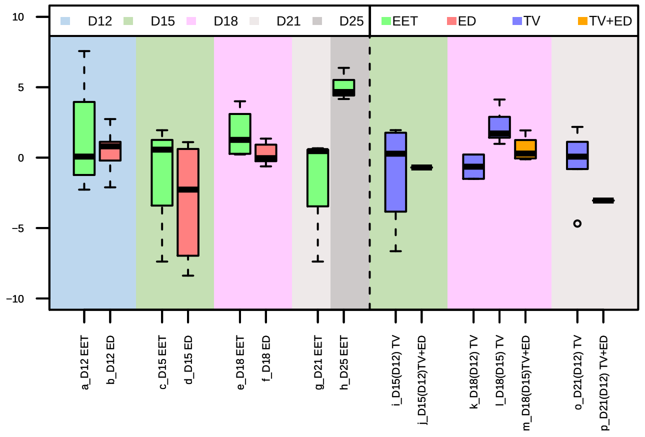

MAPRE2

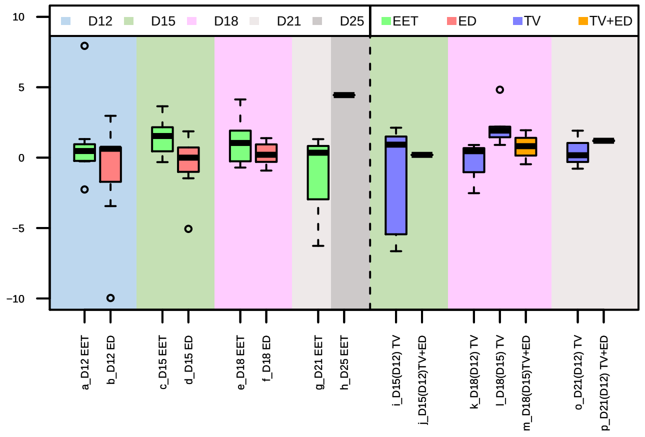

Biological Pathway = Cytokinesis

AURKB

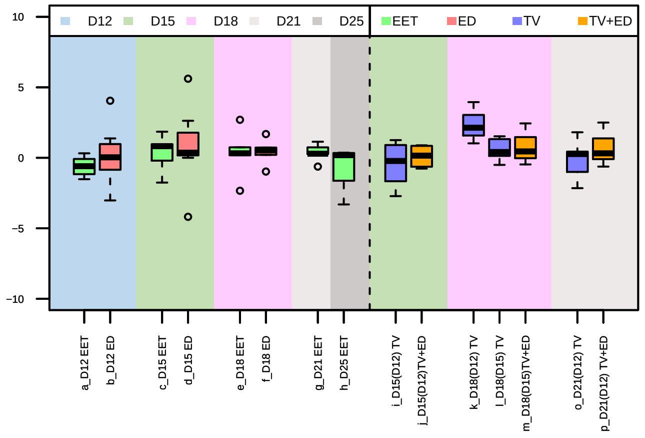

AURKC

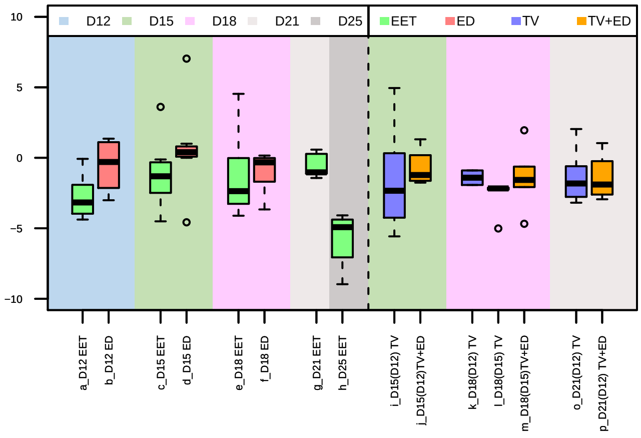

DSTN

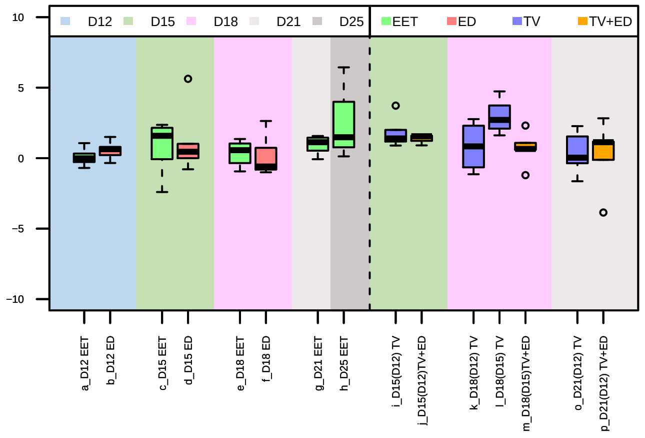

ROCK1

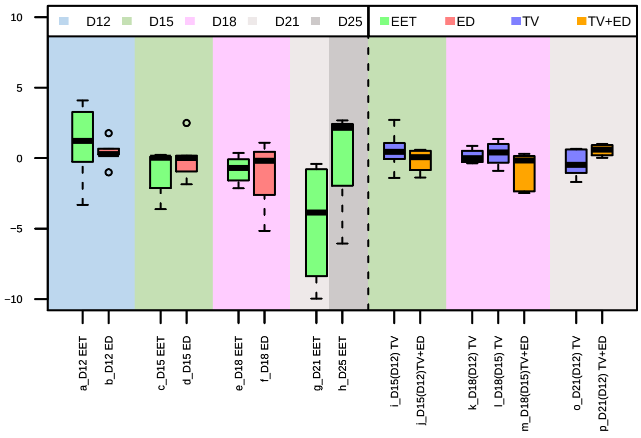

Biological Pathway = Integrins

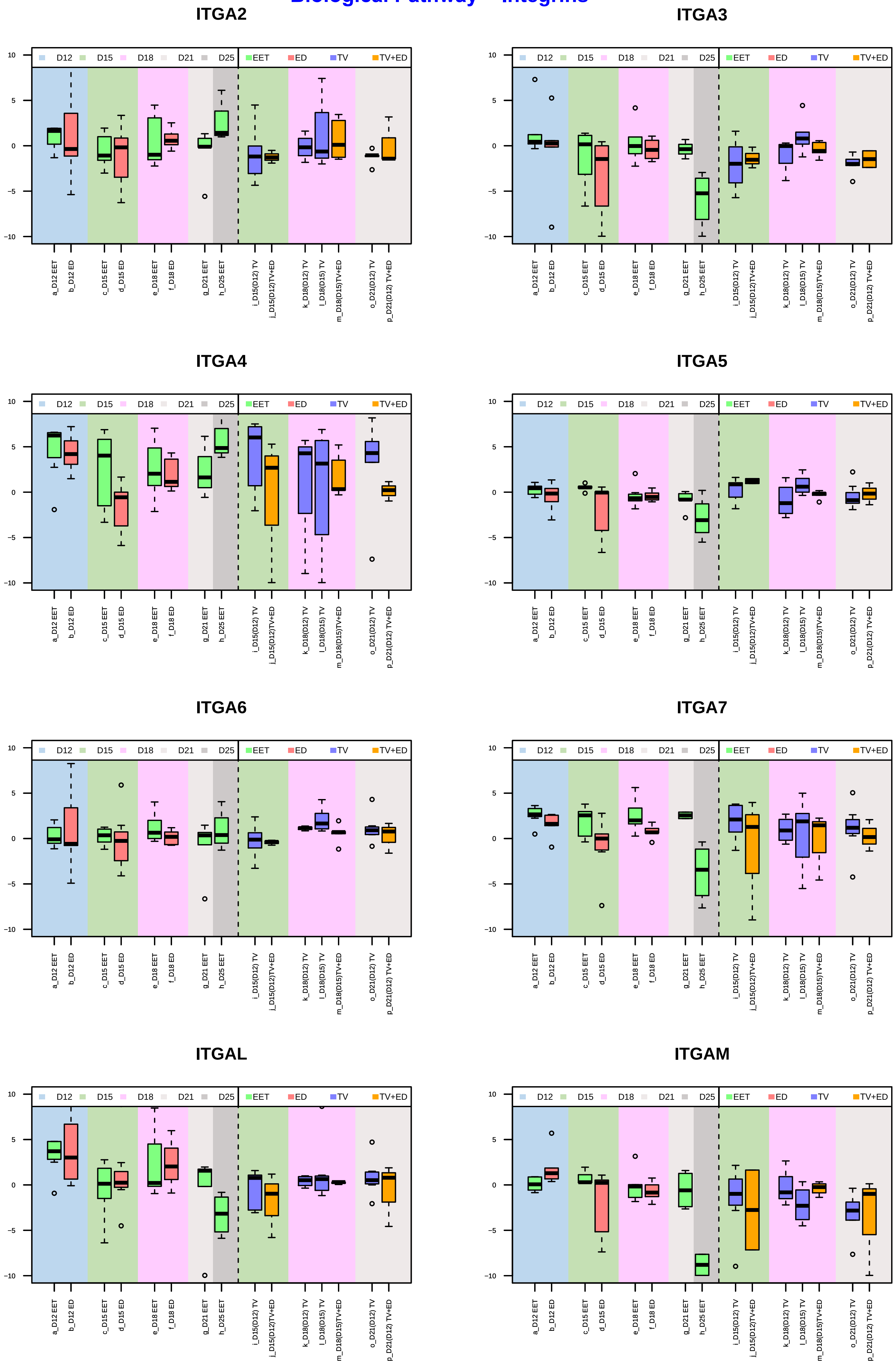

Biological Pathway = Integrins

ITGAV

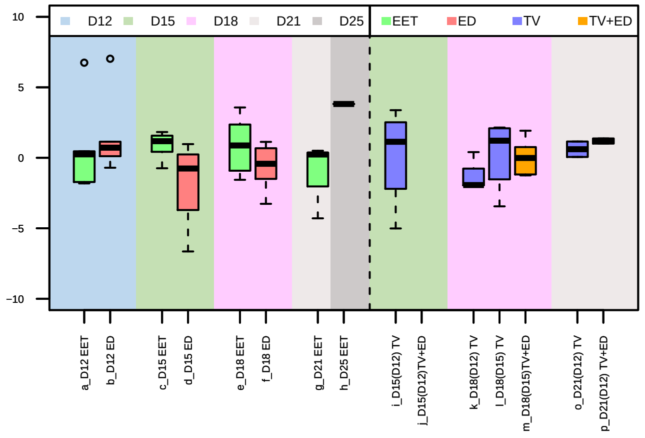

ITGB1

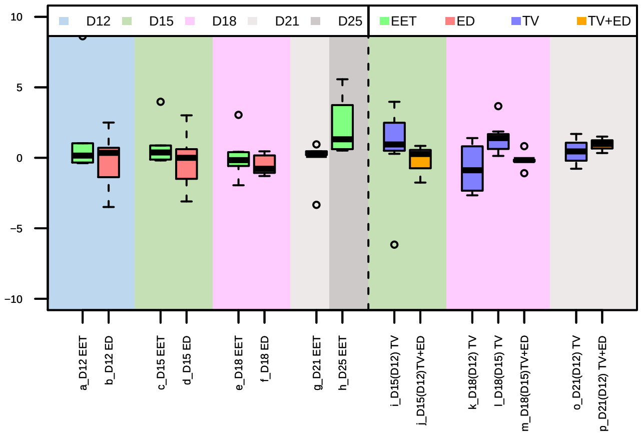

ITGB2

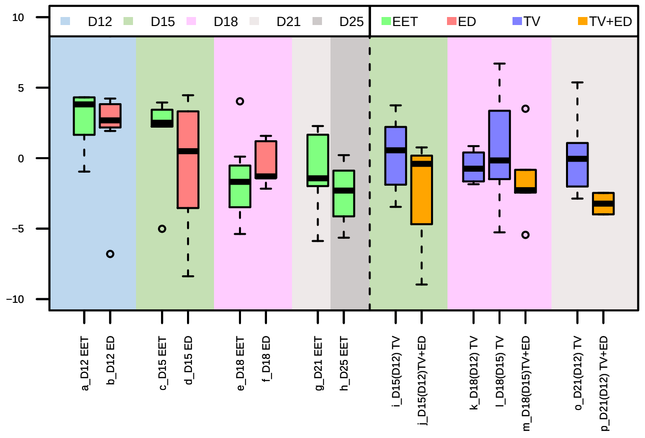

ITGB4

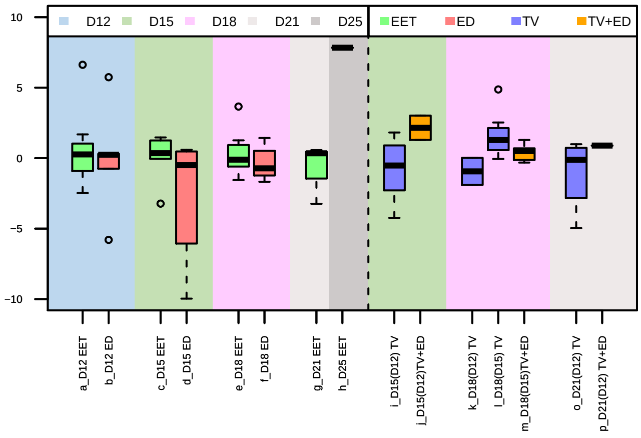

ITGB5

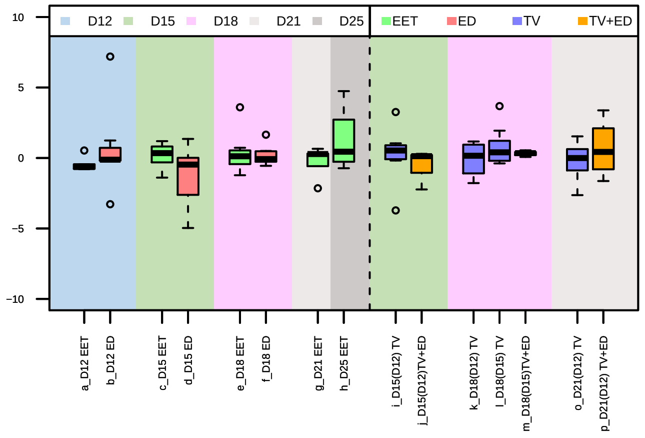

Biological Pathway = ECM

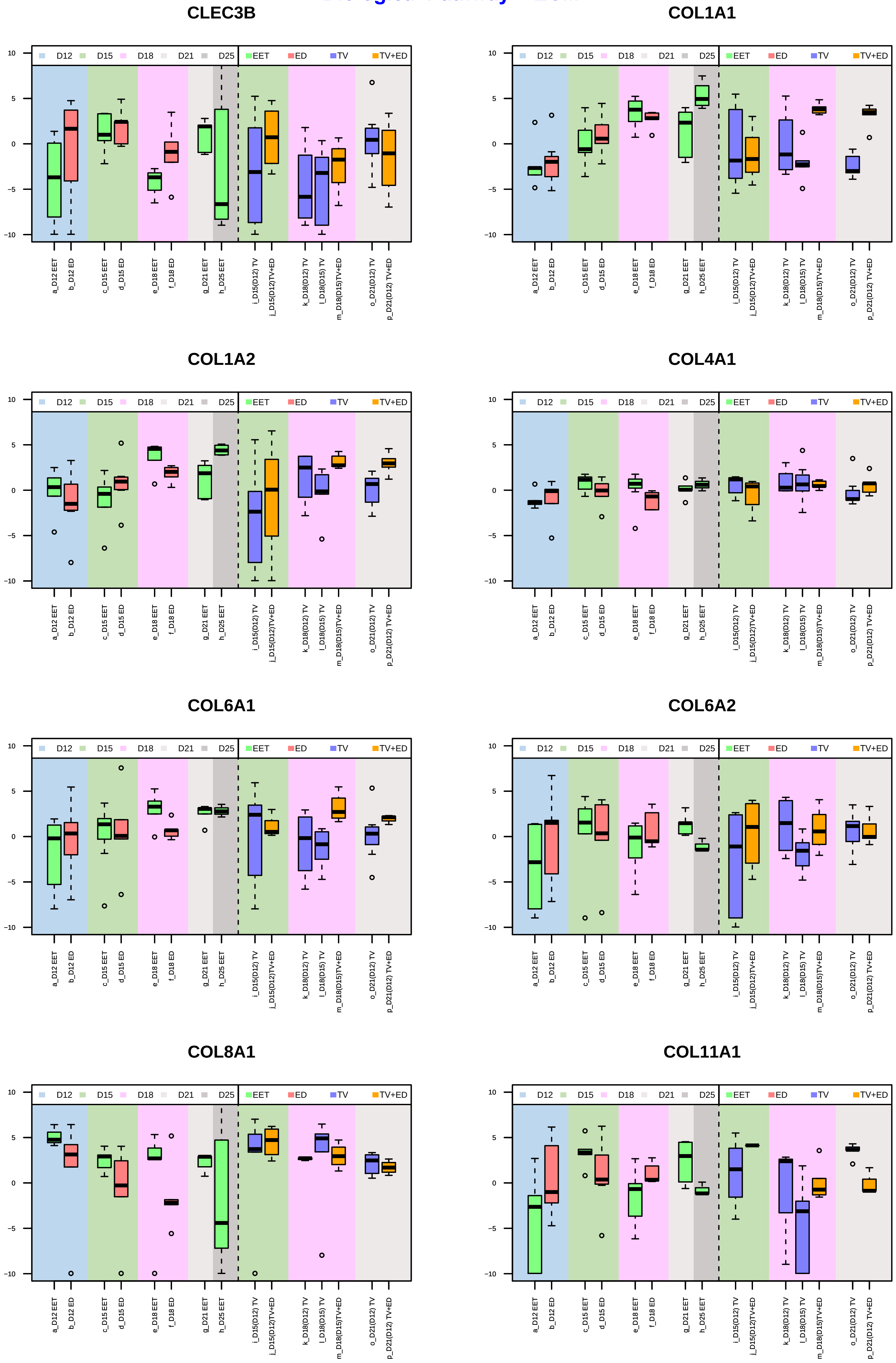

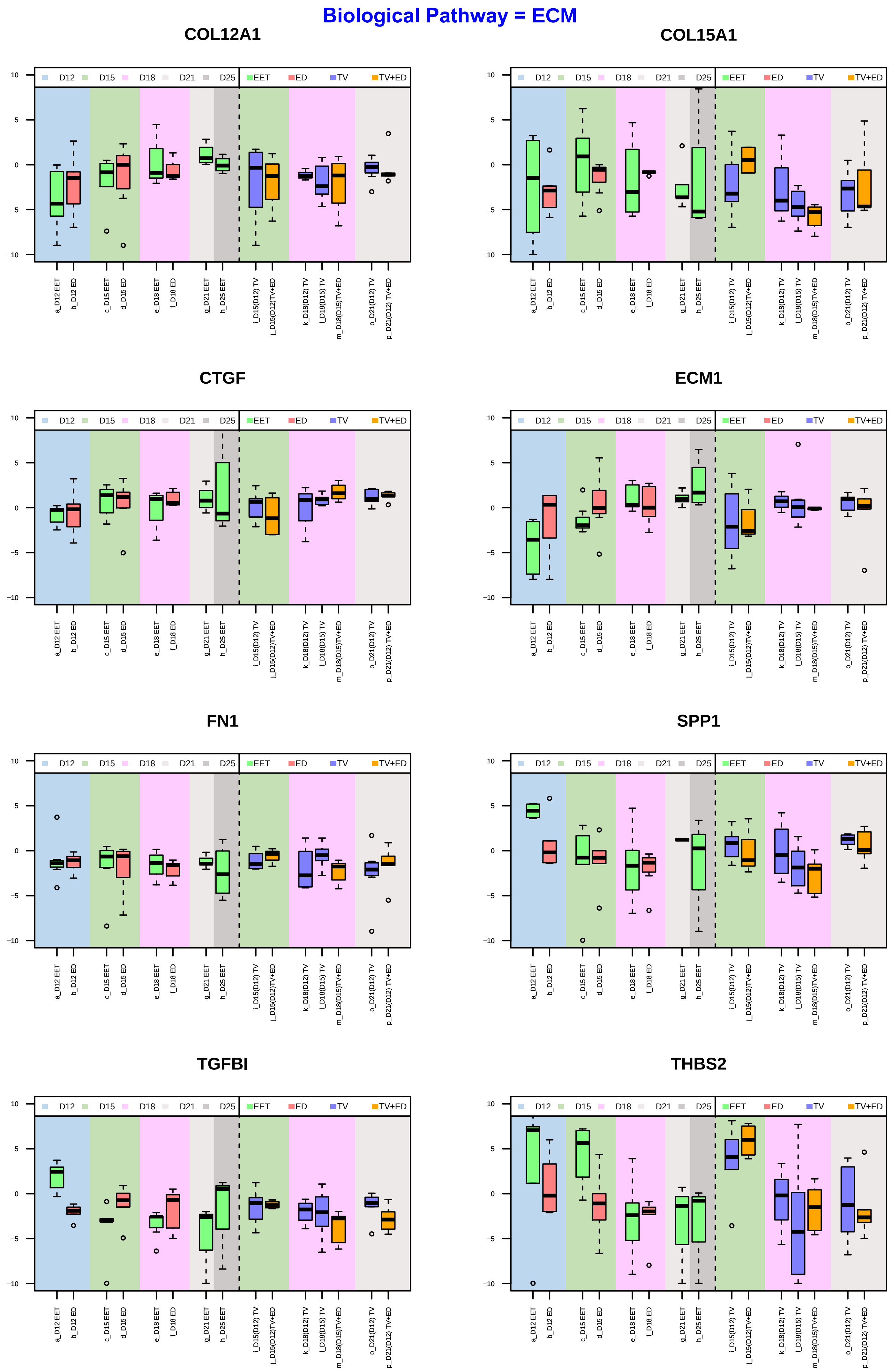
Biological Pathway = ECM
COL12A1
COL15A1
CTGF
ECM1
FN1
SPP1
TGFBI
THBS2
D12
D15
D18
D21
D25
EET
ED
TV
TV+ED
10
5
0
-5
-10
a_D12 EET
b_D12 ED
c_D15 EET
d_D15 ED
e_D18 EET
f_D18 ED
g_D21 EET
h_D25 EET
i_D15(D12) TV
j_D15(D12)TV+ED
k_D18(D12) TV
l_D18(D15) TV
m_D18(D15)TV+ED
o_D21(D12) TV
p_D21(D12) TV+ED

Biological Pathway = ECM

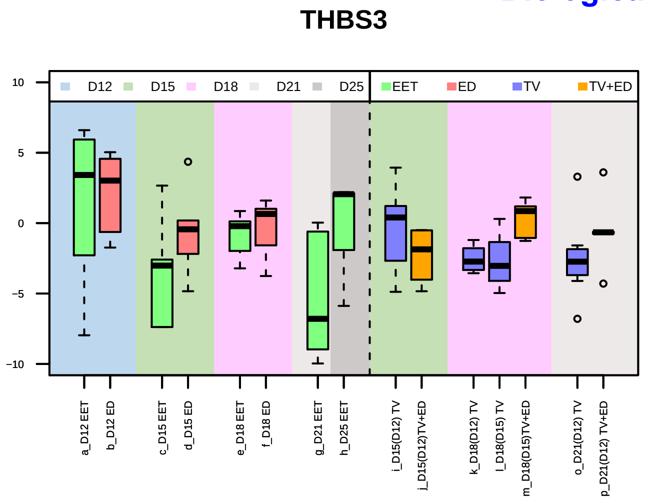

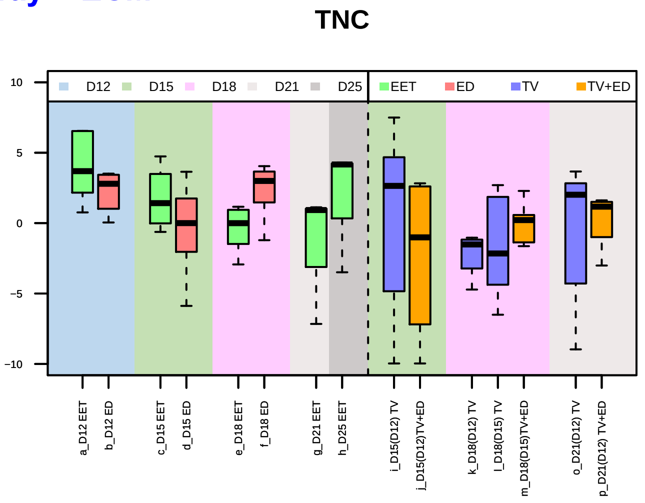

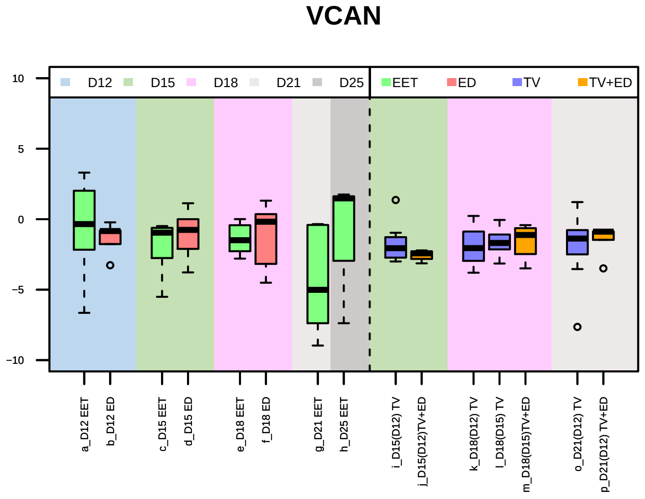

Biological Pathway = Cell Junctions

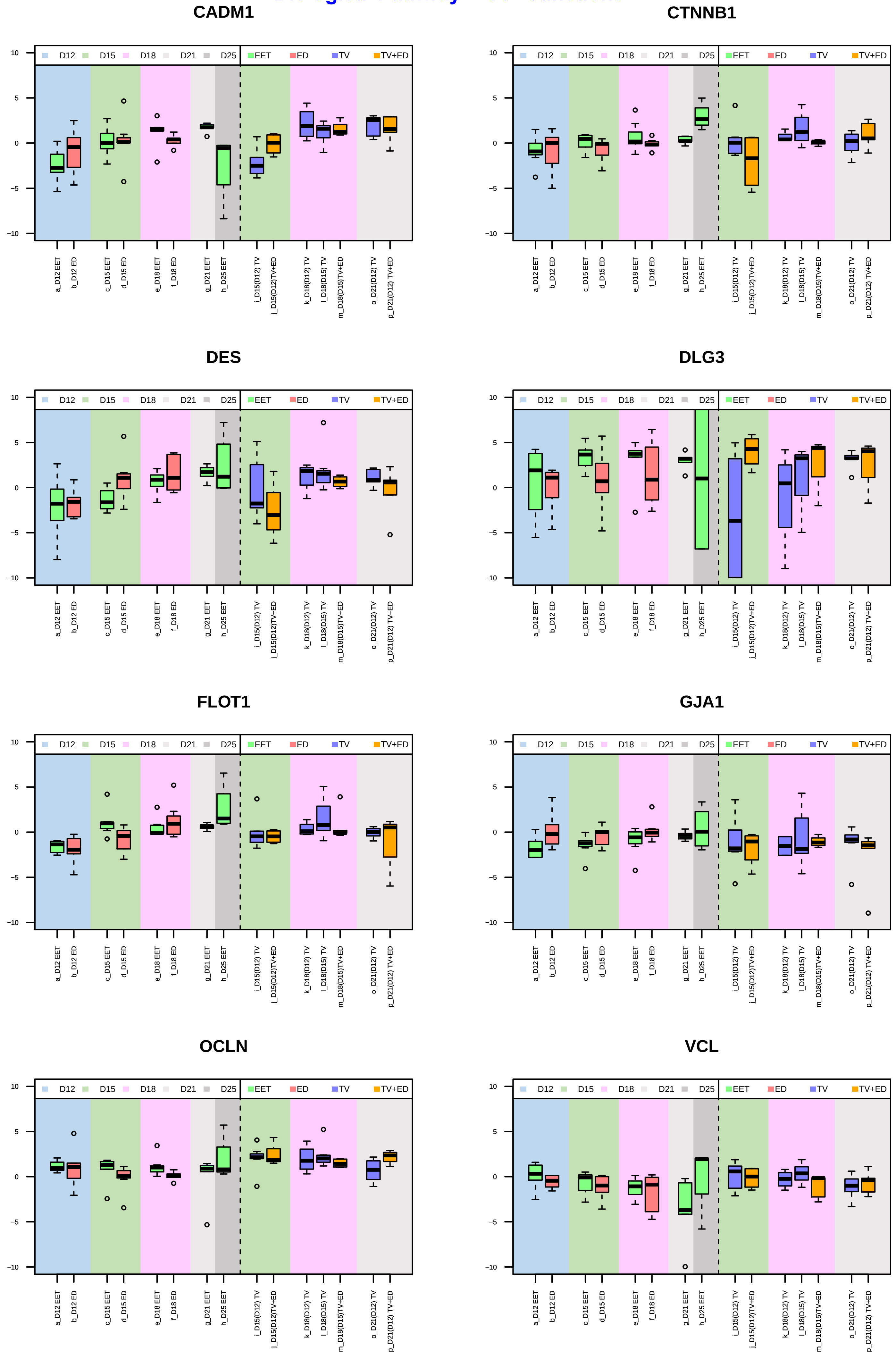

Biological Pathway = Nutrients

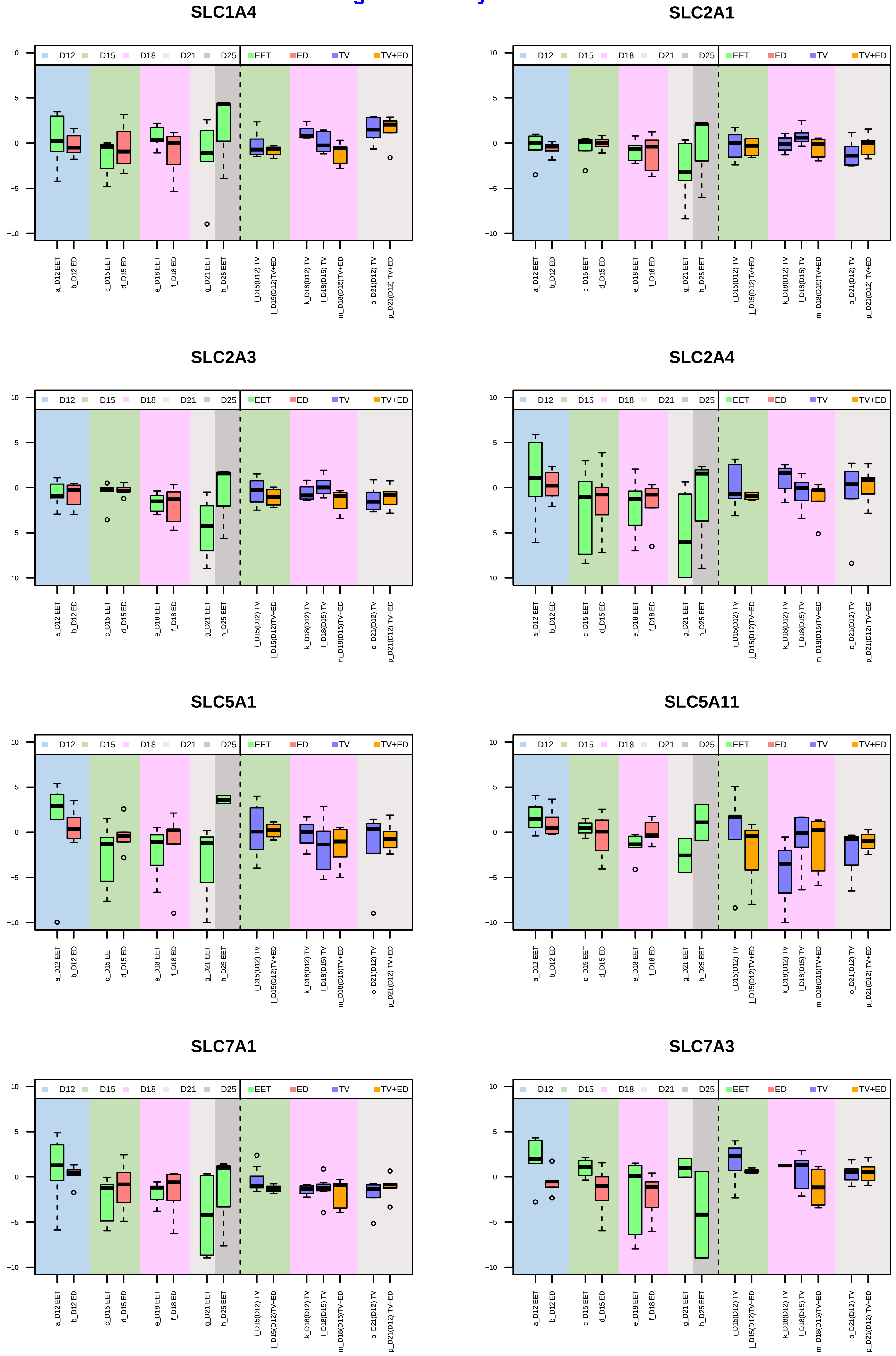

Biological Pathway = Nutrients

SLC7A5

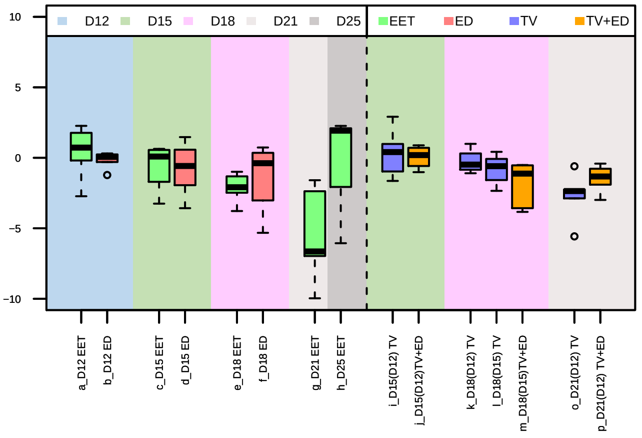

SLC7A6

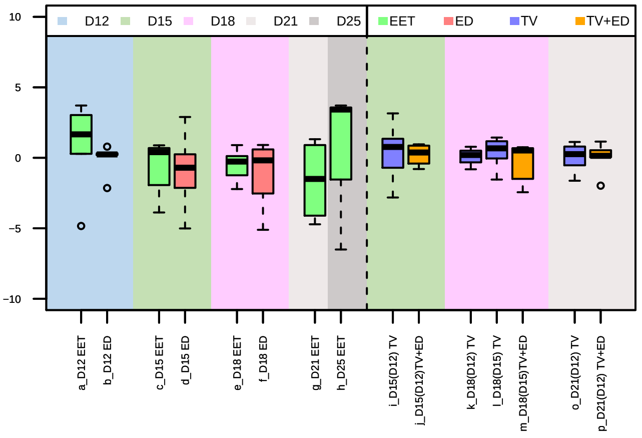

SLC27A1

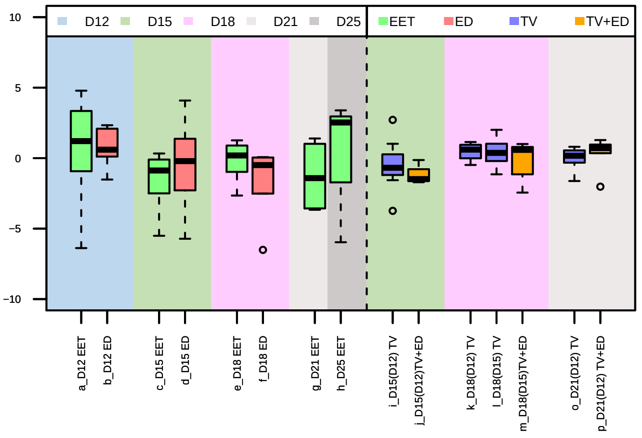

SLC38A7

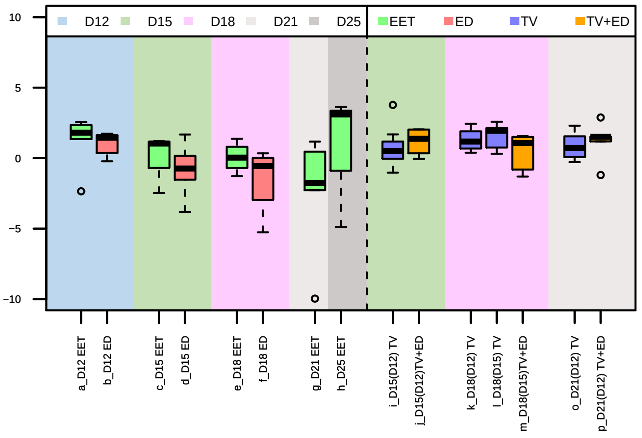

SLC38A11

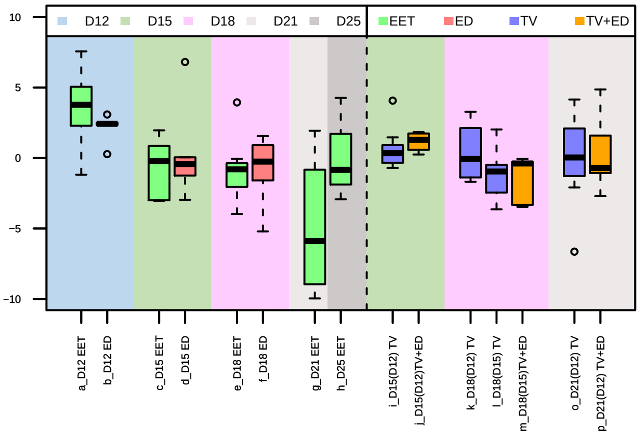

Biological Pathway = FA-AA Metab

ACADL

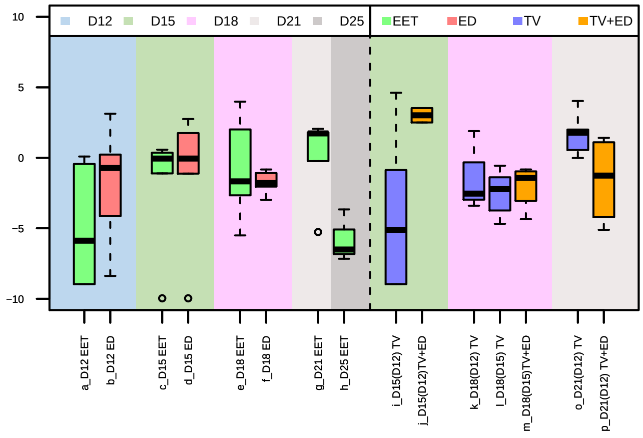

ACADM

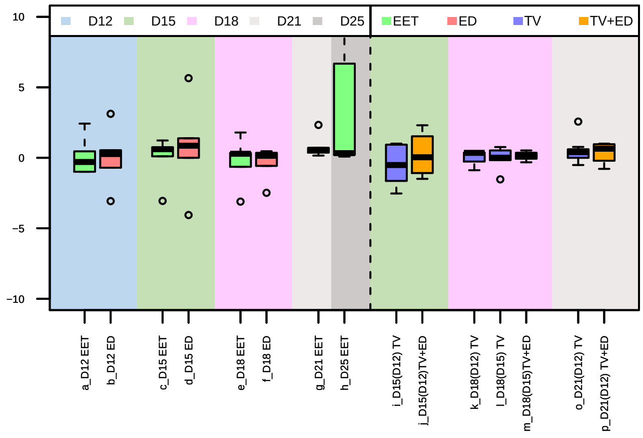

ACADS

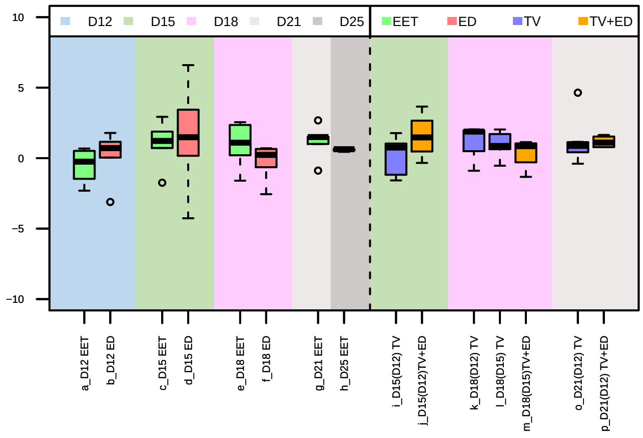

ACADSB

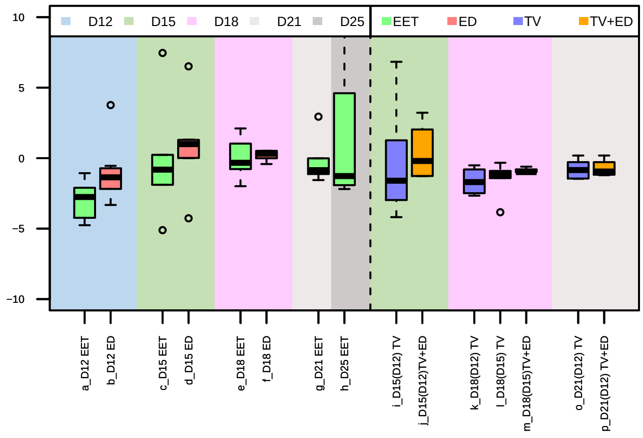

ACAT2

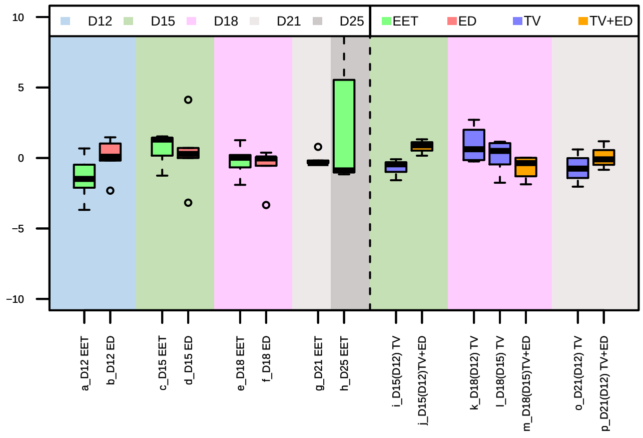

ACOX1

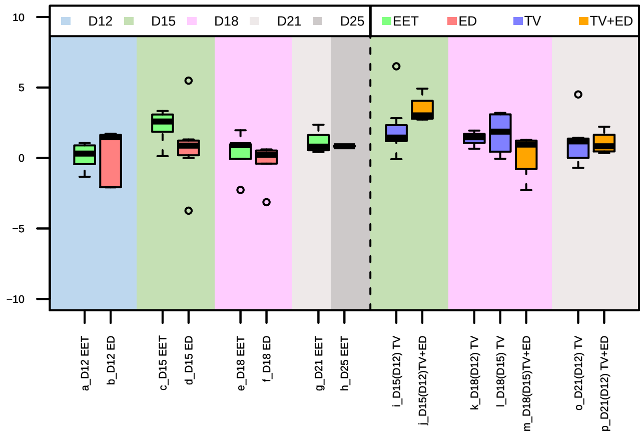

ACOX3

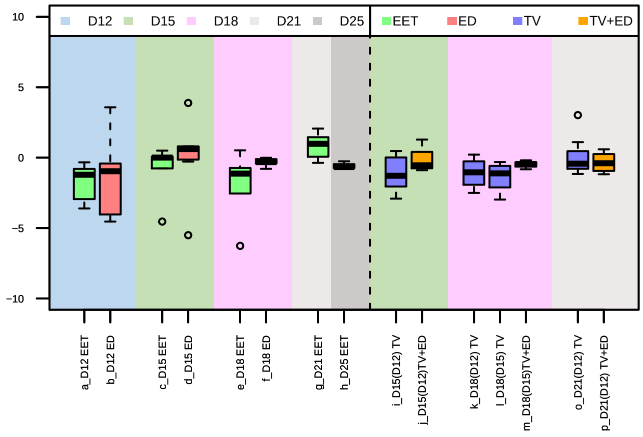

ACSL1

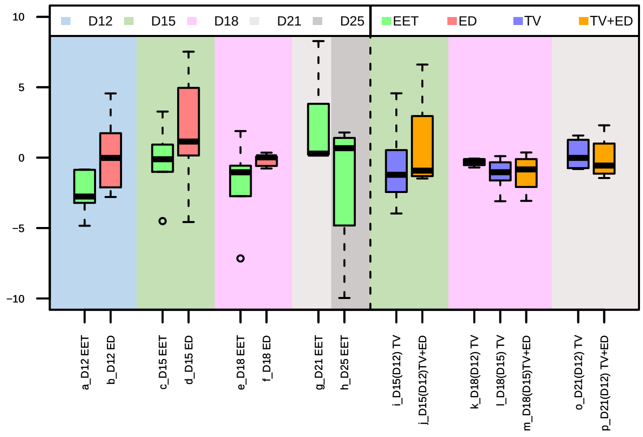

Biological Pathway = FOXO Metab

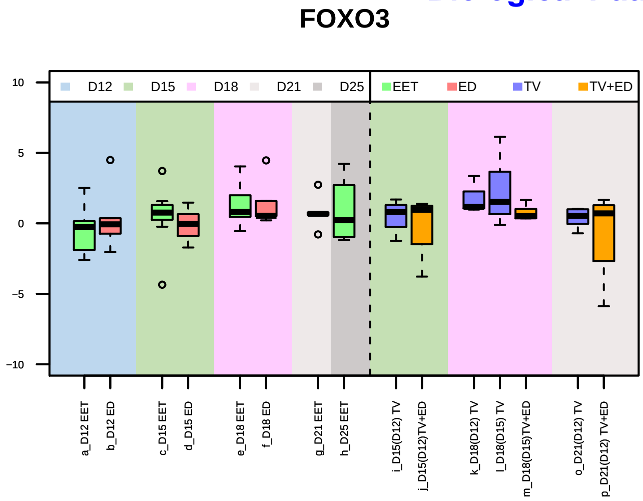

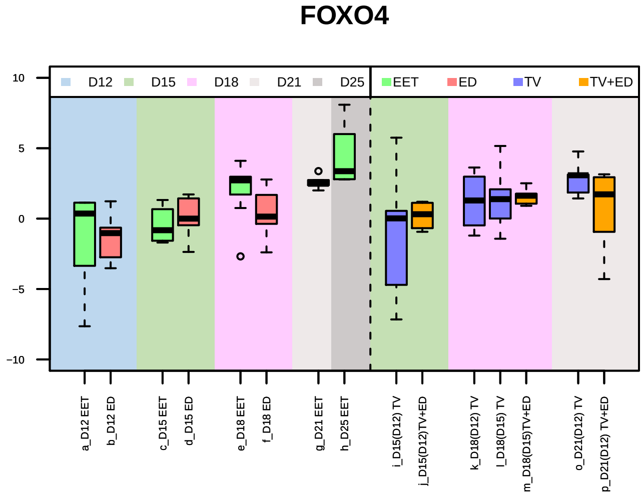

Biological Pathway = PPAR

MED1

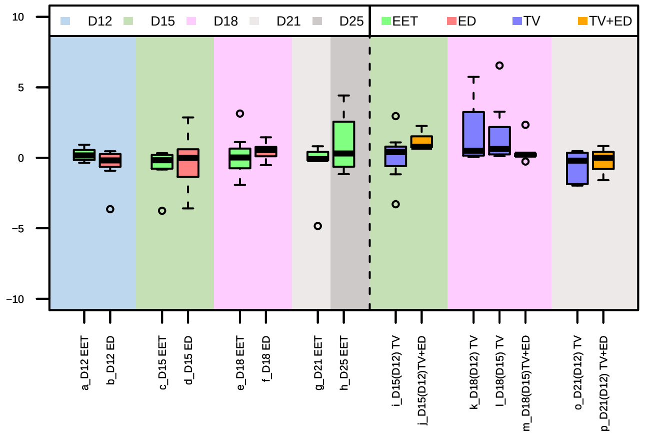

PPARA

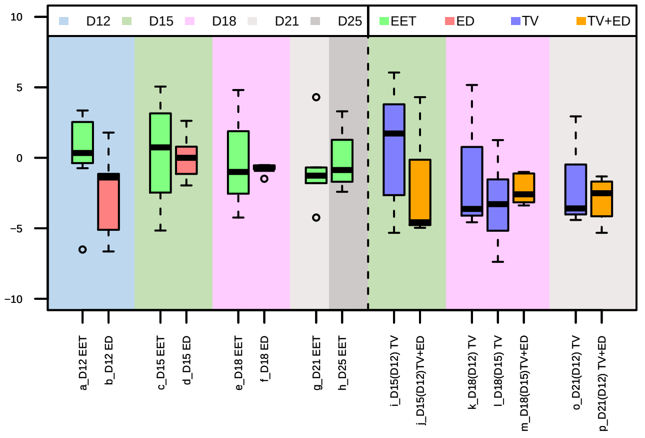

PPARD

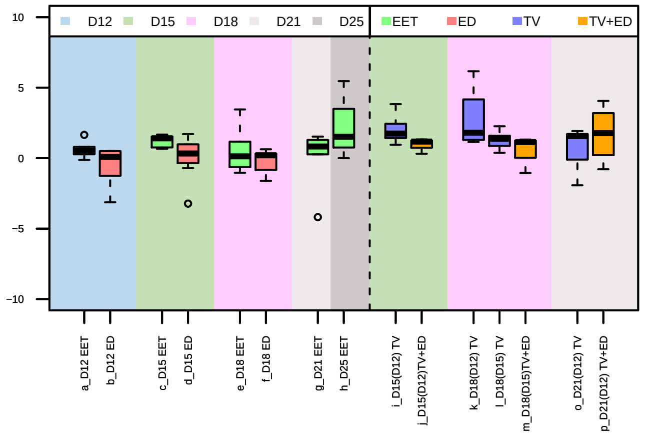

PPARG

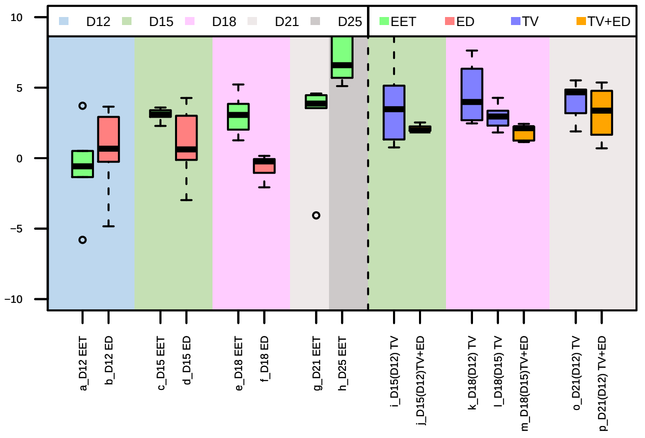

PPARGC1A

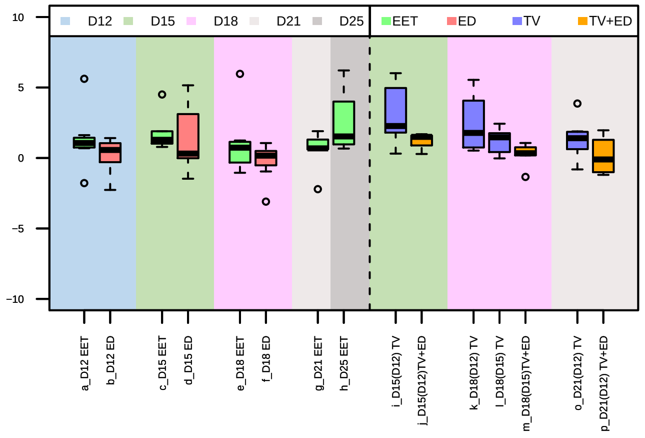

PTGS2

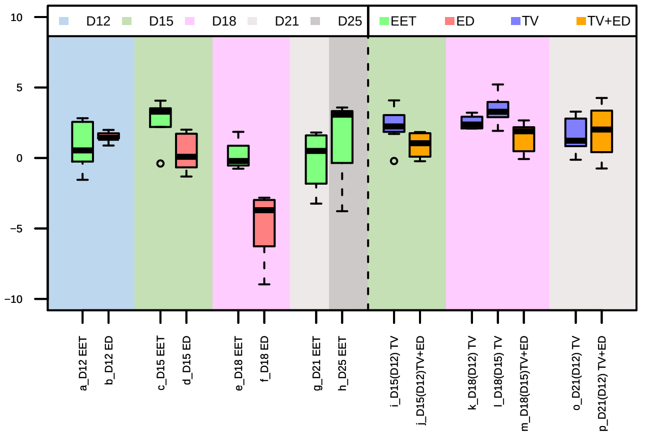

RXRA

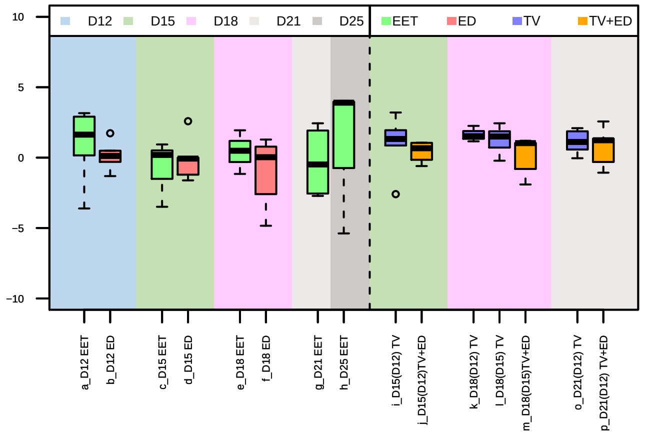

RXRB

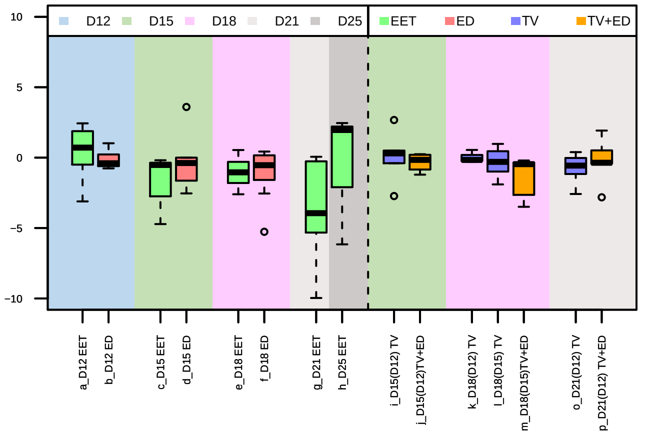

Biological Pathway = PPAR

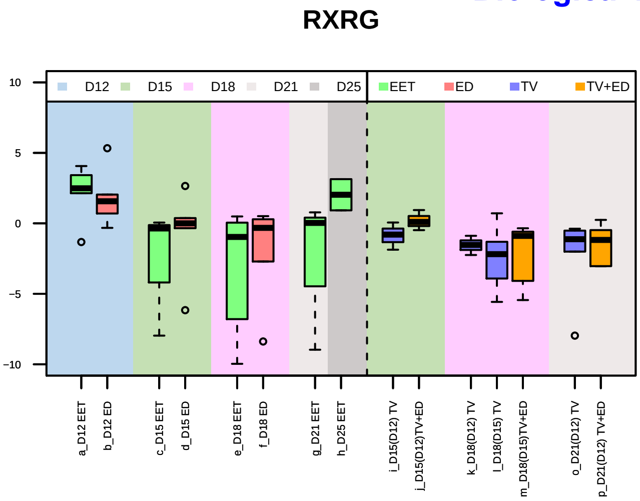

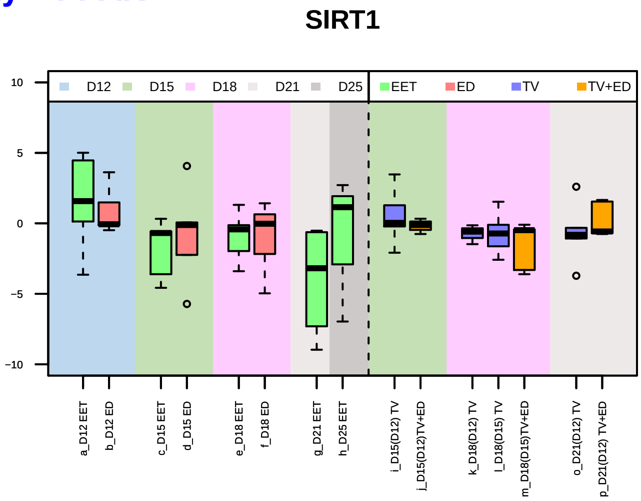

Biological Pathway = Gut Epith

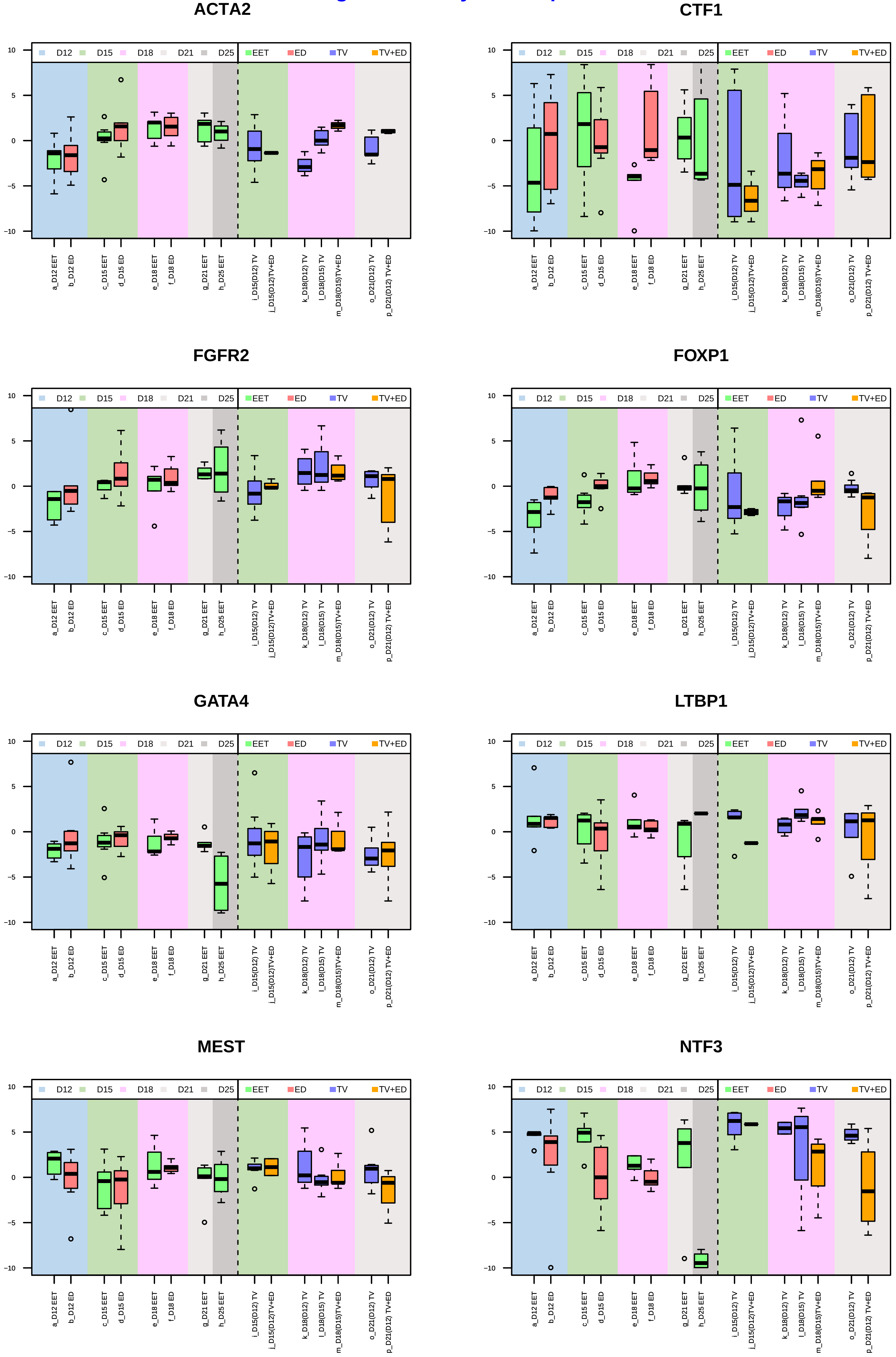

Biological Pathway = Gut Epith

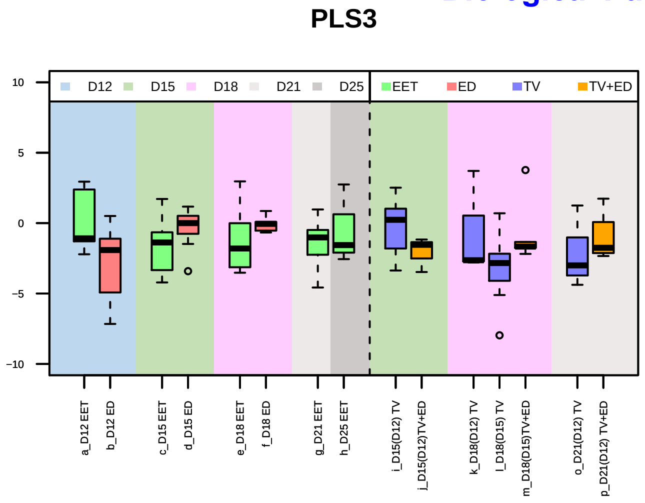

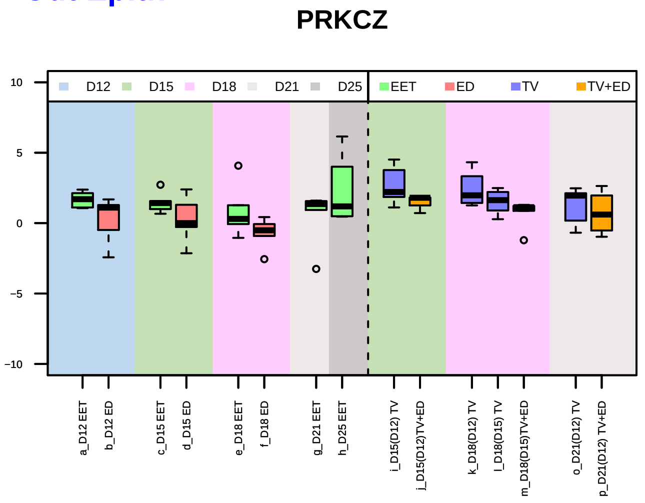

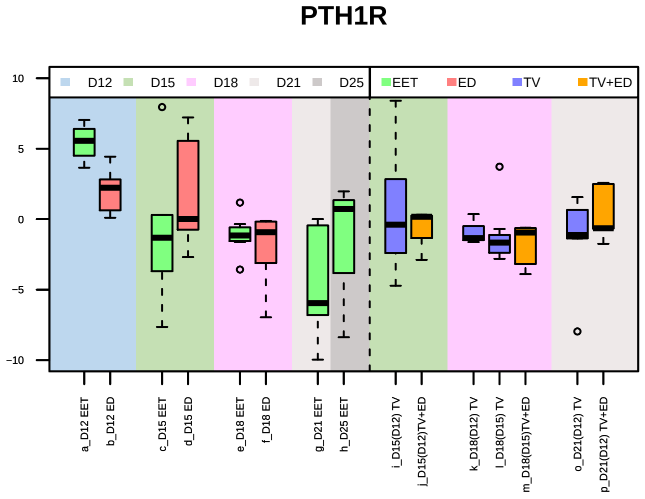

Biological Pathway = Short gut

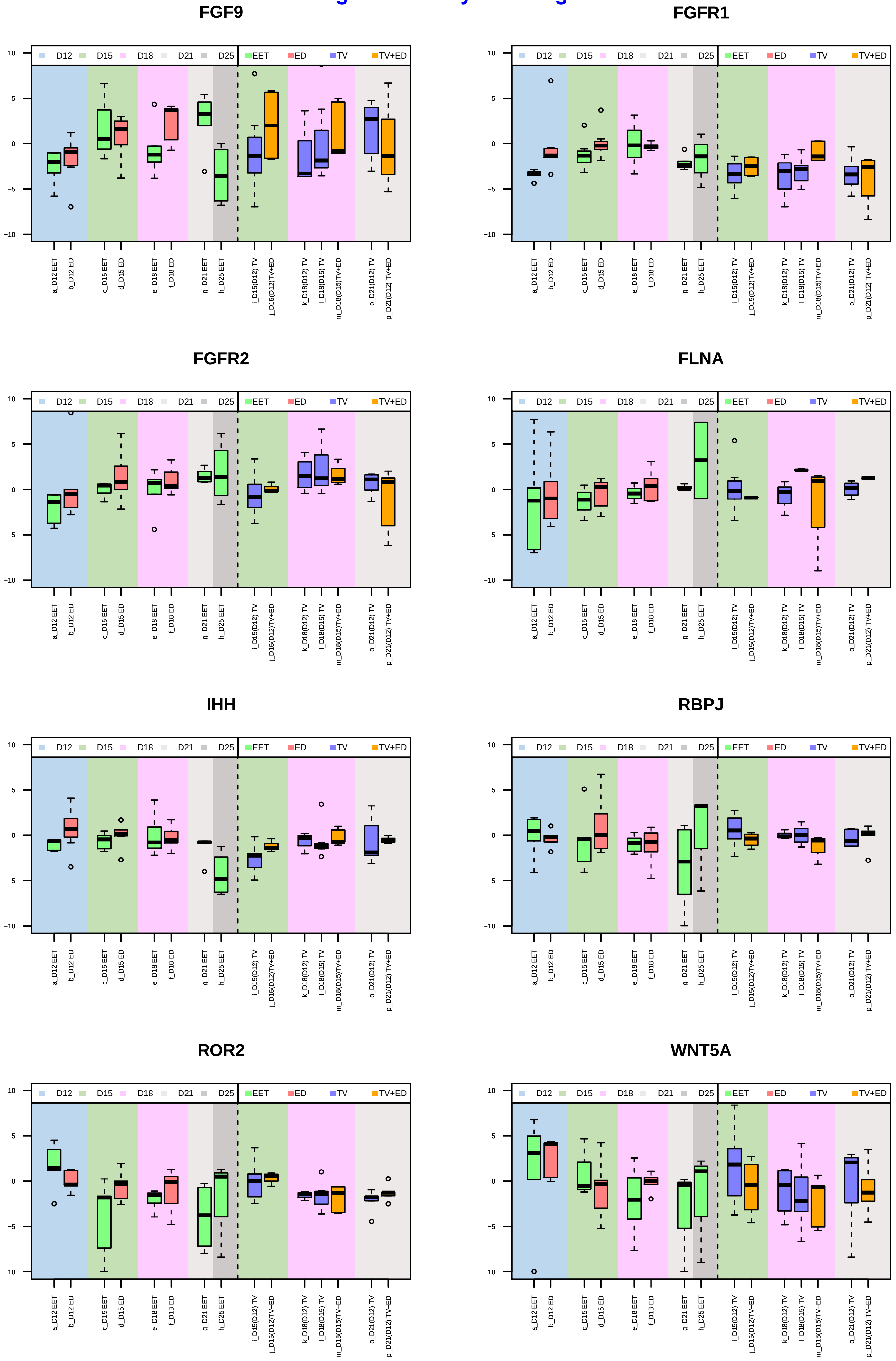

Biological Pathway = Tissue repair

ANGPT1

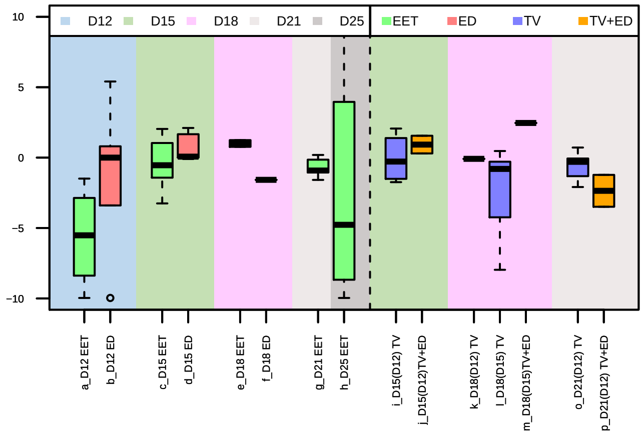

ANGPT2

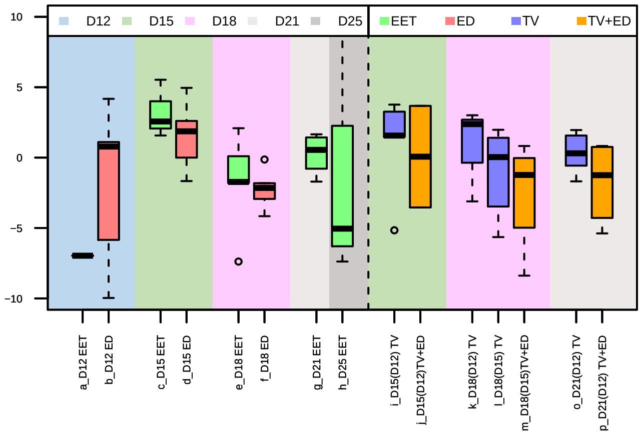

ANXA1

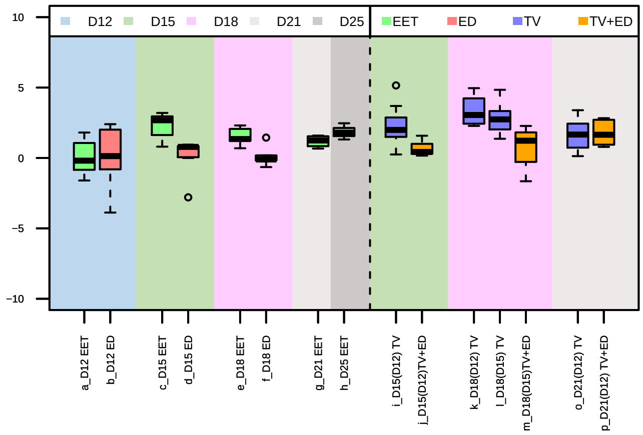

ANXA5

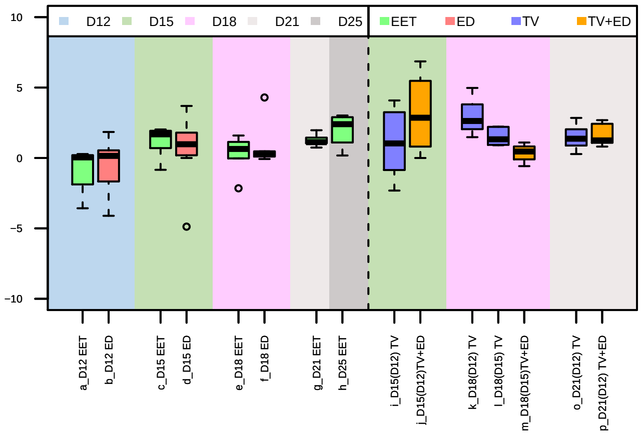

CLDN4

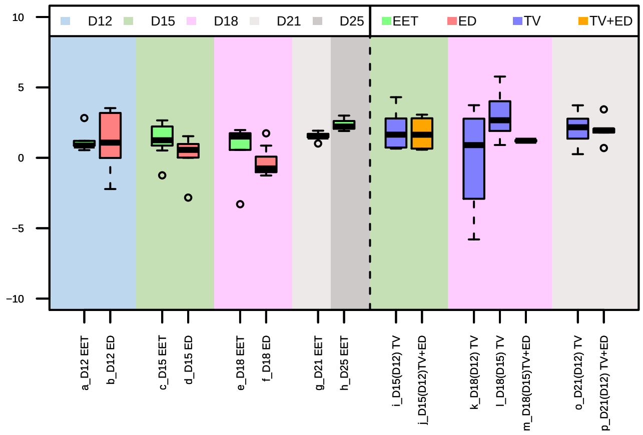

DCN

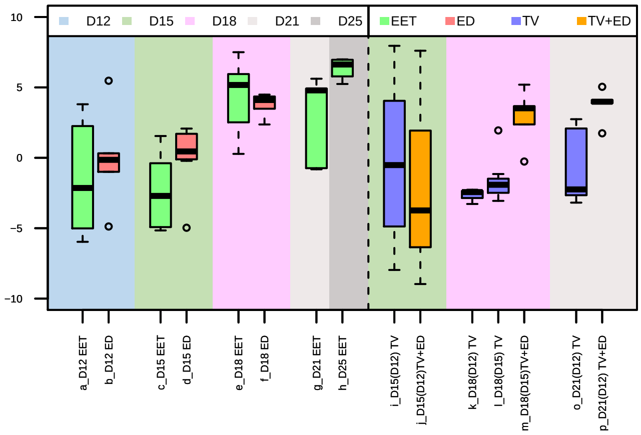

DKK1

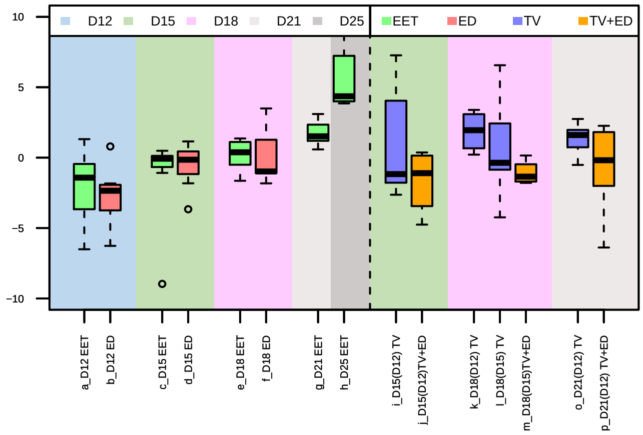

HSPG2

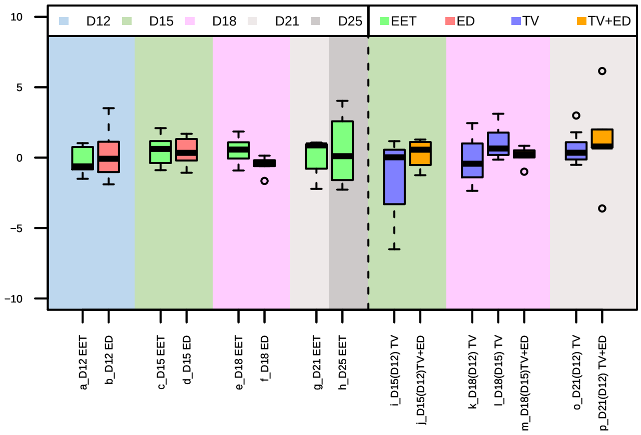

Biological Pathway = Tissue_repair

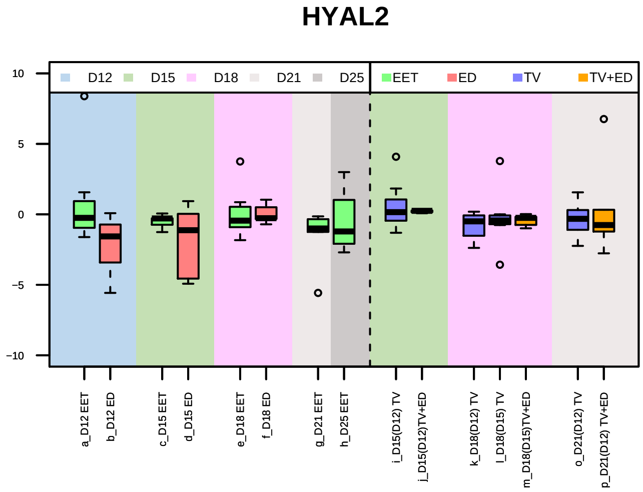

Biological Pathway = HFIsEmb

DMRTA1

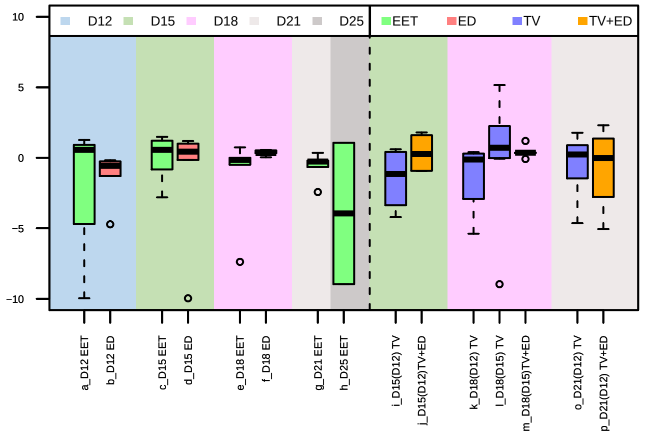

FKBP9

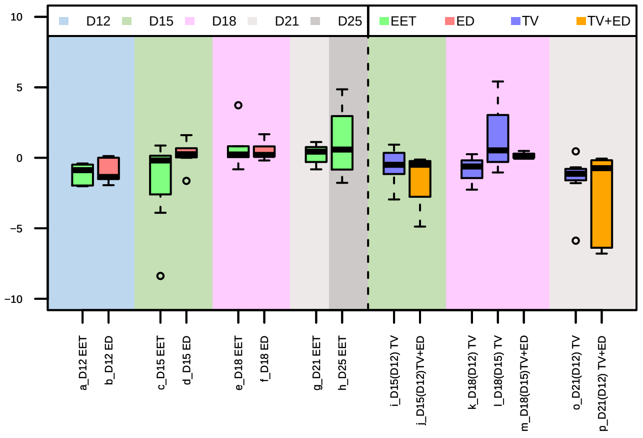

GPRC5B

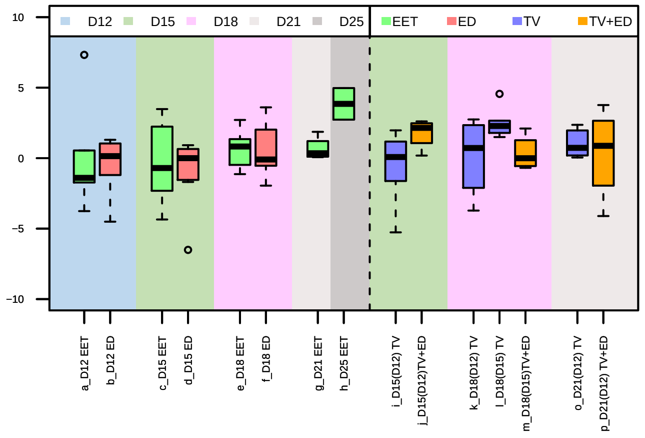

HOXA10

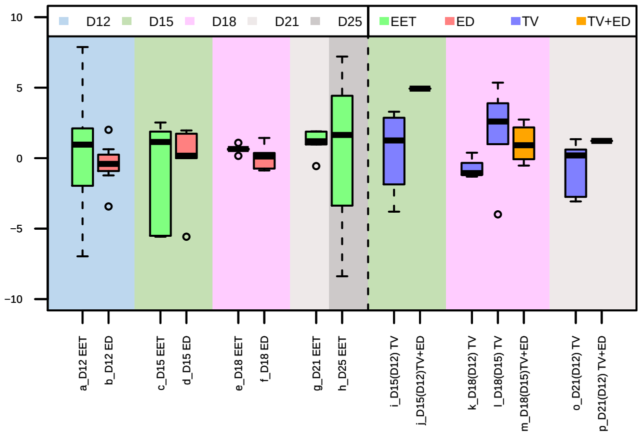

PTGER1

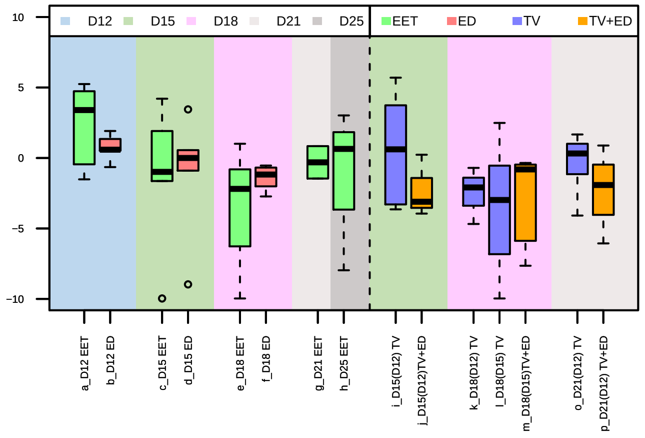

Biological Pathway = Discrimants

CALM1

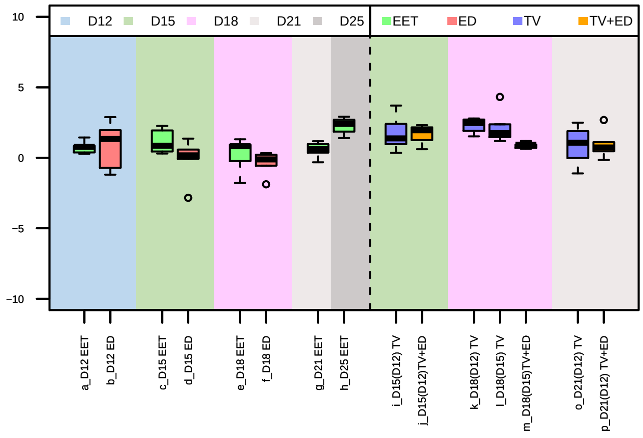

CPA3

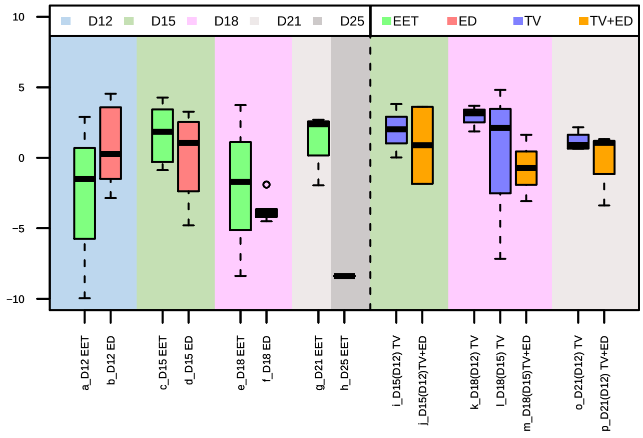

DLD

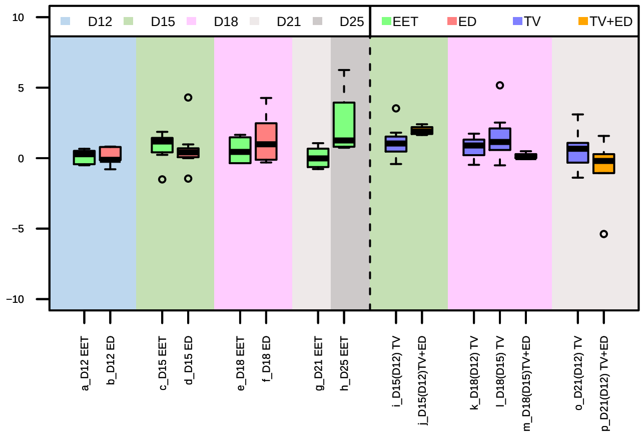

TGFB3

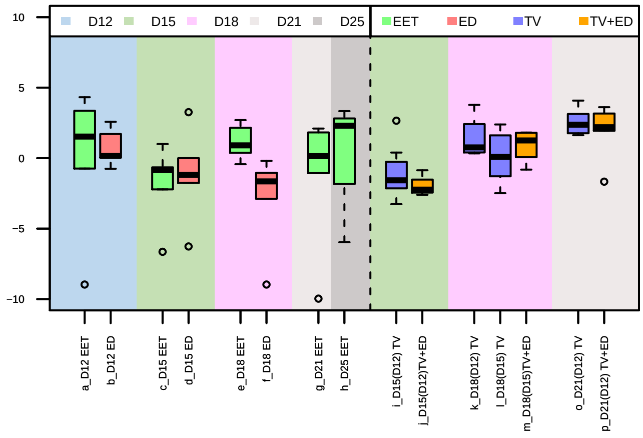

Biological Pathway = Housekeeping

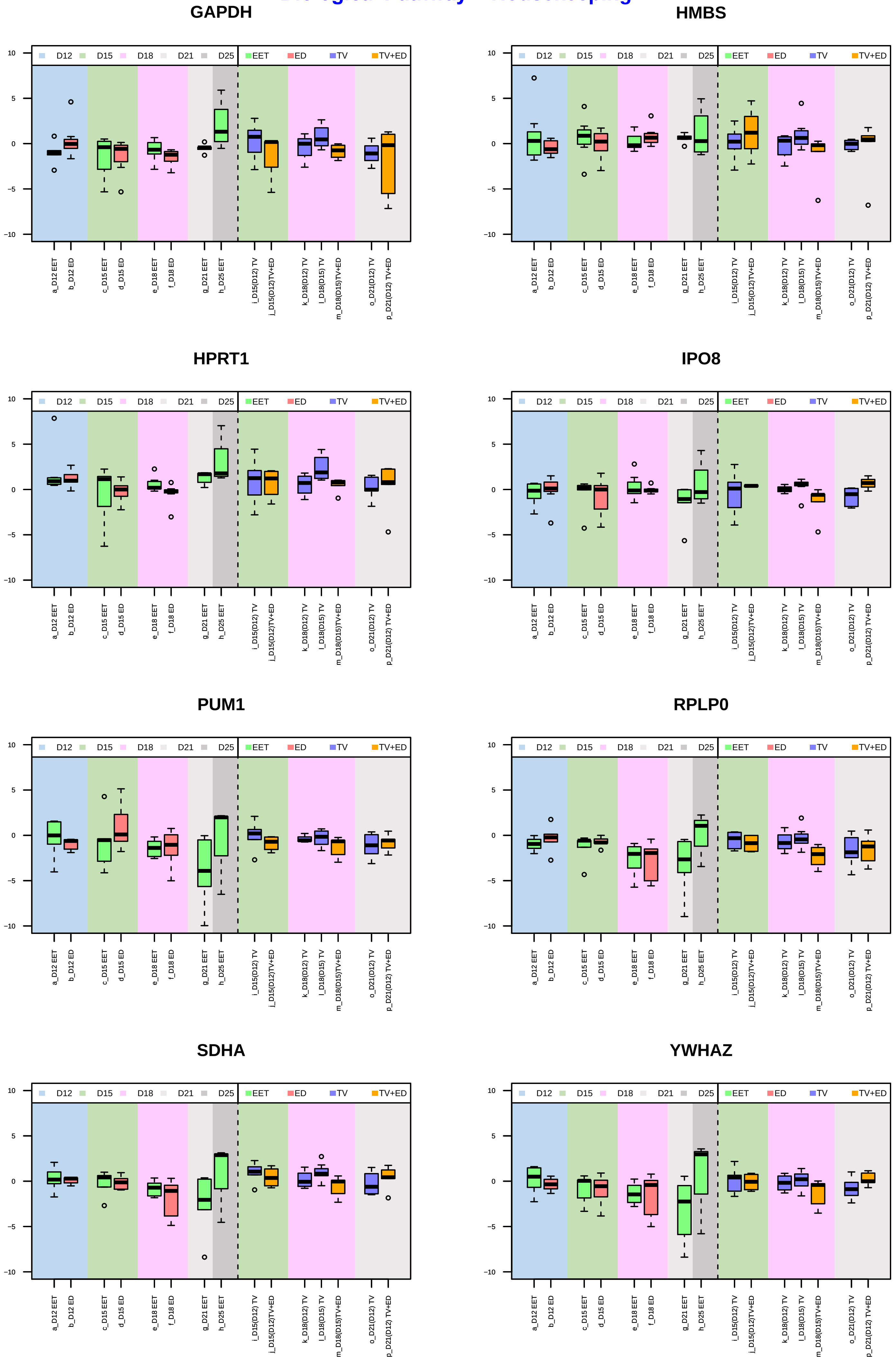

Supplement: Supplementary file 4 [file DataSheet3.PDF]
